# In vitro and in vivo single-agent efficacy of checkpoint kinase inhibition in acute lymphoblastic leukemia

**DOI:** 10.1186/s13045-015-0206-5

**Published:** 2015-11-05

**Authors:** Ilaria Iacobucci, Andrea Ghelli Luserna Di Rorà, Maria Vittoria Verga Falzacappa, Claudio Agostinelli, Enrico Derenzini, Anna Ferrari, Cristina Papayannidis, Annalisa Lonetti, Simona Righi, Enrica Imbrogno, Silvia Pomella, Claudia Venturi, Viviana Guadagnuolo, Federica Cattina, Emanuela Ottaviani, Maria Chiara Abbenante, Antonella Vitale, Loredana Elia, Domenico Russo, Pier Luigi Zinzani, Stefano Pileri, Pier Giuseppe Pelicci, Giovanni Martinelli

**Affiliations:** Department of Experimental, Diagnostic and Specialty Medicine, Institute of Hematology “L. e A. Seragnoli”, University of Bologna, Bologna, Italy; Department of Experimental Oncology, European Institute of Oncology, Milan, Italy; Department of Biomedical and Neuromotor Sciences, University of Bologna, Bologna, Italy; Hematology and BMT Unit, University of Brescia, Brescia, Italy; Division of Hematology, Department of Cellular Biotechnologies and Hematology, “Sapienza” University of Rome, Rome, Italy

**Keywords:** Acute lymphoblastic leukemia, DNA damage, Checkpoint kinase, Drug-sensitivity, New targets

## Abstract

**Background:**

Although progress in children, in adults, ALL still carries a dismal outcome. Here, we explored the in vitro and in vivo activity of PF-00477736 (Pfizer), a potent, selective ATP-competitive small-molecule inhibitor of checkpoint kinase 1 (Chk1) and with lower efficacy of checkpoint kinase 2 (Chk2).

**Methods:**

The effectiveness of PF-00477736 as single agent in B-/T-ALL was evaluated in vitro and in vivo studies as a single agent. The efficacy of the compound in terms of cytotoxicity, induction of apoptosis, and changes in gene and protein expression was assessed using different B-/T-ALL cell lines. Finally, the action of PF-00477736 was assessed in vivo using leukemic mouse generated by a single administration of the tumorigenic agent n-ethyl-n-nitrosourea.

**Results:**

Chk1 and Chk2 are overexpressed concomitant with the presence of genetic damage as suggested by the nuclear labeling for γ-H2A.X (Ser139) in 68 % of ALL patients. In human B- and T-ALL cell lines, inhibition of Chk1/2 as a single treatment strategy efficiently triggered the Chk1-Cdc25-Cdc2 pathway resulting in a dose- and time-dependent cytotoxicity, induction of apoptosis, and increased DNA damage. Moreover, treatment with PF-00477736 showed efficacy ex vivo in primary leukemic blasts separated from 14 adult ALL patients and in vivo in mice transplanted with T-ALL, arguing in favor of its future clinical evaluation in leukemia.

**Conclusions:**

In vitro, ex vivo, and in vivo results support the inhibition of Chk1 as a new therapeutic strategy in acute lymphoblastic leukemia, and they provide a strong rationale for its future clinical investigation.

**Electronic supplementary material:**

The online version of this article (doi:10.1186/s13045-015-0206-5) contains supplementary material, which is available to authorized users.

## Background

Acute lymphoblastic leukemia represents a biologically and clinically heterogeneous group of B/T-precursor-stage lymphoid cell malignancies arising from genetic insults that block lymphoid differentiation and drive aberrant cell proliferation and survival. Survival rates for children are approximately 80–85 % with current risk-oriented treatment protocols in contrast to less than 40 % for adults [1–3]. Although the outcome of children with relapsed ALL is heterogeneous, many achieve lasting second remissions. In contrast, survival after relapse in adult ALL is short [4, 5]. New therapeutic strategies are therefore needed to improve remission rates in adults and to prevent relapse both in adult and pediatric ALL patients.

Recently, in order to enhance DNA-damaging effects inflicted by cytotoxic drugs or radiation, different checkpoint kinase (Chk) inhibitors have been developed and assessed alone or in combination with DNA-damaging agents in preclinical studies and in phase I/II trials for cancer therapy [6–14]. Some of them inhibit both Chk1 and Chk2; others have a higher specificity for Chk1. The biological background underlying the development of Chk1 inhibitors and preliminary data deriving from early clinical trials have been recently well reviewed in [15]. Following DNA damage, multiprotein complexes recruit the transducers ataxia telangiectasia mutated (ATM) and ataxia telangiectasia and Rad3-related protein (ATR) which activate Chk2 and Chk1, respectively, despite an existing extensive crosstalk between the ATR-Chk1 and ATM-Chk2 pathways. Once activated by phosphorylation at S317/S345 in response to DNA single strand breaks, Chk1 phosphorylates the phosphatase Cdc25A at the G1/S and S phase checkpoints preventing cells from entering S phase and Cdc25A and Cdc25C at the G2/M checkpoint avoiding the entry in mitosis [16–19]. If Chk1 is induced by single strand breaks, Chk2 activation is largely restricted to DNA double strand breaks via ATM [20]. Once activated, they mediate cell cycle delay, DNA repair, and apoptosis in response to DNA damage. Moreover, Chk1 is required for proper mitotic spindle assembly and maintenance of chromosomal stability during mitosis [21].

PF-00477736 is a potent, selective, ATP-competitive, small-molecule inhibitor of Chk1, synthesized by Pfizer, which is selective for Chk1 and shows general selectivity over other kinases [6]. Preclinically, PF-00477736 enhanced docetaxel activity in tumor cells and xenografts by abrogating the mitotic spindle checkpoint, as well as the DNA damage checkpoint [10]. The checkpoint abrogating and cytotoxic activities attributed to PF-00477736 in combination with chemotherapy agents (e.g., gemcitabine and carboplatin) showed selectivity for p53-defective cancer cell lines over p53-competent normal cells in vitro [6]*.* In xenografts, PF-00477736 enhanced the antitumor activity of gemcitabine in a dose-dependent manner. PF-00477736 combinations were well tolerated with no exacerbation of side effects commonly associated with cytotoxic agents [6].

In this study, we investigated the activity of PF-0477736, as a single agent, in B-/T-ALL by the following: assessment of the in vitro efficacy in ALL cell lines and primary blast cells, assessment of the in vivo efficacy in mouse models, and identification and validation of potential biomarkers of functional inhibition. Results demonstrated that in vitro treatment of B-/T-ALL cell lines and primary blast cells with PF-0477736 resulted in inhibition of cell viability, induction of DNA damage, and apoptosis. Moreover, in vivo studies confirmed the efficacy of Chk1 inhibition, suggesting that this therapeutic strategy may be promising in leukemia.

## Results

### Chk1 and Chk2 are overexpressed in acute lymphoblastic leukemia

**Fig. 1:**
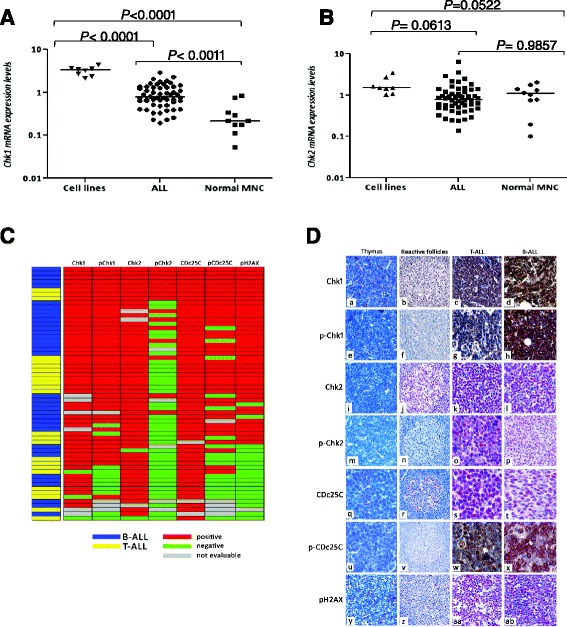
Chk1 **(a)** and Chk2 **(b)** mRNA expression levels in leukemia cell lines (BV-173, SUP-B15, REH, NALM-6, NALM-19, MOLT-4, RPMI-8402, and CCRF-CEM), blast cells from 54 adult newly-diagnosed ALL cases, and in normal bone marrow mononuclear cells (MNC). Results are expressed as Log10 2exp[−(ΔΔCt)], and they are the mean of at least two different replicates. **c** Immunohistochemical profile of the 60 ALL (36 B-ALL and 24 T-ALL); a *red box* indicates expression of the marker, whereas a *green box* signifies negativity (samples were considered positive if 30 % or more of the cells were stained with an antibody); *gray box* indicates a not evaluable reaction due to a core loss in TMA. The figure allows the assessment of the co-expression of the DNA damage markers in individual samples. **d** Immunohistochemical expression of Chk1, phosphorylated Chk1 (Ser345), Chk2, phosphorylated Chk2 (Thr68), and phosphorylated H2A.X (Ser139) (γ-H2A.X) in thymus, reactive follicle (RF), T-ALL and B-ALL. The figure highlights the close similarity between expression profiling of B- and T-ALL, characterized by a high percentage of cases Chk1^+^, pChk1^+^, Chk2^+^, Cdc25C, pCdc25C, pH2A.X^+^ and, in a lesser extent, pChk2^+^, and its distinction with the expression profiling of the non-tumoral populations as maturing thymocytes, which were substantially negative for these seven proteins and as B lymphocytes of reactive follicles, which resulted Chk1 weakly ^+^, Chk2^+^, Cdc25C^+^ but negative for the corresponding phosphorylated forms and pH2A.X. (*a*) Thymus: thymocytes Chk1^−^ (×400); (*b*) RF: B lymphocytes in mantle zone (*MZ*) and germinal center (*GC*) showing weak Chk1 positivity (×100); (*c*) strong expression of Chk1 in T-ALL; and (*d*) B-ALL (×400). (*e*) Thymus: thymocytes pChk1^−^ (×400); (*f*) RF: B lymphocytes in MZ and GC pChk1^−^; (*g*) strong positivity of pChk1 in T-ALL; and (*h*) B-ALL (×400). (*i*) Thymus: normal lymphoblasts Chk2^−^ (×400); (*j*) RF: B lymphocytes in MZ and GC showing diffuse Chk2 positivity (×100); (*k*) strong nuclear staining for Chk2 in T-ALL; and (*l*) B-ALL (×400). (*m*) Thymus: thymocytes pChk2^−^ (×400); (*n*) RF: B lymphocytes in MZ and GC pChk2^−^ (×100); (*o*) diffuse expression of pChk2 in T-ALL; and (*p*) B-ALL (×400). (*q*) Thymus: normal lymphoblasts negative for Cdc25C (×400); (*r*) RF: B lymphocytes in mantle zone (MZ) and germinal center (GC) showing weak Cdc25C positivity (×100); (*s*) strong nuclear staining for Cdc25C in T-ALL; and (*t*) B-ALL (×400). (*u*) Thymus: thymocytes pCdc25C^−^ (×400); (*v*) RF: B lymphocytes in MZ and GC pCdc25C^−^ (×100); (*w*) diffuse expression of pCdc25C in T-ALL; and (*x*) B-ALL (×400); (*y*) Thymus: normal lymphoblasts negative for pH2A.X (γ-H2A.X) (×400); (*z*) RF: MZ B and GC lymphocytes substantially pH2A.X (γ-H2A.X)-negative, with only occasional GC positive cells (×100); (*aa*) strong nuclear staining for pH2A.X (γ-H2A.X) in T-ALL; and (*ab*) B-ALL (×400)

Chk1/2 transcript levels were assessed by quantitative polymerase chain reaction (qPCR) in eight cell lines from B/T-ALL (BV-173, SUP-B15, REH, NALM-6, NALM-19, MOLT-4, RPMI-8402, and CCRF-CEM) (Additional file 1: Figure S1) and in blast cells from 54 adult newly diagnosed ALL cases including 41 (76 %) *BCR-ABL1*-positive and 13 (23 %) *BCR-ABL1*-negative cases. Normal bone marrow precursor cells isolated from leukemia patients in complete remission were also analyzed by the same method. In this cohort, higher transcript levels of Chk1 but not Chk2 were found in leukemia cell lines and newly diagnosed ALL cases compared to normal bone marrow mononuclear cells (*p* value <0.001) (Fig. 1). The web-based public database Oncomine [22] (https://www.oncomine.org/) was queried for Chk1/2 expression in the available leukemia datasets based on the comparison leukemia versus normal using a criterion of a twofold change for both Chk1 and Chk2 expressions and a *p* value of 1 × 10^−4^. Using these stringent criteria, we found that both Chk1 and Chk2 transcripts are highly overexpressed in B-ALL and T-ALL if compared to normal bone marrow samples (Additional file 1: Figure S2). We then investigated by immunohistochemistry of formalin-fixed paraffin-embedded (FFPE) tissue samples collected at diagnosis from 60 ALL patients (36 B-ALL and 24 T-ALL) for protein expression of Chk1, phosphorylated Chk1 (Ser345), Chk2, phosphorylated Chk2 (Thr68), Cdc25C, phosphorylated Cdc25C (Ser 2016), and phosphorylated H2A.X (Ser139) (γ-H2A.X). Results are detailed in Table 1 and viewed in Fig. 1c. Among B/T-ALL, a diffuse positivity for Chk1, Chk2, Cdc25c, and the phosphorylated forms of Chk1 (Ser345) and Cdc25c (Ser216) was observed; these were detected in 51/54 (96 %), 55/57 (94 %), 57/57 (100 %), 45/56 (80 %), and 38/55 (70 %) of the cases, respectively, whereas 15/55 (27 %) of ALLs was stained for phosphorylated form of Chk2 (Thr68) (Fig. 1d). Interestingly, in our ALL series, genomic damage was suggested by the nuclear labeling for γ-H2A.X molecule in 40/59 (68 %) of samples (Fig. 1d). In thymuses, normal lymphoblasts did not show protein expression of Chk1, Chk2, Cdc25C, and their phosphorylated counterparts (Fig. 1d). In B follicles of the reactive lymph node, in spite of the Chk1 weak positivity, diffuse Chk2, and Cdc25C nuclear staining, no phosphorylation in their amino acid Ser345, Thr68, and Ser216, respectively, was detected (Fig. 1d). Notably, in thymuses and reactive B follicles, only scattered γ-H2A.X^+^ cells were present, indicating a low level of genomic damage in physiologic conditions compared to ALLs (Fig. 1d).

**Table 1:** Immunohistochemical results

Tumor type	Chk1		pChk1		Chk2		pChk2		Cdc25C		pCDc25C		γH2A.X	
	pos	(%)	pos	(%)	pos	(%)	pos	(%)	pos	(%)	Pos	(%)	pos	(%)
ALL	51/54	(94)	45/56	(80)	55/57	(96)	15/55	(27)	57/57	(100)	38/55	(70)	40/59	(68)
B-ALL	31/31	(100)	28/33	(85)	32/33	(97)	11/32	(33)	34/34	(100)	23/32	(72)	25/36	(69)
T-ALL	20/23	(87)	17/23	(74)	23/24	(96)	4/23	(17)	23/23	(100)	15/23	(65)	15/23	(65)

*pos* number of positive cases/number of evaluable cases

### PF-00477736 reduces cell viability in a dose-dependent manner

**Fig. 2:**
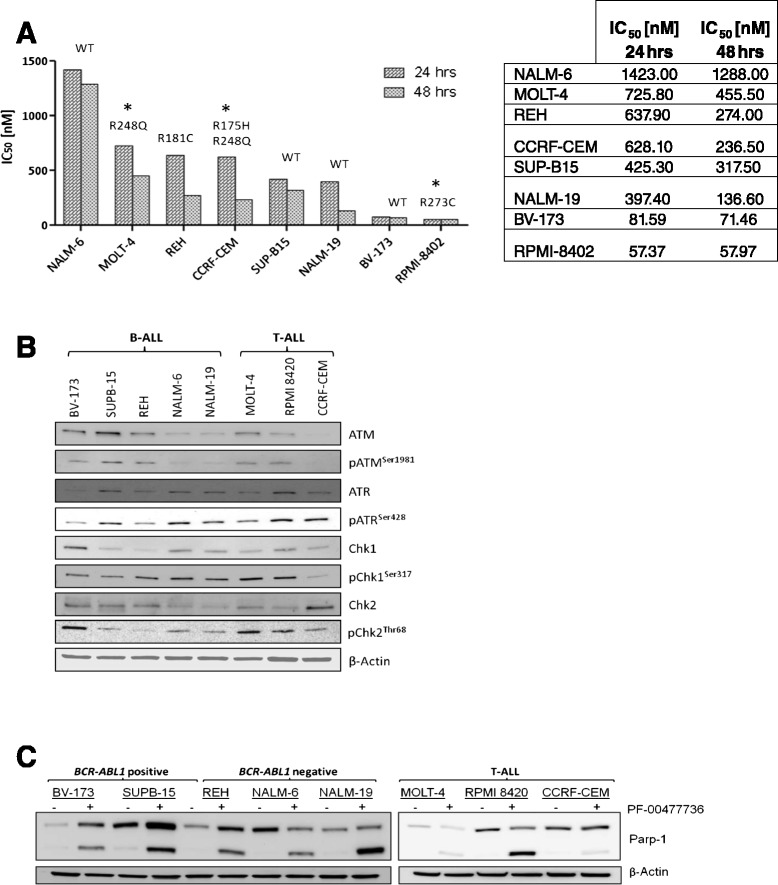
**a** IC_50_ values for B- and T-ALL cell lines at exposure durations of 24 and 48 h (*hrs*) to PF-0477736. For each cell line, the mutational status of *TP53* gene is shown. Abbreviations: *WT* wild-type; *hrs* hours. **b** Assessment of the expression and activation of the major components of the ATR-Chk1 and ATM-Chk2 pathways in B-/T-ALL cell lines at baseline. The homogeneity of the protein loaded was determined by using as an internal control (β-actin). **c** Reduction of PARP-1 cleavage in leukemia cell lines detected by Western blotting. PARP-1 cleavage was detected as a marker of apoptosis. Β-actin was detected on the same membrane for loading normalization

B-/T-ALL cell lines were incubated with increasing concentrations of drug (5–1000 nM) for 24 and 48 h. PF-00477736 inhibition resulted in dose- and time-dependent cytotoxicity with RPMI-8402 (T-ALL) being the most sensitive (IC_50_ = 57.4 nM at 24 h) while NALM-6 (B-ALL) the most resistant (IC_50_ = 1423.0 nM at 24 h). In vitro sensitivity does not correlate with leukemia cell type (B-ALL vs T-ALL), *TP53* mutation status (BV-173, SUPB-15, NALM-6, and NALM-19 cells were p53 wild-type, whereas REH, MOLT-4, RPMI-8402, and CEM cells were p53 mutated) (Fig. 2a), and with baseline levels of Chk1/2 and ATR/ATM phosphorylation, indicative of intrinsic genetic stress (Fig. 2b).

### PF-00477736 induces apoptosis at 24 and 48 h in B-/T-ALL cells

In order to assess whether cytotoxicity was correlated to increased susceptibility to apoptosis, B-/T-ALL cell lines were incubated with increasing concentrations of drug (0.1, 0.5, and 1 μM and 0.05, 0.1, and 0.2 μM only for BV-173 and RPMI-8402, respectively) for 24 and 48 h. Consistent with the viability results, Annexin V/propidium iodide (PI) staining analysis showed a significant increase of apoptosis at 24 and 48 h in B- and T-ALL cells proportional to drug dose and drug exposure times (Additional file 1: Figure S3-A). The induction of apoptosis by PF-00477736 was also assessed by the detection of poly (ADP-ribose) polymerase (PARP) cleavage by Western blot analysis [23]. The PARP-1 cleavage band at 89 kDa was observed in lysates from leukemia cell lines after 24 h of drug exposure, while it was undetectable in cells treated with only DMSO 0.1 % (Fig. 2c).

### PF-00477736 perturbs the cell cycle profile in B-/T-ALL cells

The effect of the inhibition of Chk1 pathway on cell cycle progression was evaluated in RPMI-8402, BV-173, SUP-B15, and NALM-6 cell lines. Cells were incubated with increasing concentrations of PF-00477736 for 6 and 24 h and then stained with propidium iodide to quantify the DNA amount. The effect of PF-00477736 on the cell cycle progression was very weak after 6 h of incubation (data not showed) and very heterogeneous among the different cell lines after 24 h. In RPMI-8402, BV-173, and SUP-B15 cell lines, the treatment induced a progressive reduction of the number of cells in S and G2/M phase and a concomitant increment of cell debris. In the less sensitive cell line, NALM-6, the inhibition of Chk1 progressively increased the percentage of cells in G2/M phase and reduced the percentage of cells in G1 phase (Additional file 1: Figure S3-B).

### PF-00477736 efficiently targets Chk1 pathway and induces DNA damage

**Fig. 3:**
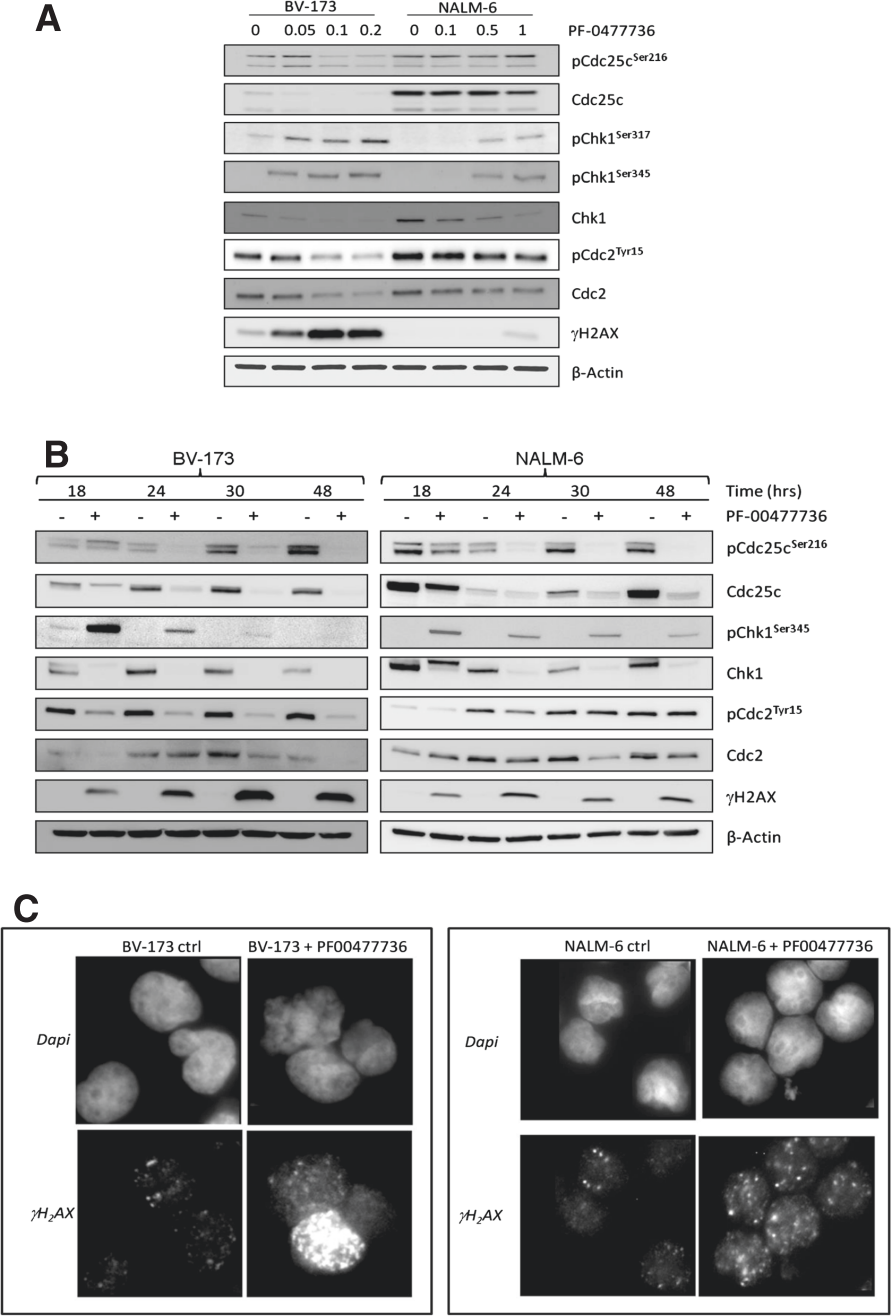
**a** Western blot analysis in BV-173 and NALM-6 cell lines after exposure to increasing concentrations of PF-00477736 (0.05, 0.1, and 0.2 uM for BV-173 and 0.1, 0.5, and 1 uM for NALM-6) or DMSO 0.1 % (−). **b** Western blot analysis in BV-173 and NALM-6 cell lines after exposure to PF-00477736 (+) at the concentration closest the IC_50_ or DMSO 0.1 % (−) at 18, 24, 30, and 48 h. **c** Western blot analysis in all leukemia cell lines after exposure to PF-00477736 (+) at the concentration closest the IC_50_ or DMSO 0.1 %

In order to assess whether PF-00477736 efficiently targeted Chk1 pathway, we examined the changes in the downstream phosphorylation of the phosphatase Cdc25C and cyclin-dependent kinase, Cdc2. Moreover, Chk1 itself and γ-H2A.X, which is a marker of stalling replication forks and checkpoint abrogation-induced apoptosis, have been assessed. Functional analyses were initially performed on the most sensitive (BV-173) and the most resistant (NALM-6) B-ALL cell lines, as determined by the viability and apoptosis analyses. Specifically, BV-173 cells were treated with 0.05, 0.1, and 0.2 μM and NALM-6 with 0.1, 0.5, and 1 μM of PF-00477736 for 24 h (Fig. 3a). In the second analysis, BV-173 and NALM-6 cells were treated with the dose of PF-00477736 that goes near to the IC_50_ (0.1 μM for BV-173 and 1 μM for NALM-6) for 18, 24, 30, and 48 h (Fig. 3b). Thereafter, the same molecular targets have been assessed on all B- and T-ALL cell lines using the dose that goes near to the IC_50_ (Additional file 1: Figure S4). Active Chk1 phosphorylates Cdc25C at serine 216 throughout the interphase and upon G2 checkpoint activation. This leads to the nuclear export of Cdc25C and its subsequent cytoplasmic sequestration by 14-3-3 protein, which prevents the activation of the downstream target of Cdc25C, the cyclin B/Cdc2 kinase that is responsible for G2/M transition [24]. Upon inhibition of Chk1, we observed decreased levels of phosphorylated Cdc25c (Ser216) and Cdc2 (Tyr 15) as well as reduced levels of their total forms in a dose- and time-dependent manner, suggesting that PF-00477736 efficiently targets Chk1 pathway. In contrast, the phosphorylated forms of Chk1 (Ser317 and Ser345) were dose-dependently increased suggesting that treatment amplifies genomic damage which in a feedback loop increases Chk1 phosphorylation. Although the increment of the phosphorylated forms of Chk1 (Ser317 and Ser345) as a consequence of genomic damage amplification, the complete inhibition of Chk1 functionality was confirmed by the progressive reduction of the auto-phosphorylated form of Chk1 (Ser296) in an early time point analysis. Indeed, even after 3 h of treatment with PF-00477736 in both BV-173 and NALM-6 cell lines, the auto-phosphorylated form of Chk1 (Ser296) was drastically reduced in comparison with the untreated counterpart (Additional file 1: Figure S4-B). The enhancement of genomic damage was also confirmed by the time-dependently increase of γ-H2A.X both in Western blot and immunofluorescence analyses.

The phospho-H2A.X accumulates and forms characteristic nuclear foci where the DNA is damaged [25]. Both NALM-6 and BV-173 cell lines show an increased number of γ-H2A.X foci when treated with PF-00477736 compared to untreated controls (Fig. 3c). However, evaluating the mean fluorescence intensity of γ-H2A.X positive cells, BV-173 cells seem to be more damaged than NALM-6. Actually, BV-173 treated cells have five-fold change difference of mean value intensity of γ-H2A.X compared to BV-173 untreated cells, while NALM-6 treated cells have 1.4-fold change difference of mean value intensity of γ-H2A.X compared to their untreated counterparts. In addition to these changes in the overall amount of γ-H2A.X foci among the two different cell types, we observed the presence of hyper-γ-H2A.X-positive cells specifically in BV-173 cells treated with PF-00477736. The so-called hyper-γ-H2A.X-positive cells loose the typical foci signal of γ-H2A.X and emit a much higher and diffuse signal of γ-H2A.X positivity. Almost 6.5 % of the γ-H2A.X-positive cells are hyper-γ-H2A.X-positive in BV-176-treated cells compared to 1.8 % in the untreated controls. These data suggest a higher induction of DNA damage upon PF-00477736 treatment in BV-173 than NALM-6 cells as expected by the fact that BV-173 cells are more responsive to PF-00477736. However, the increase of hyper-γ-H2A.X-positive cells only in BV-176-treated cells would suggest the existence of a specific effect that causes a hyper DNA damage only in a restricted, but significant, population of leukemic cells.

Interestingly, upon treatment, we observed a strong reduction of Chk1. As already hypothesized, this may be the result of cleavage by caspase during apoptosis induced by genotoxic stress [26, 27]. Persisting protein levels of Cdc25, pChk1, Ser345, pCdc2 (Tyr 15), and Cdc2 after 48 h of exposure to PF-00477736 differentiated less sensitive leukemia cells from more sensitive ones (Additional file 1: Figure S3c).

### Gene expression profiling results

**Fig. 4:**
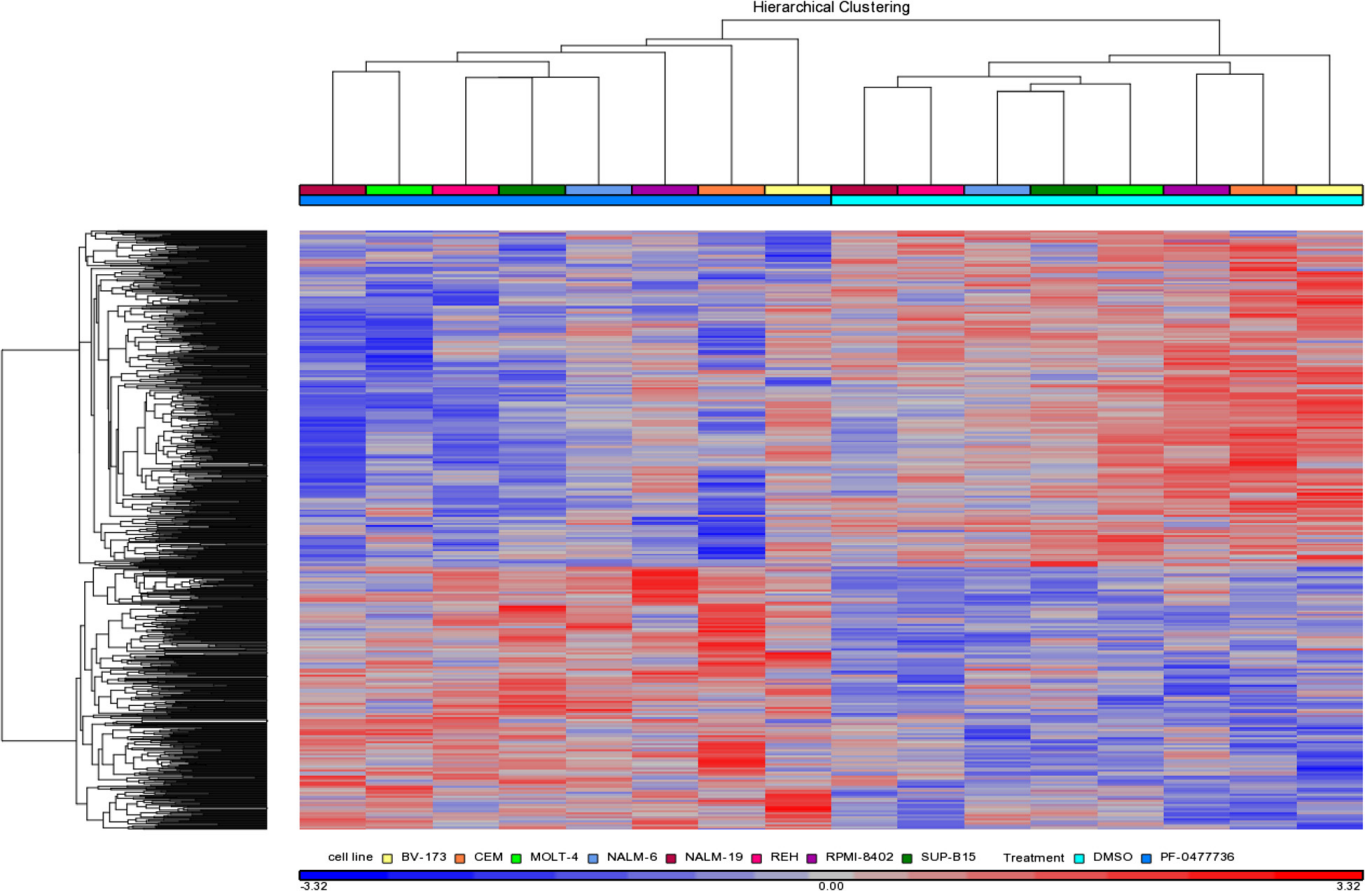
Heat map of differentially expressed genes. Lists of differentially expressed genes with a *t* test *p* value <0.05 were generated for each cell line. A hierarchical clustering method in Partek Genomics Suite was used to construct both the gene tree and the sample tree. Data are shown in a matrix format: each row represents a single gene, and each column represents a cell line. *Red* indicates overexpressed genes and *blue* indicates underexpressed genes (see legend)

In order to identify gene expression changes specifically correlated with Chk1 inhibition and to better elucidate the mechanism of action of Chk1 inhibitor, gene expression profiling (GEP) analysis was performed by microarray on treated B/T-ALL cell lines (BV-173, SUP-B15, REH, NALM-6, NALM-19, MOLT-4, RPMI-8402, and CCRF-CEM) and on their untreated counterparts (DMSO 0.1 %) after 24 h of drug exposure. Treatment resulted in a differential expression of 941 genes (*p* < 0.05): 528 (56 %) were down-modulated and 413 (44 %) were upregulated in treated leukemia cells compared to untreated cells (Additional file 2 and Fig. 4). To identify peculiar critical pathways affected by Chk1 inhibition, the differentially expressed genes were analyzed in terms of biological function using the MetaCore pathway mapping software (GeneGo Inc.) which categorizes genes in pathway maps, Gene Ontology (GO) cellular processes, and cellular and molecular process networks. Consistent with a Chk1’s mechanism of action, the three top scored maps (map with the lowest *p* value) were “cell cycle: estrogen receptor 1 (ESR1) regulation of G1/S transition” including *c-Jun/c-Fos, cyclin A*, *CARM1*, Skp2/TrCP/*FBXW*, *SKP2*, c-Fos, *CDK4, NCOA3, c-Jun,* and *CDK2* genes (Additional file 1: Figure S5); “apoptosis and survival: granzyme A signaling” including *PHAP1*, *Ku70/80, Ku80, Ku70, histone H3, NDPK A, SET*, histone H2B, and histone H1 genes (Additional file 1: Figure S6); and “DNA damage: ATM/ATR regulation of G1/S checkpoint” including *PCNA,* growth arrest and DNA damage 45 alpha (*GADD45a*)*, Chk2, cyclin A, NF-kB,* cyclin-dependent kinase 4 (*CDK4), FANCD2, Claspin*, and *CDK2* genes (Additional file 1: Figure S7). GO cellular processes highlighted 617 genes involved in cellular metabolic process (Additional file 3). Finally, the three top scored process networks impaired by the treatment with Chk1 inhibitor were as follows: the DNA damage checkpoint, the S phase of cell cycle, and the apoptosis, confirming the specificity of treatment on its target pathways (Additional file 4). Thirty-five genes had a false discovery rate less than 0.05 (Additional file 5: Table S1), and the three most differentially expressed genes were as follows: DNA damage-inducible transcript 3 (*DDIT3*, fold-change 3.32, *p* value 6.06 × 10^−5^), Kruppel-like factor 6 (*KLF6,* fold-change 2.17, *p* value 8.41 × 10^−5^), and *FBJ* murine osteosarcoma viral oncogene homolog (*FOS*, fold-change 2.40, *p* value 1.97 × 10^−4^). Due to its implication in the regulation of the cell cycle and apoptosis, Western blot analysis against c-Jun was performed to better comprehend its biological role in response to PF-00477736. c-Jun is a component of the transcription factor activating protein-1 (AP-1) which is involved in cell cycle progression through the G1 phase by the regulation of the cyclin D1 [28]. As soon after treatment with Chk inhibitor, c-Jun protein levels increased in all B-ALL cell lines but not in all T-ALL cell lines in which the response to the treatment was very heterogeneous (Additional file 1: Figure S8-A). The data found in the gene expression profile analysis were validated using qPCR. Four genes (two upregulated, PLK3 and GADD45a; two downregulated, Chk2 and CDK4), which have been chosen on their biological relevance in the Chk1 pathway, were validated on B-/T-ALL cell lines treated for 24 h with or without PF-00477736 (IC50) (Additional file 1: Figure S8-C).

### PF-00477736 reduces viability in primary ALL blast cells

**Fig. 5:**
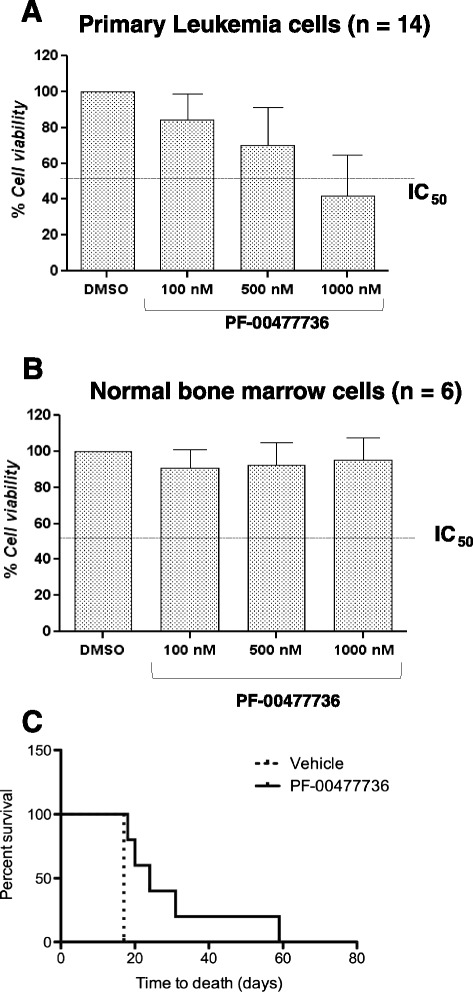
**a** Cell viability results in primary ALL blast cells after 24 h of exposure to PF-0477736 at 100, 500, and 1000 nM. **b** Cell viability results in normal bone marrow mononuclear cells after 24 h of exposure to PF-0477736 at 100, 500, and 1000 nM. **c** Survival curves of leukemic mice treated with PF-0477736 and control mice injected with vehicle. PF-0477736 treatment significantly increases the overall survival of mice transplanted with murine lymphoid leukemia (*p* value =0.0009)

The efficacy of PF-00477736 was confirmed in primary blast cells from 14 newly diagnosed cases including ten *BCR-ABL1*-positive (71 %) cases and four *BCR-ABL1*-negative ALL cases (29 %). Leukemic cells were incubated with increasing concentrations of drug (0.1, 0.5, and 1 μM) for 24 h. Based on the viability results, three groups of patients were identified: very good responders, 36 % (5/14) with IC_50_ at 24 h ranging from 0.1 to 0.5 μM; good responders, 43 % (6/14) with IC_50_ at 24 h ranging from 0.5 to 1 μM; and poor responders, 21 % (3/14) with IC_50_ at 24 h higher than 1 μM (Additional file 5: Table S2 and Fig. 5a). PF-00477736 did not reduce viability in primary cultures of normal bone marrow mononuclear cells, demonstrating that it selectively targets leukemia cells (Fig. 5b). Moreover, in contrast to leukemia cells, treatment did not induce protein phosphorylation changes in Chk1 (Ser345), Cdc25c (Ser216), and Cdc2 (Tyr 15) neither increased levels of phospho-H2A.X (Additional file 1: Figure S8-B).

### PF-00477736 impairs survival of leukemic mice

We extended the in vitro and ex-vivo studies by assessing the efficacy of Chk inhibitor in mice transplanted with T-ALL.

Leukemic mice were generated by the usage of the tumorigenic agent n-ethyl-n-nitrosourea (ENU). The obtained leukemia has been immunophenotyped and characterized as a T-ALL. Leukemic blasts coming from the spleen of leukemic animals were transplanted into C57BL/6Ly5.1-recipient mice. Three days after transplantation, a time sufficient for leukemic blasts to home the bone marrow of the host, we started to treat the animals. A concentration of 40 mg/kg of PF-00477736 given with a q3dx4 schedule seems not to be toxic for the animals; however, it significantly affects the overall survival of the treated animals (five mice) in comparison to the untreated ones (seven mice) (*p* value =0.0009). Actually, control animals (intraperitoneally injected with PBS) die all at day 17 post-transplantation while the animals treated with PF-00477736 live longer (up to 59 days post transplantation). The leukemia used for the experiments shown here was very aggressive (untreated animals die with massive infiltration of the spleen and the liver by leukemic blasts at day 17) suggesting a reason why the effect of the Chk inhibitor is significant though quite variable among treated mice (Fig. 5c).

## Discussion

Several studies in different tumors have investigated the role of Chk inhibition in combination with conventional chemotherapy demonstrating that this combination enhances tumor cell death [6, 10].

**Fig. 6:**
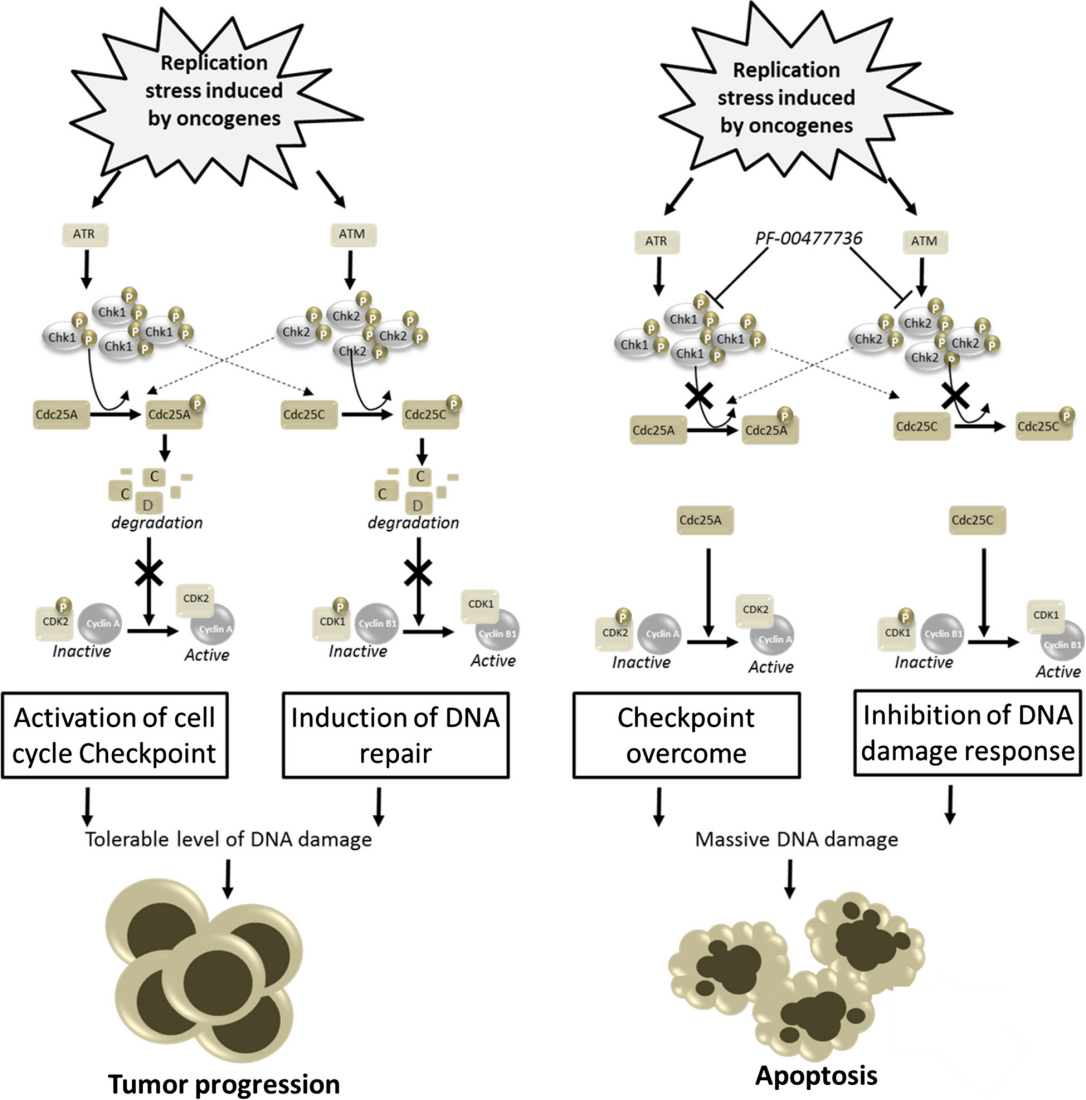
Cartoon drawing the rational of the study: in leukemia cells, oncogenes could lead to replication stress, activation of ATR/Chk1 pathway that in conjunction with elevated proliferation promote tolerable level of DNA damage, genomic instability, and tumor progression. Inhibition of Chk1/Chk2 could increase DNA damage leading to apoptosis

Here, we evaluated the in vitro and in vivo effects of the administration of PF-00477736 as a single agent and not in combination with other chemotherapeutic agents in cell lines and primary blast cells from acute lymphoblastic leukemia based on the hypothesis that the intrinsic genomic instability of leukemic clones as demonstrated by the nuclear labeling for γ-H2A.X molecule in 68 % of ALL patients may be itself sufficient to bring the cells to apoptosis. According to this hypothesis, it was possible to highlight how the administration of PF-0477736 as single agent was able to reduce cell viability in all leukemia cell lines treated in this study (BV-173, SUP-B15, REH, NALM-6, NALM-19, MOLT-4, RPMI-8402, and CCRF-CEM) and to induce apoptosis (Fig. 6). Different leukemia cell lines showed different sensitivity to PF-00477736 with RPMI-8402 (T-ALL) being the most sensitive (IC_50_ = 57.4 at 24 h) while NALM-6 (B-ALL) the less sensitive (IC_50_ = 1423.0 at 24 h) cell line. Interestingly, the sensitivity to Chk1 inhibitor was not related to the mutational status of the tumor suppressor p53.

The results obtained on cell viability and induction of apoptosis have been confirmed by genome-wide studies evaluating global gene expression changes upon treatment and by functional studies performed by Western blot analysis. Interestingly, by GEP analysis, the majority of genes downregulated were involved in the DNA damages response and in particular in DNA repair mechanisms, while the genes upregulated were involved in chromatin assembly, nucleosome organization, DNA packaging, and apoptosis. The efficacy of Chk inhibition has been evaluated and confirmed in terms of reduction of cell viability in primary ALL blast cells but not in normal bone marrow precursor cells. Furthermore, assessing the efficacy of Chk inhibition in mice transplanted with T-lymphoid leukemia, we demonstrated that PF-0477736 increases the survival of treated mice compared with mice treated with vehicle (*p* = 0.0016).

In conclusion, in vitro, ex-vivo, and in vivo results support the inhibition of Chk1 as a new therapeutic strategy in acute lymphoblastic leukemia, and they provide a strong rationale for its future clinical investigation. The checkpoint kinase inhibitor have been synthetized to increase the effectiveness of conventional chemotherapy, preventing cells to arrest cell cycle, and to repair the DNA damages caused by the exposure to genotoxic agents. Here, we highlight that even the treatment with a checkpoint kinase inhibitor alone associated with the high genetic instability can be enough to kill cancer cells. Indeed, we believe that leukemic cells, thanks to a higher activation of the ATR-Chk1/ATM-Chk2 pathways, can better tolerate high genetic instability. Switching off these mechanisms of survive, we can kill leukemic cell by overcoming the cell cycle checkpoint, by inhibiting the mechanisms of DNA damages repair and by inducing massive damages that cannot be tolerated (Fig. 6).

## Conclusions

Inhibition of Chk1/2 represents a novel therapeutic strategy to overcome genetic instability and to promote selective killing of leukemia cells in ALL.

## Methods

### Leukemia cell lines

Human B- (BV-173, SUP-B15, REH, NALM-6, and NALM-19), T-ALL (MOLT-4, RPMI-8402, and CCRF-CEM) cell lines were obtained from Leibniz-Institut DSMZ-Deutsche Sammlung von Mikroorganismen und Zellkulturen GmbH (Germany). Cells were cultured in RPMI-1640 medium (Invitrogen, Paisley, UK) with 1 % l-glutamine (Sigma, St. Louis, MO, USA) and penicillin and streptomycin (Gibco, Paisley, UK) supplemented with 10–20 % fetal bovine serum (Gibco) in a humidified atmosphere of 5 % CO2 at 37 °C. Online databases have been interrogated to molecularly characterize leukemia cell lines: International Agency for Research on Cancer (IARC) TP53 database (http://www-p53.iarc.fr/) and the Catalogue of Somatic Mutations in Cancer, (COSMIC, http://www.sanger.ac.uk/genetics/CGP/cosmic/). The Chk1 inhibitor PF-00477736 was purchased by Sigma-Aldrich (Sigma-Aldrich Co. St. Louis, MO, USA).

### Reagents

The Chk inhibitor PF-00477736 was purchased from Sigma-Aldrich (Sigma-Aldrich Co. St. Louis, Missouri 63103 United States). qPCR analysis of Chk1 and Chk2 mRNA expressions was evaluated in 41 (76 %) newly diagnosed BCR-ABL1-positive (median age 56 years, range 26–81; ratio male to female 20/21) and 13 (23 %) newly diagnosed BCR-ABL1-negative ALL cases (median age 41 years, range 18–69; ratio male to female 5/8). One microgram of RNA was reverse transcribed using the High-Capacity cDNA Archive Kit (Applied Biosystems, Foster City, CA, USA). PCR analysis was performed using HS00967506_m1 (Chk1) and HS00200485_m1 (Chk2) assays (Applied Biosystems) and the Fluidigm Dynamic Array 48 × 48 system, a real-time qPCR assay which enables to automatically assemble 48 samples and 48 assays to create individual TaqMan reactions of a final volume of 6.75 nl each (Fluidigm, San Francisco, CA, USA; http://www.fluidigm.com/). RNA integrity was confirmed by PCR amplification of the GAPDH mRNA (Hs99999905_m1), which is expressed ubiquitously in human hematopoietic cells. Results were expressed as 2exp(−ΔΔCt). GraphPad Prism 5 software (GraphPad, Avenida de la Playa La Jolla, CA, USA) was used to plot the data. The basal mRNA expression of Chk1 and of Chk2 was evaluated in all the cell lines using the same assays (Applied Biosystems: HS00967506_m1 Chk1 and HS00200485_m1 Chk2) used for the primary samples, and the statistical validity of the results were confirmed using ANOVA multiple comparisons test (GraphPad Prism 5 software). In addition, reverse transcription and quantitative PCR analysis for GADD45a, PLK3, CDK4, and CHK2 in treated cell lines and on their untreated counterpart was performed as described above using the following assays from Applied Biosystems: Hs00169255_m1 (GADD45a), Hs00177725_m1 (PLK3), Hs00262861_m1 (CDK4), and HS00200485_m1 (Chk2).

### TP53 mutation screening

Total cellular RNA was extracted using the RNeasy total RNA isolation kit (Qiagen, Valencia, CA, USA). One microgram of total RNA was reverse transcribed using the M-MLV Reverse Transcriptase (Invitrogen, San Diego, CA, USA). Three overlapping shorter amplicons [amplicon 1 (491 bp) exons 1–5; amplicon 2 (482 bp) exons 5–8; amplicon 3 (498 bp) exons 8–11)] covering the entire TP53 coding sequence [GenBank:NM_000546.4] were amplified with 2 U of FastStart Taq DNA Polymerase (Roche Diagnostics, Mannheim, Germany), 0.8 mM dNTPs, 1 mM MgCl2, and 0.2 M forward and reverse primers (Additional file 5: Table S3) in 25 μl reaction volumes. PCR products were purified using QIAquick PCR Purification Kit (Qiagen) and then directly sequenced using an ABI PRISM 3730 automated DNA sequencer (Applied Biosystems, Foster City, CA, USA) and a Big Dye Terminator DNA sequencing kit (Applied Biosystems). All sequence variations were detected by comparison using the BLAST software tool (www.ncbi.nlm.nih.gov/BLAST/) to reference genome sequence data [GenBank:NM_000546.4].

### Immunohistochemistry

Studies evaluating Chk1, phosphorylated Chk1 (Ser345), Chk2, phosphorylated Chk2 (Thr68) Cdc25C, phosphorylated Cdc25C (Ser216), and phosphorylated H2A.X (Ser139) (γ-H2A.X) protein expressions were performed on FFPE samples corresponding to three thymuses, three reactive lymph nodes, and to primary tumors collected from 60 ALL patients at diagnosis, including 36 B-ALL (median age 50 years, range 5–86; ratio male to female 18/18) and 24 T-ALL (median age 38 years, range 2–76; ratio male to female 15/9). All the cases were retrieved from the archives of the Haematopathology Unit, Department of Experimental, Diagnostic and Specialty Medicine—DIMES, University of Bologna. The study was conducted according to the principles of the Declaration of Helsinki after approval of the Internal Review Board. Two different tissue microarrays (TMAs) were constructed from these paraffin-embedded blocks as previously reported. TMA sections were investigated by antibodies raised against fixation resistant epitopes. The antibody reactivity and sources as well as the antigen retrieval protocols, dilutions, and revelation systems are detailed in Additional file 5: Table S4. A cutoff of staining >30 % of the examined cells was assigned as positive score, according to formerly defined criteria [29]. Immunohistochemical preparations were visualized, and images were captured using Olympus dotSlide microscope digital system equipped with the VS110 image analysis software.

### Primary cells

Primary blast cells from 14 newly diagnosed ALL cases were obtained, upon written informed consent, from bone marrow and peripheral blood samples by density gradient centrifugation over Lymphoprep (Nycomed UK, Birmingham, UK). ten (71 %) samples were from adults with BCR-ABL1-positive ALL (median age 51 years, range 25–74 years; median blast percentage 92 %, range 60–100 %) and four (29 %) from patients with BCR-ABL1-negative ALL (median age 34 years, range 18–43 years; median blast percentage 93 %, range 90–100 %). Main patients’ characteristics are given in Additional file 5: Table S1.

Normal bone marrow progenitors were harvested from bone marrow aspirations performed in lymphoma patients undergoing initial staging procedures, which then resulted negative for lymphoma infiltration. Normal peripheral blood progenitors were harvested from healthy donors.

### Cell viability assay

In order to assess the cell viability after treatment with PF-00477736 (Pfizer), ALL cell lines were seeded in 96-well plates at 50,000 cell/100 μl/well with increasing concentrations of drug (0.005–2 μM) for 24 and 48 h and incubated at 37 °C. Cell viability was assessed by adding WST-1 reagent (Roche Applied Science, Basel, Switzerland) to the culture medium at 1:10 dilution. Cells were incubated at 37 °C, and the optical density was measured by microplate ELISA reader at *λ* = 450 after 3 h. The amount of the formazan formed directly correlates to the number of metabolically active cells. All viability experiments were performed in triplicates and repeated in least two separated experiments. In ex-vivo primary leukemia cells, the effects on cell viability were assessed by counting viable and non-viable cell numbers by the trypan blue dye exclusion method. Cells were seeded in six-well plates at 500,000 cell/1 ml with increasing concentrations of drug (0.1, 0.5, and 1 μM) for 24 h and incubated at 37 °C. Cellular viability was calculated as a percentage of the viable cells compared to the untreated controls (DMSO 0.1 %).

### Annexin V staining of apoptotic cells

According to the WST-1 results, three different increasing concentrations of PF-00477736 were used to treat leukemia cells lines in order to detect and discriminate apoptotic, necrotic and dead cells. Cell lines were seeded in 12-well plates at 500,000 cell/1 ml with increasing concentrations of drug (0.1, 0.5 and 1 μM and 0.05, 0.1 and 0.2 μM only for BV-173 and RPMI-8402) for 24 and 48 h and incubated at 37 °C. Following the treatment, cells were harvested and stained with Annexin V/PI according to the manufacturer’s instruction (Roche). The percentage of Annexin V-PI-positive cells was determined within 1 × 10^4^ cells of the population by flow cytometry (FACSCanto II, BD Biosciences Pharmingen, San Jose, CA, USA). The mean percentage of Annexin V-PI-positive cells and standard error measurement was calculated from at least two separate experiments.

### Western blot analysis

To gain insight into the molecular mechanisms responsible for cell death following treatment, functional analyses by Western blot were performed. Leukemia cell lines were plated in six-well plates at 500,000 cell/1 ml with increasing concentrations of drug for 24 h and incubated at 37 °C. After the treatment, the cells were collected and lysate using a specific buffer made of KH_2_PO_4_ 0.1 M (pH 7.5), Igepal 1 % (NP-40), β-glicerofosfato 0.1 mM, and complete protease inhibitor cocktail 1× (Roche Diagnostics). For each sample, 30 μg of protein was fractioned on Mini-PROTEAN TGX stain-free precasted gels, blotted to nitrocellulose membranes (Bio-Rad Trans-blot turbo transfer pack), and incubated overnight with the following antibodies: ATM (#2873S), phosphorylated ATM (Ser1981)(#5883S), ATR (#2790S), phosphorylated ATR (Ser428)(#2853S), Chk1 (#2345S), phosphorylated Chk1 (Ser317)(#2344S), phosphorylated Chk1 (Ser296)(#2349) and Chk1 (Ser345)(#2348S), Chk2 (#2662S), phosphorylated Chk2 (Thr68)(#2661S), Cdc25c (#4688S), phosphorylated Cdc25C (Ser216)(#9528S), PARP-1 (#9542), Cdc2 (#9112S), phosphorylated Cdc2 (Tyr15)(#4539S), and phosphorylated H2A.X (Ser139) (γ-H2A.X) (#2577S) from Cell Signaling. Antibody to β-actin came from Sigma (St. Louis, MO, USA). Finally, all these antibodies were detected using the enhanced chemiluminescence kit ECL (GE) and the compact darkroom ChemiDoc-It (UVP).

### Immunofluorescence analysis

NALM-6 and BV-173 cells were seeded to poly-d lysine-coated slides, fixed with 4 % PFA (paraformaldehyde), and stained at 37 °C with a mouse anti-H2A.X-Phosphorylated Alexa 647 conjugated antibody (BioLegend). Then, they were treated with DAPI (4.6 diamidino-2-phenylindole; Sigma Aldrich), and the slides were mounted with Mowiol (Calbiochem). Images were acquired with wide field fluorescence microscope Olympus BX61 fully motorized driven by Metamorph software, and the analysis was performed using the freeware ImageJ software.

### Gene expression profiling

Gene expression profiling on treated and untreated cells (DMSO 0.1 %) after 24 h of exposure to PF-0077736 was performed using Affymetrix GeneChip Human Gene 1.0 ST platform (Affymetrix Inc. Santa Clara, CA, USA) and following manufacturers’ instructions. Raw data were normalized by using the RMA algorithm and filtered. Genes differentially expressed were selected by analysis of variance (ANOVA) (*p* value threshold =0.05) using the Partek Genomics Suite software (Partek Incorporated Saint Louis, MO, USA; http://www.partek.com). The most significantly involved process networks were defined by the Metacore software (GeneGo Inc., www.genego.com).

### Cell cycle analysis

In order to evaluate the effect of PF-00477736 on cell cycle progression, NALM-6, SUPB-15, RPMI-8402, and BV-173 cell lines were seeded in a 24-well plate at 500,000 cell/1 ml with increasing concentrations of PF-00477736 (0.1, 0.5, and 1 μM for NALM-6 and SUP-B15; 0.05, 0.1, and 0.2 μM for RPMI-8402 and BV-173). After 6 and 24 h, cells were harvested and fixed overnight using ethanol at 70 %. Then, cells were stained using PI/RNase Staining Buffer according to the manufacturer’s instruction (BD Pharmingen), and the cell cycle profile was detected using FACSCanto II instrument. The percentage of the different cell cycle phases was performed using the DNA cell-cycle analysis software for flow cytometry data, ModFit LT (Verity).

### In vivo studies

Experiments involving mice were performed in agreement with Italian guidelines and after the approval of the Institutional Review Board of the European Institute of Oncology. Leukemic mice were generated by a single administration of the tumorigenic agent n-ethyl-n-nitrosourea (Sigma, 50 mg/kg intraperitoneally). Spleen cells from leukemic C57BL6/Ly5.2 mice were injected intravenously (2 × 10^6^ cells/mouse) into non-irradiated, recipient C57BL6/Ly5.1 mice. PF-0077736 was administered intraperitoneally starting from day 3 after the transplant of leukemic cells. Mice received doses of PF-0077736 (40 mg/kg each dose) every 3 days for four treatments (q3dx4). Kaplan-Meyer rank test was used to compare the survival rate between treated and control mice.

## Additional files

Additional file 1: Figure S1. Basal expression of Chk1 and Chk2 (mRNA level) in the cell lines treated, the results are expressed as 2exp(-ΔΔCt) (A). Schematic representation of the statistically significance of the ANOVA multiple comparison test in which the basal expression of each cell lines is compared to the value of the basal expression of all the other cell lines (B). **Figure S2.** Oncomine expression analysis of Chk1 (A) and Chk2 (B) levels between normal monunuclear cells (MNCs) from bone marrow and different subtypes of leukemia (AML, B-ALL, T-ALL). **Figure S3.** Apoptosis following treatment with PF-00477736 at 24 and 48 hours in B-ALL and T-ALL cells(A). Cell cycle analysis of RPMI-8402, BV-173, SUP-B15 and NALM-6 cell lines treated for 24 hours with increasing concentrations of PF-00477736 (B). **Figure S4.** Western Blot analysis in all leukemia cell lines after exposure to PF-00477736 (+) at the concentration closest the IC50 or DMSO 0.1 %(-) (A). Western Blot analysis of BV-173 and NALM-6 cell lines treated with or without PF-00477736 (IC50) for 3, 6 and 9 hours (B). **Figure S5.** Cell cycle_ESR1 regulation of G1/S transition: the top scored map (map with the lowest *p*-value) based on the enrichment distribution sorted by 'Statistically significant Maps' set. **Figure S6.** Apoptosis and survival_Granzyme A signaling: the second scored map (map with the second lowest *p*-value) based on the enrichment distribution sorted by 'Statistically significant Maps' set. **Figure S7.** DNA damage_ATM/ATR regulation of G1/S checkpoint: the third scored map (map with the third lowest *p*-value) based on the enrichment distribution sorted by 'Statistically significant Maps' set. **Figure S8.** Protein expression level of c-Jun in B-/T-ALL cell lines (A). Western Blot analysis of normal MNCs and ALL MNCs after exposure to PF-0047773(B). mRNA expression of CDK4, Chk2, GADD45a and PLK3 in B-/T-ALL cell lines treated with or without PF-00477736(IC50 value) for 24 hours. The results are expressed as 2exp(-ΔΔCt)(C). (PPTX 2905 kb) Additional file 2:
**Treatment resulted in a differential expression of 941 genes (** ***p*** **< 0.05).** (PDF 256 kb) Additional file 3:
**Enrichment analysis report: enrichment by GO processes.** (PDF 41 kb) Additional file 4:
**Enrichment analysis report: enrichment by process networks.** (PDF 35 kb) Additional file 5:
**Table S1.** List of differentially expressed genes between treated cells and their untreated counterpart with a false discovery rate less than 0.05. **Table S2.** Main clinical and molecular characteristics of ALL patients whose primary ALL samples have been tested in vitro. **Table S3.** TP53 primer sequences. **Table S4.** Antibody reactivity and sources as well as the antigen retrieval protocols, dilutions, and revelation systems used in immunohistochemistry. (DOCX 28 kb)
