# Supplementary material for: In vitro and in vivo single-agent efficacy of checkpoint kinase inhibition in acute lymphoblastic leukemia
Source: J Hematol Oncol. 2015 Nov 5;8:125. doi: 10.1186/s13045-015-0206-5 (PMC4635624; doi:10.1186/s13045-015-0206-5)
Supplement: Additional file 1: Figure S1. — Basal expression of Chk1 and Chk2 (mRNA level) in the cell lines treated, the results are expressed as 2exp(-ΔΔCt) (A). Schematic representation of the statistically significance of the ANOVA multiple comparison test in which the basal expression of each cell lines is compared to the value of the basal expression of all the other cell lines (B). Figure S2. Oncomine expression analysis of Chk1 (A) and Chk2 (B) levels between normal monunuclear cells (MNCs) from bone marrow and different subtypes of leukemia (AML, B-ALL, T-ALL). Figure S3. Apoptosis following treatment with PF-00477736 at 24 and 48 hours in B-ALL and T-ALL cells(A). Cell cycle analysis of RPMI-8402, BV-173, SUP-B15 and NALM-6 cell lines treated for 24 hours with increasing concentrations of PF-00477736 (B). Figure S4. Western Blot analysis in all leukemia cell lines after exposure to PF-00477736 (+) at the concentration closest the IC50 or DMSO 0.1 %(-) (A). Western Blot analysis of BV-173 and NALM-6 cell lines treated with or without PF-00477736 (IC50) for 3, 6 and 9 hours (B). Figure S5. Cell cycle_ESR1 regulation of G1/S transition: the top scored map (map with the lowest p-value) based on the enrichment distribution sorted by 'Statistically significant Maps' set. Figure S6. Apoptosis and survival_Granzyme A signaling: the second scored map (map with the second lowest p-value) based on the enrichment distribution sorted by 'Statistically significant Maps' set. Figure S7. DNA damage_ATM/ATR regulation of G1/S checkpoint: the third scored map (map with the third lowest p-value) based on the enrichment distribution sorted by 'Statistically significant Maps' set. Figure S8. Protein expression level of c-Jun in B-/T-ALL cell lines (A). Western Blot analysis of normal MNCs and ALL MNCs after exposure to PF-0047773(B). mRNA expression of CDK4, Chk2, GADD45a and PLK3 in B-/T-ALL cell lines treated with or without PF-00477736(IC50 value) for 24 hours. The results are expressed as 2exp(-ΔΔCt)(C). (PPTX 2905 [file 13045_2015_206_MOESM1_ESM.pptx]

## Slide 1
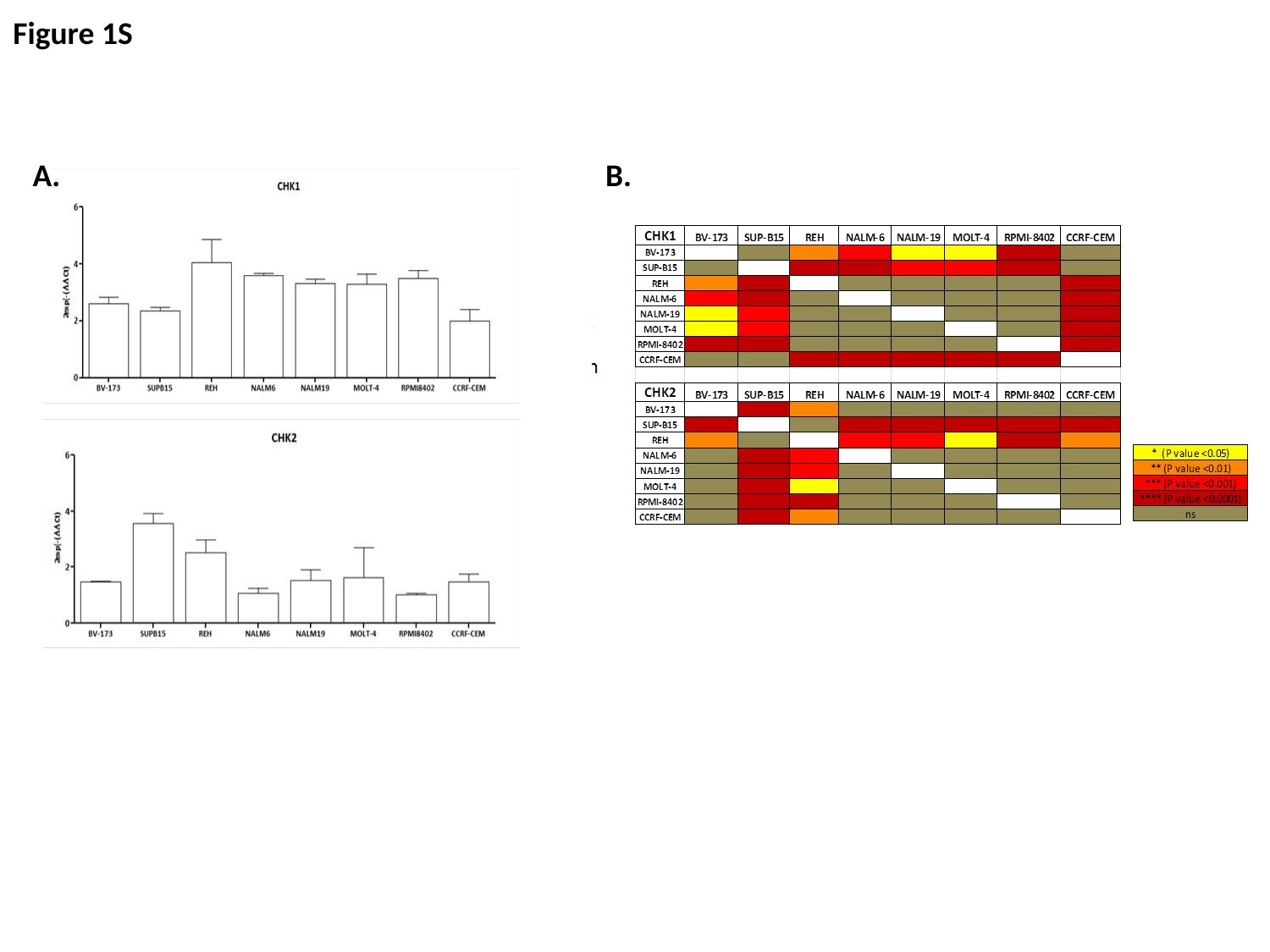

Figure 1S
A.
B.

## Slide 2
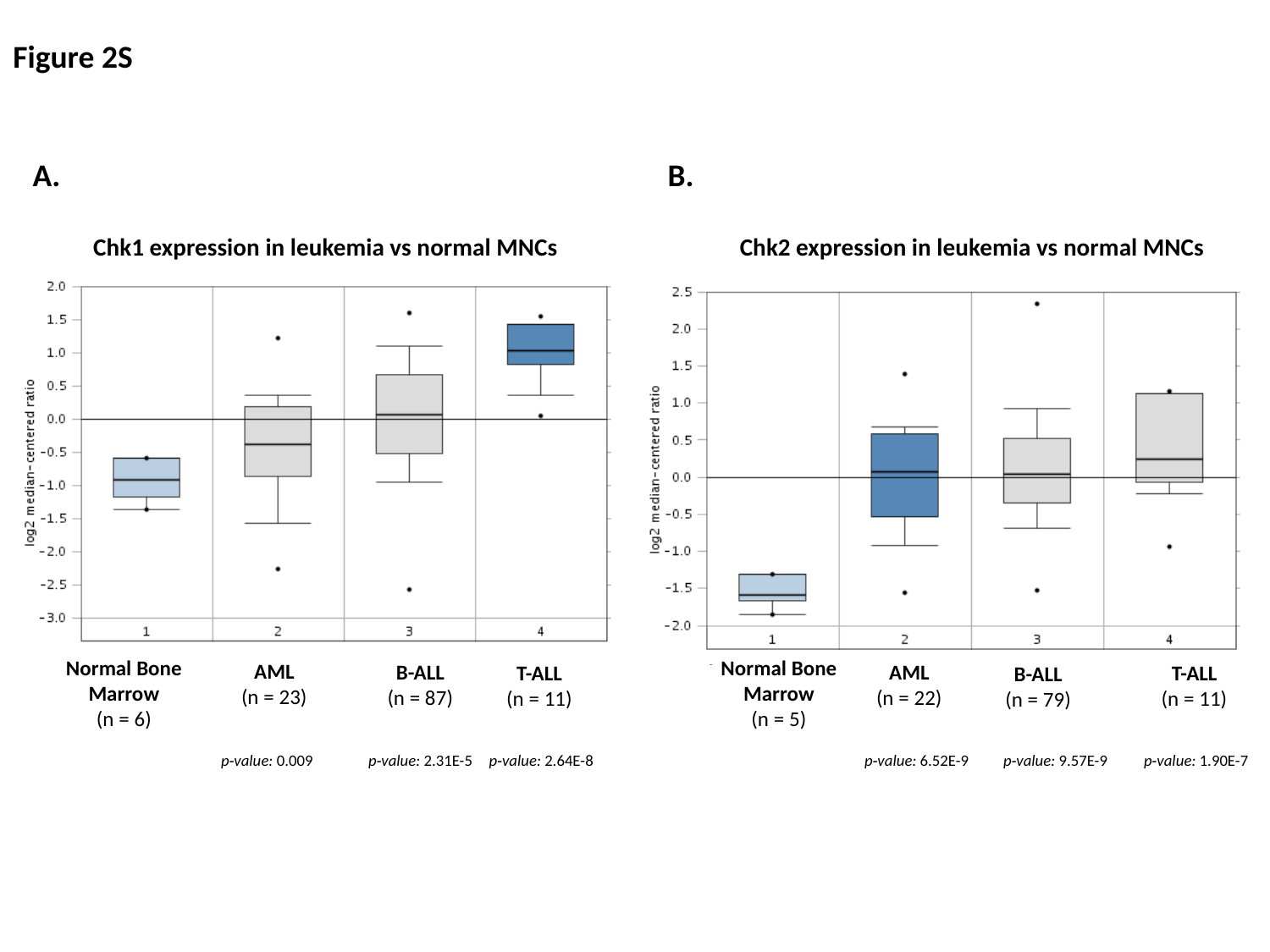

Figure 2S
A.
B.
Chk1 expression in leukemia vs normal MNCs
Chk2 expression in leukemia vs normal MNCs
Normal Bone Marrow
(n = 6)
Normal Bone Marrow
(n = 5)
AML
(n = 23)
B-ALL
(n = 87)
AML
(n = 22)
T-ALL
(n = 11)
T-ALL
(n = 11)
B-ALL
(n = 79)
p‑value: 0.009
p‑value: 2.31E-5
p‑value: 2.64E-8
p‑value: 6.52E-9
p‑value: 9.57E-9
p‑value: 1.90E-7

## Slide 3
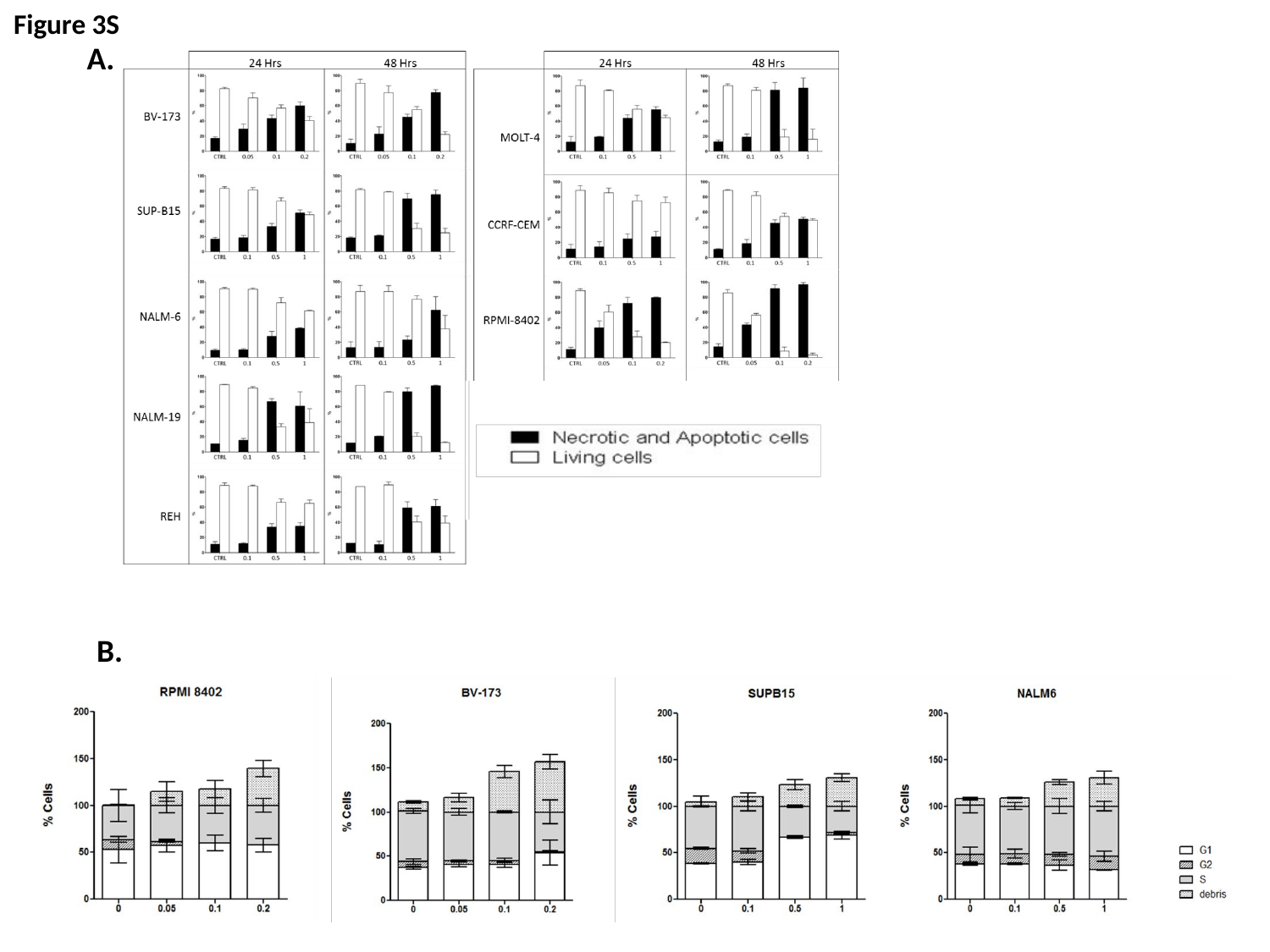

Figure 3S
A.
B.

## Slide 4
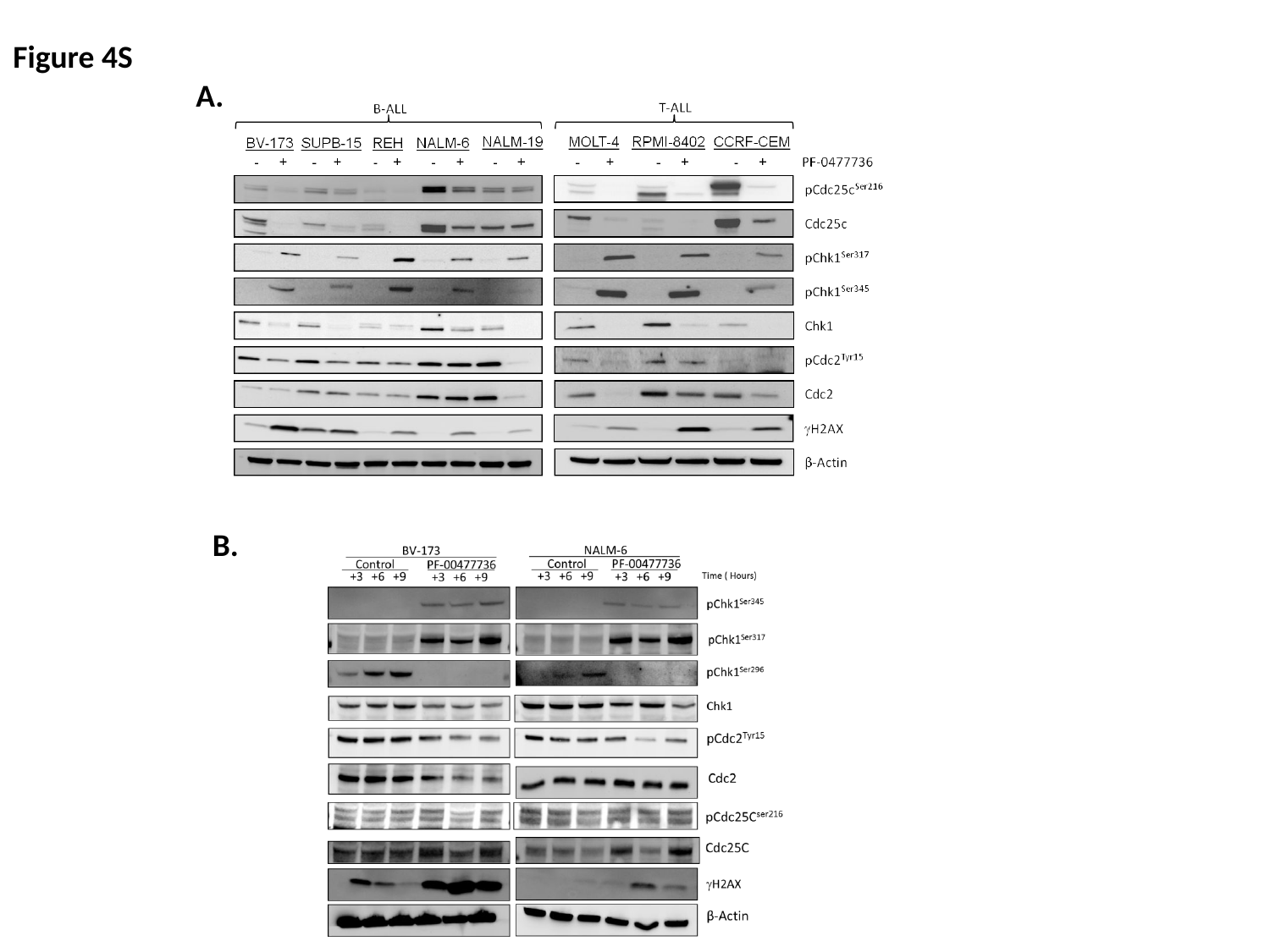

Figure 4S
A.
B.

## Slide 5
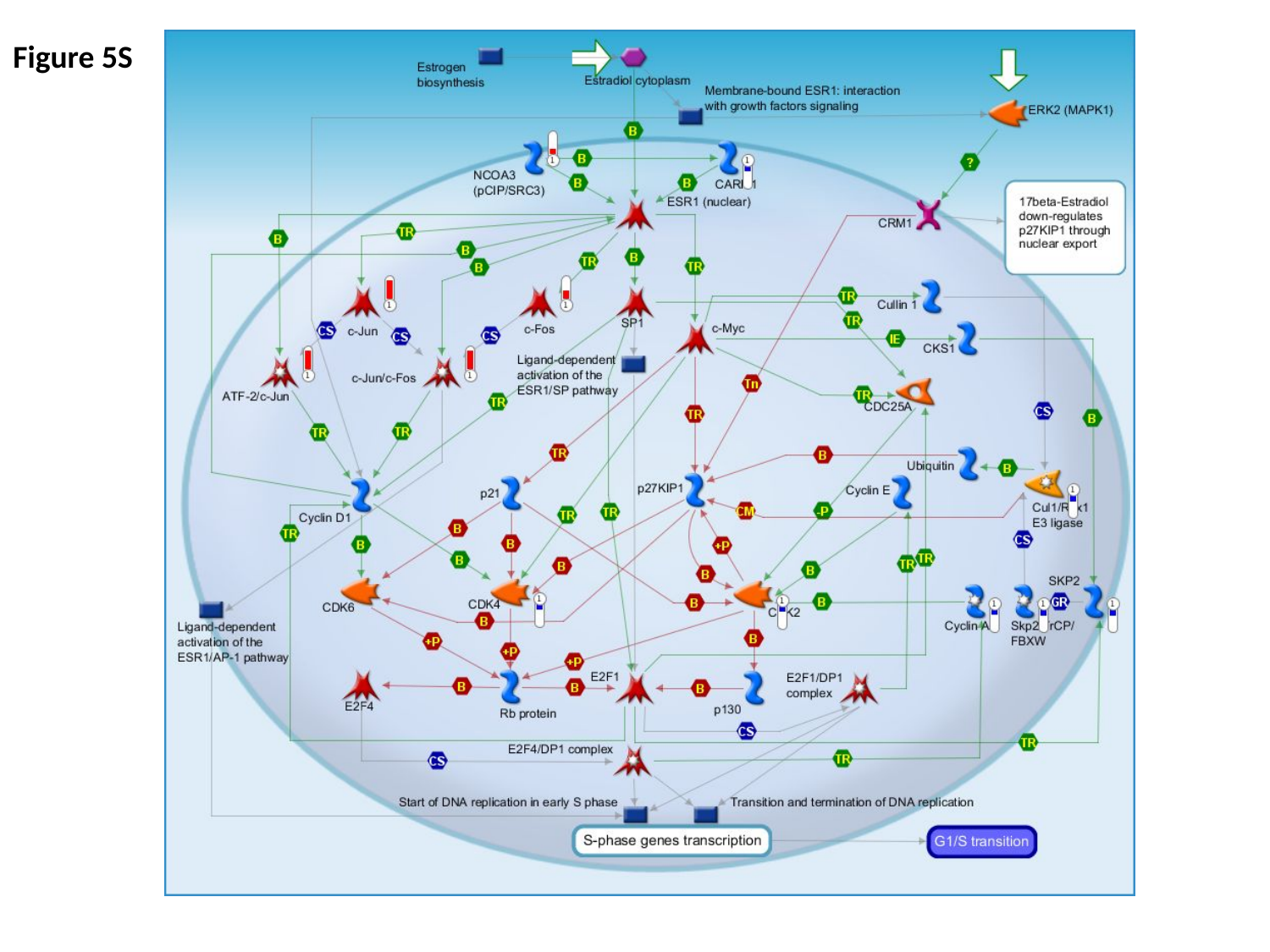

Figure 5S

## Slide 6
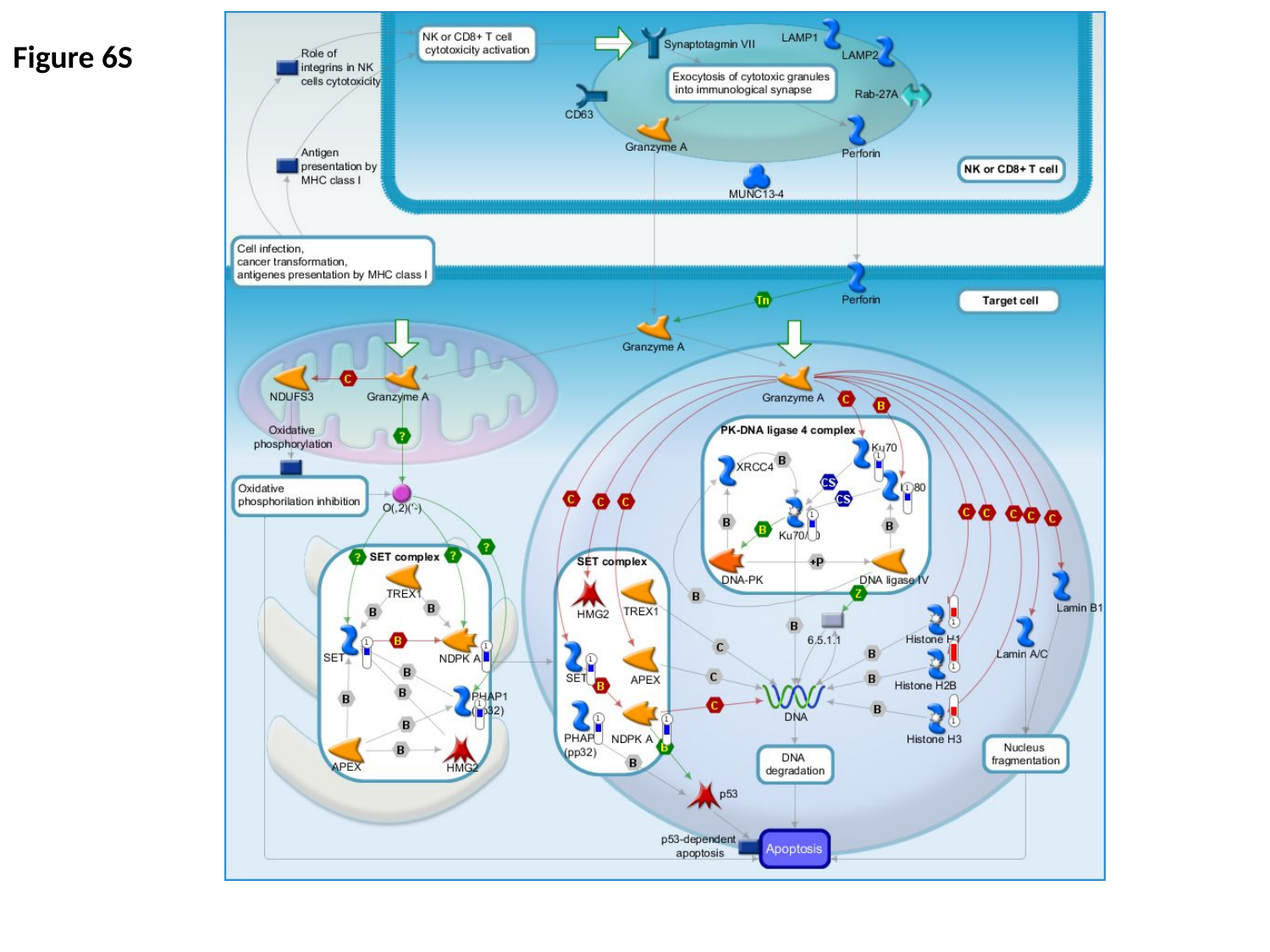

Figure 6S

## Slide 7
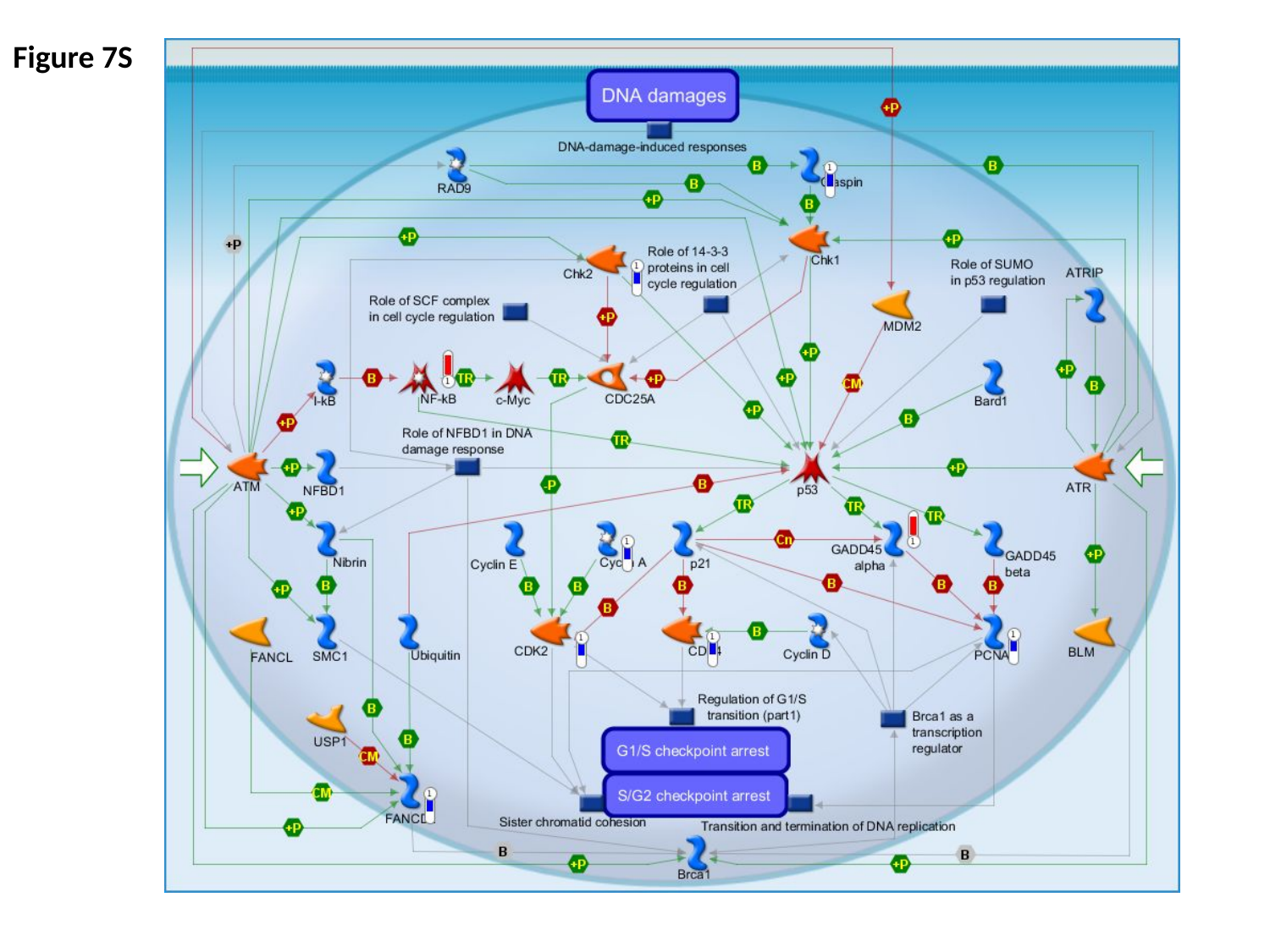

Figure 7S

## Slide 8
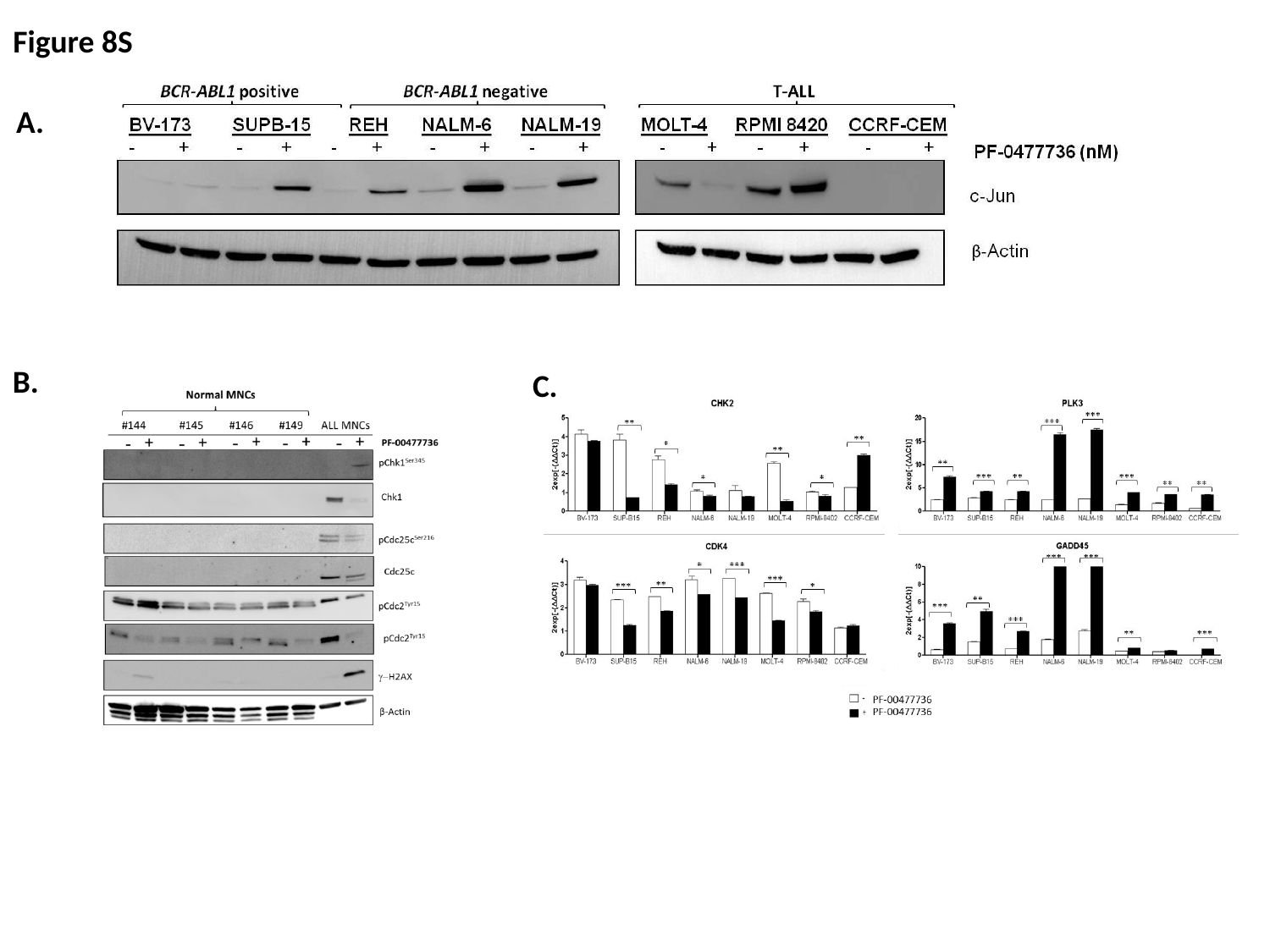

Figure 8S
A.
B.
C.
