# Supplementary material for: In vitro and in vivo single-agent efficacy of checkpoint kinase inhibition in acute lymphoblastic leukemia
Source: J Hematol Oncol. 2015 Nov 5;8:125. doi: 10.1186/s13045-015-0206-5 (PMC4635624; doi:10.1186/s13045-015-0206-5)
Supplement: Additional file 2: — Treatment resulted in a differential expression of 941 genes ( p < 0.05). (PDF 256 kb) [file 13045_2015_206_MOESM2_ESM.pdf]

Column #	Transcript ID	Gene Symbol	RefSeq	p-value(Treatment)	Mean(DMSO)	LSMean(DMSO)	p-value(PF-00477736 vs. DMSO)	Ratio(PF-00477736 vs. DMSO)	Fold-Change(PF-00477736 vs. DMSO)	Fold-Change(PF-00477736 vs. DMSO) (Description)
20364	8058914	AAMP	NM_001087	0.00233078	8.37162	8.37162	0.00233078	0.823485	-1.21435	PF-00477736 down vs DMSO
14759	8002347	AARS	NM_001605	0.0466349	9.86692	9.86692	0.0466349	1.40831	1.40831	PF-00477736 up vs DMSO
11773	7972297	ABCC4	NM_005845	0.036395	8.1223	8.1223	0.036395	0.64068	-1.56084	PF-00477736 down vs DMSO
24215	8097647	ABCE1	NM_002940	0.0237173	9.70655	9.70655	0.0237173	0.704324	-1.4198	PF-00477736 down vs DMSO
15920	8014487	ACACA	NM_198839	0.00465182	8.78227	8.78227	0.00465182	0.656759	-1.52263	PF-00477736 down vs DMSO
23478	8090511	ACAD9	NR_033426	0.0334495	4.86311	4.86311	0.0334495	1.2094	1.2094	PF-00477736 up vs DMSO
26917	8123137	ACAT2	NM_005891	0.0155366	9.23601	9.23601	0.0155366	0.677593	-1.47581	PF-00477736 down vs DMSO
14723	8002057	ACD	NM_001082486	0.0372476	7.71141	7.71141	0.0372476	0.80328	-1.2449	PF-00477736 down vs DMSO
16019	8015460	ACLY	NM_001096	0.0175606	10.5622	10.5622	0.0175606	0.764924	-1.30732	PF-00477736 down vs DMSO
24472	8099965	ACOT7	NM_007274	0.0178785	8.49802	8.49802	0.0178785	0.661511	-1.51169	PF-00477736 down vs DMSO
5732	7912012	ACOT7	NM_007274	0.0379129	8.17551	8.17551	0.0379129	0.851131	-1.17491	PF-00477736 down vs DMSO
19422	8048733	ACSL3	NM_004457	0.00194206	9.23703	9.23703	0.00194206	0.785791	-1.2726	PF-00477736 down vs DMSO
13933	7993726	ACSM5	NM_017888	0.0195938	4.65477	4.65477	0.0195938	0.925836	-1.0801	PF-00477736 down vs DMSO
7987	7934906	ACTA2	NM_001141945	0.0414549	5.75747	5.75747	0.0414549	1.89891	1.89891	PF-00477736 up vs DMSO
13360	7987315	ACTC1	NM_005159	0.0464735	4.78004	4.78004	0.0464735	0.861385	-1.16092	PF-00477736 down vs DMSO
6683	7921821	ADAMTS4	NM_005099	0.0263687	4.82976	4.82976	0.0263687	0.876789	-1.14053	PF-00477736 down vs DMSO
21822	8073242	ADSL	NM_000026	0.0391221	9.41942	9.41942	0.0391221	0.778343	-1.28478	PF-00477736 down vs DMSO
29125	8144378	AGPAT5	NM_018361	0.000635376	8.95975	8.95975	0.000635376	0.713056	-1.40241	PF-00477736 down vs DMSO
15382	8008664	AKAP1	NM_003488	0.0316319	9.22481	9.22481	0.0316319	0.745579	-1.34124	PF-00477736 down vs DMSO
17961	8035023	AKAP8L	NM_014371	0.0184159	8.89065	8.89065	0.0184159	1.24189	1.24189	PF-00477736 up vs DMSO
27486	8128788	AKD1	NM_001145128	0.0484291	4.19406	4.19406	0.0484291	1.51639	1.51639	PF-00477736 up vs DMSO
8021	7935230	ALDH18A1	NM_002860	0.0210157	9.04198	9.04198	0.0210157	0.713658	-1.40123	PF-00477736 down vs DMSO
14018	7994737	ALDOA	NM_000034	0.00224825	9.66786	9.66786	0.00224825	0.749457	-1.3343	PF-00477736 down vs DMSO
8151	7936923	ALDOAP2	M21191	0.0104962	5.97013	5.97013	0.0104962	0.780565	-1.28112	PF-00477736 down vs DMSO
15838	8013660	ALDOC	NM_005165	0.0281531	8.41021	8.41021	0.0281531	0.457838	-2.18418	PF-00477736 down vs DMSO
15031	8005399	ALKBH5	NM_017758	0.0299715	9.27321	9.27321	0.0299715	0.864384	-1.15689	PF-00477736 down vs DMSO
11249	7967149	ANAPC5	NM_016237	0.0139596	9.59904	9.59904	0.0139596	0.770764	-1.29741	PF-00477736 down vs DMSO
12899	7982531	ANP32A	NM_006305	0.0135808	11.2537	11.2537	0.0135808	0.835866	-1.19636	PF-00477736 down vs DMSO
13565	7990020	ANP32A	NM_006305	0.0156837	10.131	10.131	0.0156837	0.772569	-1.29438	PF-00477736 down vs DMSO
30518	8156750	ANP32B	NM_006401	0.0117012	10.7219	10.7219	0.0117012	0.736292	-1.35816	PF-00477736 down vs DMSO
17515	8030470	AP2A1	NM_014203	0.0376961	8.62376	8.62376	0.0376961	0.802267	-1.24647	PF-00477736 down vs DMSO
15170	8006542	AP2B1	NM_001030006	0.0339995	10.2089	10.2089	0.0339995	0.878742	-1.13799	PF-00477736 down vs DMSO
17762	8032670	APBA3	NM_004886	0.0465276	6.27366	6.27366	0.0465276	1.1715	1.1715	PF-00477736 up vs DMSO
8429	7939424	API5	NM_001142930	0.0273292	10.3879	10.3879	0.0273292	0.779255	-1.28328	PF-00477736 down vs DMSO
18745	8042402	APLF	NM_173545	0.0495547	7.00444	7.00444	0.0495547	1.51442	1.51442	PF-00477736 up vs DMSO
6684	7921834	APOA2	NM_001643	0.0254175	5.73669	5.73669	0.0254175	0.900265	-1.11078	PF-00477736 down vs DMSO
16431	8019765	ARHGDIA	NM_001185077	0.0484706	10.3041	10.3041	0.0484706	0.785176	-1.2736	PF-00477736 down vs DMSO
28148	8135229	ARMC10	NM_031905	0.0185609	8.22978	8.22978	0.0185609	0.748872	-1.33534	PF-00477736 down vs DMSO
23341	8089034	ARMC10	NM_031905	0.0283346	8.16419	8.16419	0.0283346	0.654152	-1.5287	PF-00477736 down vs DMSO
28090	8134581	ARPC1A	NM_006409	0.0370789	9.40309	9.40309	0.0370789	0.870753	-1.14843	PF-00477736 down vs DMSO
6791	7922793	ARPC5	NM_005717	0.02246	9.92443	9.92443	0.02246	0.923761	-1.08253	PF-00477736 down vs DMSO
25845	8113073	ARRDC3	NM_020801	0.00155508	8.31082	8.31082	0.00155508	2.17704	2.17704	PF-00477736 up vs DMSO
15672	8012028	ASGR2	NM_080912	0.0488542	5.5018	5.5018	0.0488542	1.13382	1.13382	PF-00477736 up vs DMSO
16548	8020844	ASXL3	NM_030632	0.0246263	3.94243	3.94243	0.0246263	1.24491	1.24491	PF-00477736 up vs DMSO
4239	7896952	ATAD3A	NM_018188	0.0327664	8.76908	8.76908	0.0327664	0.809874	-1.23476	PF-00477736 down vs DMSO
4238	7896937	ATAD3C	NM_001039211	0.0118469	6.69343	6.69343	0.0118469	0.810968	-1.23309	PF-00477736 down vs DMSO
5479	7909610	ATF3	NM_001040619	0.000835378	5.49884	5.49884	0.000835378	2.76388	2.76388	PF-00477736 up vs DMSO
23390	8089527	ATG3	NM_022488	0.022949	9.73973	9.73973	0.022949	0.839209	-1.1916	PF-00477736 down vs DMSO
19368	8048120	ATIC	NM_004044	0.0487314	10.1829	10.1829	0.0487314	0.75121	-1.33119	PF-00477736 down vs DMSO
23721	8092849	ATP13A3	NM_024524	0.0339614	9.7351	9.7351	0.0339614	0.822868	-1.21526	PF-00477736 down vs DMSO
4949	7904254	ATP1A1	NM_000701	0.0225777	10.708	10.708	0.0225777	0.69714	-1.43443	PF-00477736 down vs DMSO
10454	7958644	ATP2A2	NM_170665	0.0363922	10.6874	10.6874	0.0363922	0.8019	-1.24704	PF-00477736 down vs DMSO
7123	7926084	ATP5C1	NM_001001973	0.0226502	10.8369	10.8369	0.0226502	0.893058	-1.11975	PF-00477736 down vs DMSO
15339	8008132	ATP5G1	NM_005175	0.00903721	12.0487	12.0487	0.00903721	0.764457	-1.30812	PF-00477736 down vs DMSO
10545	7959657	ATP6V0A2	NM_012463	0.0469813	8.89506	8.89506	0.0469813	0.844782	-1.18374	PF-00477736 down vs DMSO
28222	8136039	ATP6V1F	NM_004231	0.0188719	9.79298	9.79298	0.0188719	0.749276	-1.33462	PF-00477736 down vs DMSO
22530	8080878	ATXN7	NM_000333	0.032048	9.68539	9.68539	0.032048	1.26194	1.26194	PF-00477736 up vs DMSO
10994	7964555	AVIL	NM_006576	0.0223365	5.21788	5.21788	0.0223365	1.25356	1.25356	PF-00477736 up vs DMSO
18181	8037032	B3GNT8	NM_198540	0.0311319	5.72463	5.72463	0.0311319	1.13781	1.13781	PF-00477736 up vs DMSO
4621	7900931	B4GALT2	NM_003780	0.00436271	8.7788	8.7788	0.00436271	0.692361	-1.44433	PF-00477736 down vs DMSO
18179	8037018	B9D2	NM_030578	0.00729568	6.541	6.541	0.00729568	1.20091	1.20091	PF-00477736 up vs DMSO
18273	8037872	BBC3	NM_001127240	0.0326482	6.72157	6.72157	0.0326482	1.19971	1.19971	PF-00477736 up vs DMSO
7618	7931268	BCCIP	NM_016567	0.0487701	8.7524	8.7524	0.0487701	0.793446	-1.26033	PF-00477736 down vs DMSO
13472	7988852	BCL2L10	NM_020396	0.0112458	4.78959	4.78959	0.0112458	1.18507	1.18507	PF-00477736 up vs DMSO
12212	7976567	BDKRB1	NM_000710	0.0473434	4.46539	4.46539	0.0473434	0.885516	-1.12928	PF-00477736 down vs DMSO
8588	7940582	BEST1	NM_004183	0.00341003	5.04941	5.04941	0.00341003	2.3629	2.3629	PF-00477736 up vs DMSO
17076	8026035	BEST2	NM_017682	0.0431396	4.68513	4.68513	0.0431396	1.15133	1.15133	PF-00477736 up vs DMSO
22743	8082774	BFSP2	NM_003571	0.0434297	4.84714	4.84714	0.0434297	1.19087	1.19087	PF-00477736 up vs DMSO
27346	8127193	BMP5	NM_021073	0.0220969	3.30626	3.30626	0.0220969	0.903551	-1.10674	PF-00477736 down vs DMSO
8168	7937079	BNIP3	NM_004052	0.0356865	9.75025	9.75025	0.0356865	0.43987	-2.2734	PF-00477736 down vs DMSO
22646	8081686	BOC	NM_033254	0.0231607	4.44767	4.44767	0.0231607	1.13229	1.13229	PF-00477736 up vs DMSO
10552	7959777	BRI3BP	NM_080626	0.0379022	10.2561	10.2561	0.0379022	0.70661	-1.41521	PF-00477736 down vs DMSO
16884	8023955	BSG	NM_001728	0.0434207	10.1527	10.1527	0.0434207	0.875232	-1.14255	PF-00477736 down vs DMSO
24594	8101002	BTC	NM_001729	0.0207555	4.0909	4.0909	0.0207555	1.13601	1.13601	PF-00477736 up vs DMSO
11088	7965423	BTG1	NM_001731	0.000915303	9.29435	9.29435	0.000915303	1.91036	1.91036	PF-00477736 up vs DMSO
5420	7908917	BTG2	NM_006763	0.0473404	7.73753	7.73753	0.0473404	2.0536	2.0536	PF-00477736 up vs DMSO
26328	8117485	BTN2A1	NM_078476	0.0159617	6.95074	6.95074	0.0159617	1.3262	1.3262	PF-00477736 up vs DMSO
8130	7936706	C10orf119	NM_024834	0.0245351	9.93015	9.93015	0.0245351	0.728016	-1.3736	PF-00477736 down vs DMSO
8159	7936996	C10orf90	NM_001004298	0.0275295	4.08732	4.08732	0.0275295	0.860531	-1.16207	PF-00477736 down vs DMSO
8839	7943162	C11orf54	NM_014039	0.0315265	8.24193	8.24193	0.0315265	1.30513	1.30513	PF-00477736 up vs DMSO
9514	7949327	C11orf85	BC106951	0.00939632	4.05475	4.05475	0.00939632	1.14611	1.14611	PF-00477736 up vs DMSO
9764	7951593	C11orf92	NR_034154	0.0460335	4.68546	4.68546	0.0460335	1.13731	1.13731	PF-00477736 up vs DMSO
9931	7953211	C12orf5	NM_020375	0.0339502	6.39555	6.39555	0.0339502	1.57274	1.57274	PF-00477736 up vs DMSO
10338	7957351	C12orf64	NM_173591	0.0428505	2.83803	2.83803	0.0428505	1.13774	1.13774	PF-00477736 up vs DMSO
11826	7972826	C13orf29	NR_027701	0.0485803	3.65612	3.65612	0.0485803	1.13331	1.13331	PF-00477736 up vs DMSO
11804	7972665	C13orf39	BC132748	0.0355037	3.47891	3.47891	0.0355037	1.08788	1.08788	PF-00477736 up vs DMSO
12413	7978527	C14orf126	NM_080664	0.0112167	8.76385	8.76385	0.0112167	0.734365	-1.36172	PF-00477736 down vs DMSO
13242	7986239	C15orf32	BC109252	0.0342561	4.03324	4.03324	0.0342561	0.905352	-1.10454	PF-00477736 down vs DMSO
12962	7983298	C15orf63	NM_016400	0.0212448	7.61226	7.61226	0.0212448	0.86329	-1.15836	PF-00477736 down vs DMSO
14366	7998449	C16orf42	NM_001001410	0.0405338	7.11137	7.11137	0.0405338	0.860874	-1.16161	PF-00477736 down vs DMSO
13819	7992614	C16orf59	NM_025108	0.0154303	7.24879	7.24879	0.0154303	0.885819	-1.1289	PF-00477736 down vs DMSO
13884	7993159	C16orf72	NM_014117	0.0469191	8.55628	8.55628	0.0469191	1.15806	1.15806	PF-00477736 up vs DMSO
15876	8014073	C17orf79	NM_018405	0.00910888	7.77799	7.77799	0.00910888	0.754416	-1.32553	PF-00477736 down vs DMSO
16706	8022404	C18orf19	NM_001098801	0.021335	7.67472	7.67472	0.021335	0.722053	-1.38494	PF-00477736 down vs DMSO
17227	8027416	C19orf2	NM_003796	0.0108182	10.1741	10.1741	0.0108182	0.779056	-1.2836	PF-00477736 down vs DMSO
5026	7904974	C1orf152	NR_003242	0.0420649	11.722	11.722	0.0420649	0.833315	-1.20003	PF-00477736 down vs DMSO
5544	7910261	C1orf69	NM_001010867	0.0443192	6.29508	6.29508	0.0443192	1.29923	1.29923	PF-00477736 up vs DMSO
6163	7916629	C1orf87	NM_152377	0.039166	3.7682	3.7682	0.039166	1.08229	1.08229	PF-00477736 up vs DMSO
15658	8011850	C1QBP	NM_001212	0.0244621	10.6128	10.6128	0.0244621	0.739163	-1.35288	PF-00477736 down vs DMSO
9968	7953603	C1S	NM_201442	0.0265415	4.30423	4.30423	0.0265415	1.36091	1.36091	PF-00477736 up vs DMSO
21106	8066402	C20orf111	NM_016470	0.0150824	7.18171	7.18171	0.0150824	1.31367	1.31367	PF-00477736 up vs DMSO
20916	8064739	C20orf27	NM_001039140	0.035957	7.31414	7.31414	0.035957	0.736555	-1.35767	PF-00477736 down vs DMSO
21480	8069933	C21orf45	NM_018944	0.0407664	9.06492	9.06492	0.0407664	0.687177	-1.45523	PF-00477736 down vs DMSO
19793	8052834	C2orf42	BC005079	0.0332586	7.19259	7.19259	0.0332586	1.25887	1.25887	PF-00477736 up vs DMSO
19597	8050689	C2orf44	NM_025203	0.0354565	8.89301	8.89301	0.0354565	0.815914	-1.22562	PF-00477736 down vs DMSO
18860	8043407	C2orf51	NM_152670	0.0100887	4.82351	4.82351	0.0100887	1.21042	1.21042	PF-00477736 up vs DMSO
20273	8057954	C2orf66	AY358249	0.0387035	3.69958	3.69958	0.0387035	1.41945	1.41945	PF-00477736 up vs DMSO
22295	8078033	C3orf19	NM_016474	0.0248209	7.06595	7.06595	0.0248209	1.53928	1.53928	PF-00477736 up vs DMSO
26449	8118409	C4A	NM_007293	0.0273243	5.25296	5.25296	0.0273243	1.15805	1.15805	PF-00477736 up vs DMSO
26450	8118455	C4A	NM_007293	0.0273243	5.25296	5.25296	0.0273243	1.15805	1.15805	PF-00477736 up vs DMSO
33005	8179399	C4A	NM_007293	0.0273243	5.25296	5.25296	0.0273243	1.15805	1.15805	PF-00477736 up vs DMSO
24029	8095767	C4orf26	BC117342	0.00677176	3.63946	3.63946	0.00677176	1.10619	1.10619	PF-00477736 up vs DMSO
25215	8106999	C5orf27	NR_026936	0.00335604	4.50268	4.50268	0.00335604	1.22598	1.22598	PF-00477736 up vs DMSO
25520	8110032	C5orf41	NM_153607	0.0138226	6.42945	6.42945	0.0138226	1.79987	1.79987	PF-00477736 up vs DMSO
25523	8110068	C5orf47	NM_001144954	0.0198482	3.46955	3.46955	0.0198482	1.07399	1.07399	PF-00477736 up vs DMSO
25468	8109572	C5orf52	NM_001145132	0.0470827	3.86676	3.86676	0.0470827	1.09749	1.09749	PF-00477736 up vs DMSO
27233	8126095	C6orf129	NM_138493	0.0196705	7.96895	7.96895	0.0196705	0.792196	-1.26231	PF-00477736 down vs DMSO
27267	8126382	C6orf132	NM_001164446	0.0237456	5.73499	5.73499	0.0237456	1.08054	1.08054	PF-00477736 up vs DMSO
32867	8178090	C6orf48	NM_001040437	0.0332682	9.81981	9.81981	0.0332682	1.62362	1.62362	PF-00477736 up vs DMSO
33000	8179326	C6orf48	NM_001040437	0.0332682	9.81981	9.81981	0.0332682	1.62362	1.62362	PF-00477736 up vs DMSO
28506	8138647	C7orf31	NM_138811	0.0419089	7.03729	7.03729	0.0419089	1.26646	1.26646	PF-00477736 up vs DMSO
28211	8135915	C7orf68	NM_013332	0.0474169	8.03213	8.03213	0.0474169	0.701707	-1.4251	PF-00477736 down vs DMSO
30683	8158539	C9orf106	NM_001012715	0.0472199	5.67659	5.67659	0.0472199	1.11706	1.11706	PF-00477736 up vs DMSO
30531	8156897	C9orf30	BC008993	0.00778103	8.61335	8.61335	0.00778103	0.754974	-1.32455	PF-00477736 down vs DMSO
30758	8159415	C9orf86	NM_024718	0.0430517	8.60648	8.60648	0.0430517	0.870106	-1.14929	PF-00477736 down vs DMSO
18610	8040843	CAD	NM_004341	0.0156864	8.89317	8.89317	0.0156864	0.702111	-1.42428	PF-00477736 down vs DMSO
17449	8029831	CALM3	NM_005184	0.0116517	10.3046	10.3046	0.0116517	0.805382	-1.24165	PF-00477736 down vs DMSO
7943	7934477	CAMK2G	NM_172171	0.00304502	8.92108	8.92108	0.00304502	0.857466	-1.16623	PF-00477736 down vs DMSO
8647	7941179	CAPN1	NM_005186	0.0336981	8.70829	8.70829	0.0336981	0.833532	-1.19971	PF-00477736 down vs DMSO
17044	8025766	CARM1	NM_199141	0.0225797	9.26608	9.26608	0.0225797	0.783856	-1.27575	PF-00477736 down vs DMSO
27792	8131949	CBX3	NM_016587	0.0414834	9.2688	9.2688	0.0414834	0.870958	-1.14816	PF-00477736 down vs DMSO
16340	8019018	CBX4	NM_003655	0.000767904	8.37853	8.37853	0.000767904	1.76116	1.76116	PF-00477736 up vs DMSO
17087	8026170	CCDC130	NM_030818	0.00649358	8.07785	8.07785	0.00649358	1.34998	1.34998	PF-00477736 up vs DMSO
15556	8010629	CCDC137	NM_199287	0.0196974	7.68108	7.68108	0.0196974	0.827264	-1.2088	PF-00477736 down vs DMSO
26840	8122327	CCDC28A	NM_015439	0.0499929	6.59551	6.59551	0.0499929	1.2251	1.2251	PF-00477736 up vs DMSO
4597	7900555	CCDC30	NM_001080850	0.0163675	4.1151	4.1151	0.0163675	1.48071	1.48071	PF-00477736 up vs DMSO
23432	8089988	CCDC58	NM_001017928	0.0295488	8.71376	8.71376	0.0295488	0.743289	-1.34537	PF-00477736 down vs DMSO
17456	8029894	CCDC9	NM_015603	0.0150158	6.65122	6.65122	0.0150158	1.15582	1.15582	PF-00477736 up vs DMSO

27005	8124008	CCDC90A	NM_001031713	0.0445061	8.59	8.59	0.0445061	0.790193	-1.26551	PF-00477736 down vs DMSO
15179	8006608	CCL4L1	NM_001001435	0.00201532	4.10213	4.10213	0.00201532	1.22592	1.22592	PF-00477736 up vs DMSO
15180	8006621	CCL4L1	NM_001001435	0.00729162	4.27813	4.27813	0.00729162	1.25788	1.25788	PF-00477736 up vs DMSO
16414	8019651	CCL4L1	NM_001001435	0.00729162	4.27813	4.27813	0.00729162	1.25788	1.25788	PF-00477736 up vs DMSO
24750	8102643	CCNA2	NM_001237	0.0266383	10.3479	10.3479	0.0266383	0.713243	-1.40205	PF-00477736 down vs DMSO
24040	8095870	CCNG2	NM_004354	0.00495453	7.42329	7.42329	0.00495453	1.82447	1.82447	PF-00477736 up vs DMSO
23593	8091658	CCNL1	NM_020307	0.000566784	8.7314	8.7314	0.000566784	1.64698	1.64698	PF-00477736 up vs DMSO
19101	8045381	CCNT2	NM_058241	0.0242315	8.61383	8.61383	0.0242315	1.25133	1.25133	PF-00477736 up vs DMSO
19757	8052562	CCT4	NM_006430	0.00366289	10.3033	10.3033	0.00366289	0.666096	-1.50128	PF-00477736 down vs DMSO
24966	8104449	CCT5	NM_012073	0.00538542	10.9554	10.9554	0.00538542	0.712589	-1.40333	PF-00477736 down vs DMSO
27900	8132943	CCT6A	NM_001762	0.0302095	10.0295	10.0295	0.0302095	0.745382	-1.34159	PF-00477736 down vs DMSO
17435	8029688	CD3EAP	NM_012099	0.0393091	7.01076	7.01076	0.0393091	0.762257	-1.31189	PF-00477736 down vs DMSO
10678	7961075	CD69	NM_001781	0.00169334	6.42376	6.42376	0.00169334	3.16956	3.16956	PF-00477736 up vs DMSO
25716	8112053	CDC20B	NM_152623	0.000645219	4.08815	4.08815	0.000645219	1.26152	1.26152	PF-00477736 up vs DMSO
21613	8071212	CDC45	NM_001178010	0.04615	9.42691	9.42691	0.04615	0.634949	-1.57493	PF-00477736 down vs DMSO
19204	8046488	CDCA7	NM_031942	0.0445873	9.15336	9.15336	0.0445873	0.754847	-1.32477	PF-00477736 down vs DMSO
10230	7956076	CDK2	NM_001798	0.036415	9.58721	9.58721	0.036415	0.726673	-1.37613	PF-00477736 down vs DMSO
10991	7964522	CDK4	NM_000075	0.029788	10.9537	10.9537	0.029788	0.743493	-1.345	PF-00477736 down vs DMSO
29077	8143850	CDK5	NM_004935	0.0338712	8.27637	8.27637	0.0338712	0.656298	-1.5237	PF-00477736 down vs DMSO
24317	8098500	CDKN2AIP	NM_017632	0.049098	7.99978	7.99978	0.049098	1.38597	1.38597	PF-00477736 up vs DMSO
17871	8034075	CDKN2D	NM_001800	0.023961	6.90404	6.90404	0.023961	1.22126	1.22126	PF-00477736 up vs DMSO
20787	8063386	CEBPB	NM_005194	0.00120031	7.71196	7.71196	0.00120031	2.11244	2.11244	PF-00477736 up vs DMSO
17242	8027566	CEBPG	NM_001806	0.0383159	8.01499	8.01499	0.0383159	1.44392	1.44392	PF-00477736 up vs DMSO
20918	8064762	CENPB	NM_001810	0.0472772	7.19438	7.19438	0.0472772	0.806817	-1.23944	PF-00477736 down vs DMSO
25109	8105842	CENPH	NM_022909	0.00181882	7.63116	7.63116	0.00181882	0.642396	-1.55667	PF-00477736 down vs DMSO
30478	8156341	CENPP	NM_001012267	0.0446794	8.02713	8.02713	0.0446794	0.749607	-1.33403	PF-00477736 down vs DMSO
30667	8158250	CERCAM	NM_016174	0.0329844	6.57449	6.57449	0.0329844	0.740939	-1.34964	PF-00477736 down vs DMSO
33002	8179351	CFB	NM_001710	0.0412379	5.3873	5.3873	0.0412379	0.88272	-1.13286	PF-00477736 down vs DMSO
9531	7949496	CFL1	NM_005507	0.0398584	11.9571	11.9571	0.0398584	0.801707	-1.24734	PF-00477736 down vs DMSO
18309	8038288	CGB1	NM_033377	0.0494712	5.68214	5.68214	0.0494712	1.09671	1.09671	PF-00477736 up vs DMSO
12929	7982868	CHAC1	NM_024111	0.0486471	7.53308	7.53308	0.0486471	2.10011	2.10011	PF-00477736 up vs DMSO
21334	8068478	CHAF1B	NM_005441	0.0349435	8.88197	8.88197	0.0349435	0.699145	-1.43032	PF-00477736 down vs DMSO
21998	8074939	CHCHD10	NM_213720	0.0362752	8.83012	8.83012	0.0362752	0.726325	-1.37679	PF-00477736 down vs DMSO
23029	8085481	CHCHD4	NM_144636	0.0082342	6.31061	6.31061	0.0082342	0.751327	-1.33098	PF-00477736 down vs DMSO
22028	8075164	CHEK2	NM_001005735	0.0278447	6.85039	6.85039	0.0278447	0.743321	-1.34531	PF-00477736 down vs DMSO
24518	8100382	CHIC2	NM_012110	0.0306179	9.00693	9.00693	0.0306179	1.29527	1.29527	PF-00477736 up vs DMSO
9080	7945579	CHID1	NM_001142674	0.0220219	7.9661	7.9661	0.0220219	0.773656	-1.29256	PF-00477736 down vs DMSO
22224	8077123	CHKB-CPT1B	NR_027928	0.0384536	7.43108	7.43108	0.0384536	1.28337	1.28337	PF-00477736 up vs DMSO
17969	8035110	CIB3	NM_054113	0.0319345	4.5756	4.5756	0.0319345	1.11151	1.11151	PF-00477736 up vs DMSO
4801	7902721	CLCA1	NM_001285	0.0437	3.07867	3.07867	0.0437	0.952599	-1.04976	PF-00477736 down vs DMSO
10680	7961091	CLEC2A	NM_001130711	0.0210509	3.15237	3.15237	0.0210509	1.11782	1.11782	PF-00477736 up vs DMSO
17004	8025303	CLEC4M	NR_026707	0.00136035	4.41121	4.41121	0.00136035	0.881053	-1.13501	PF-00477736 down vs DMSO
21323	8068383	CLIC6	NM_053277	0.00840592	3.82318	3.82318	0.00840592	1.13651	1.13651	PF-00477736 up vs DMSO
20285	8058127	CLK1	NR_027856	0.0200307	8.38574	8.38574	0.0200307	1.64318	1.64318	PF-00477736 up vs DMSO
25599	8110803	CLPTM1L	NM_030782	0.0273549	9.67328	9.67328	0.0273549	0.773751	-1.29241	PF-00477736 down vs DMSO
5989	7914851	CLSPN	NM_022111	0.0408708	7.93717	7.93717	0.0408708	0.682213	-1.46582	PF-00477736 down vs DMSO
23312	8088866	CNTN3	NM_020872	0.0128599	3.67008	3.67008	0.0128599	0.856675	-1.1673	PF-00477736 down vs DMSO
14971	8004741	CNTROB	NM_001037144	0.0201842	7.42439	7.42439	0.0201842	1.30821	1.30821	PF-00477736 up vs DMSO
13599	7990436	COX5A	NM_004255	0.00192346	10.6663	10.6663	0.00192346	0.796003	-1.25628	PF-00477736 down vs DMSO
18913	8043718	COX5B	NM_001862	0.0156192	10.3827	10.3827	0.0156192	0.710011	-1.40843	PF-00477736 down vs DMSO
26513	8119153	COX6A1	NM_004373	0.0285419	11.2744	11.2744	0.0285419	0.874198	-1.14391	PF-00477736 down vs DMSO
10504	7959153	COX6A1	NM_004373	0.0339407	11.628	11.628	0.0339407	0.873729	-1.14452	PF-00477736 down vs DMSO
17278	8027932	COX6B1	NM_001863	0.0264302	10.0144	10.0144	0.0264302	0.793833	-1.25971	PF-00477736 down vs DMSO
28245	8136217	CPA5	NM_001127441	0.0387182	4.03439	4.03439	0.0387182	1.09832	1.09832	PF-00477736 up vs DMSO
31753	8168638	CPXCR1	NM_033048	0.0429409	3.34297	3.34297	0.0429409	1.13926	1.13926	PF-00477736 up vs DMSO
19344	8047839	CREB1	NM_004379	0.0385602	9.91516	9.91516	0.0385602	1.20324	1.20324	PF-00477736 up vs DMSO
20332	8058524	CRYGC	NM_020989	0.0437557	5.27804	5.27804	0.0437557	1.08282	1.08282	PF-00477736 up vs DMSO
10960	7964064	CS	NM_004077	0.00918954	10.55	10.55	0.00918954	0.818422	-1.22186	PF-00477736 down vs DMSO
20776	8063283	CSE1L	NM_001316	0.0117323	10.8281	10.8281	0.0117323	0.689617	-1.45008	PF-00477736 down vs DMSO
7228	7927146	CSGALNACT2	NM_018590	0.0246343	7.55457	7.55457	0.0246343	1.37305	1.37305	PF-00477736 up vs DMSO
23150	8086799	CSPG5	NM_006574	0.0171171	4.98032	4.98032	0.0171171	0.888997	-1.12486	PF-00477736 down vs DMSO
11977	7974090	CTAGE5	NM_005930	0.0302277	6.09293	6.09293	0.0302277	1.16657	1.16657	PF-00477736 up vs DMSO
4592	7900510	CTPS	NM_001905	0.0489636	9.86796	9.86796	0.0489636	0.774446	-1.29124	PF-00477736 down vs DMSO
7785	7933047	CUL2	NM_003591	0.0191242	8.624	8.624	0.0191242	0.819168	-1.22075	PF-00477736 down vs DMSO
27200	8125752	CUTA	NM_015921	0.0349932	9.37622	9.37622	0.0349932	0.810525	-1.23377	PF-00477736 down vs DMSO
30185	8153819	CYHR1	NM_138496	0.0399647	6.82786	6.82786	0.0399647	1.47627	1.47627	PF-00477736 up vs DMSO
11810	7972699	DAOA	NM_001161812	0.0126497	3.98779	3.98779	0.0126497	0.85404	-1.1709	PF-00477736 down vs DMSO
10596	7960320	DCP1B	NM_152640	0.0214224	7.94281	7.94281	0.0214224	1.52663	1.52663	PF-00477736 up vs DMSO
14597	8000884	DCTPP1	NM_024096	0.006708	10.8157	10.8157	0.006708	0.696416	-1.43592	PF-00477736 down vs DMSO
9442	7948534	DDB1	NM_001923	0.0358602	10.0151	10.0151	0.0358602	0.864086	-1.15729	PF-00477736 down vs DMSO
10987	7964460	DDIT3	NM_001195053	6.06E-05	6.97175	6.97175	6.06E-05	3.32494	3.32494	PF-00477736 up vs DMSO
25329	8108134	DDX46	NM_014829	0.00187593	9.37102	9.37102	0.00187593	0.809769	-1.23492	PF-00477736 down vs DMSO
11201	7966570	DDX54	NM_001111322	0.0497549	8.88389	8.88389	0.0497549	0.812695	-1.23047	PF-00477736 down vs DMSO
11122	7965760	DEPDC4	NM_152317	0.0373089	4.41121	4.41121	0.0373089	0.704859	-1.41872	PF-00477736 down vs DMSO
9596	7950067	DHCR7	NM_001360	0.00439928	9.38179	9.38179	0.00439928	0.586244	-1.70577	PF-00477736 down vs DMSO
16739	8022640	DHFR	NM_000791	0.0101205	10.1791	10.1791	0.0101205	0.736837	-1.35715	PF-00477736 down vs DMSO
25819	8112902	DHFR	NM_000791	0.0124174	9.99694	9.99694	0.0124174	0.744915	-1.34244	PF-00477736 down vs DMSO
14240	7997179	DHODH	NM_001361	0.00600728	6.91561	6.91561	0.00600728	0.760959	-1.31413	PF-00477736 down vs DMSO
15849	8013804	DHRS13	NM_144683	0.0401491	7.26139	7.26139	0.0401491	0.847082	-1.18052	PF-00477736 down vs DMSO
24438	8099649	DHX15	NM_001358	0.00265058	10.1406	10.1406	0.00265058	0.827898	-1.20788	PF-00477736 down vs DMSO
15659	8011861	DHX33	NM_020162	0.0352407	9.08126	9.08126	0.0352407	0.772377	-1.2947	PF-00477736 down vs DMSO
17459	8029918	DHX34	NM_014681	0.00922351	7.20981	7.20981	0.00922351	0.814766	-1.22735	PF-00477736 down vs DMSO
31080	8162254	DIRAS2	NM_017594	0.0355159	5.40738	5.40738	0.0355159	0.894357	-1.11812	PF-00477736 down vs DMSO
32032	8170971	DKC1	NM_001363	0.0198284	9.72877	9.72877	0.0198284	0.673962	-1.48376	PF-00477736 down vs DMSO
11429	7969171	DLEU1	NR_002605	0.0483094	7.83442	7.83442	0.0483094	0.668877	-1.49504	PF-00477736 down vs DMSO
7892	7934026	DNA2	NM_001080449	0.00282152	8.29224	8.29224	0.00282152	0.658829	-1.51784	PF-00477736 down vs DMSO
22496	8080226	DNAH1	NM_015512	0.00431908	6.39122	6.39122	0.00431908	1.53861	1.53861	PF-00477736 up vs DMSO
14522	8000034	DNAH3	NM_017539	0.0140983	3.9101	3.9101	0.0140983	1.13638	1.13638	PF-00477736 up vs DMSO
20267	8057821	DNAH7	NM_018897	0.0397325	3.81686	3.81686	0.0397325	1.09052	1.09052	PF-00477736 up vs DMSO
19403	8048523	DNAJB2	NM_006736	0.0426681	6.01504	6.01504	0.0426681	1.32698	1.32698	PF-00477736 up vs DMSO
4619	7900911	DPH2	NM_001384	0.0454134	7.87018	7.87018	0.0454134	0.719719	-1.38943	PF-00477736 down vs DMSO
31276	8164336	DPM2	NM_003863	0.0289069	7.18112	7.18112	0.0289069	0.789698	-1.26631	PF-00477736 down vs DMSO
9084	7945641	DUSP8	NM_004420	0.0181096	6.20423	6.20423	0.0181096	1.21143	1.21143	PF-00477736 up vs DMSO
12988	7983594	DUT	NM_001025248	0.0203259	9.17875	9.17875	0.0203259	0.776522	-1.28779	PF-00477736 down vs DMSO
22632	8081503	DZIP3	NM_014648	0.0344338	6.22725	6.22725	0.0344338	1.65096	1.65096	PF-00477736 up vs DMSO
22302	8078138	EAF1	NM_033083	0.0429427	8.61493	8.61493	0.0429427	1.22972	1.22972	PF-00477736 up vs DMSO
31587	8167305	EBP	NM_006579	0.0234475	8.8398	8.8398	0.0234475	0.69448	-1.43993	PF-00477736 down vs DMSO
25829	8112980	EDIL3	NM_005711	0.0169535	3.29536	3.29536	0.0169535	1.08466	1.08466	PF-00477736 up vs DMSO
5142	7905918	EFNA3	NM_004952	0.0124017	6.34815	6.34815	0.0124017	0.72376	-1.38167	PF-00477736 down vs DMSO
16077	8016099	EFTUD2	NM_004247	0.0286337	10.3718	10.3718	0.0286337	0.809323	-1.2356	PF-00477736 down vs DMSO
25350	8108370	**EGR1**	NM_001964	0.0316728	7.68272	7.68272	0.0316728	2.57069	2.57069	PF-00477736 up vs DMSO
7880	7933872	EGR2	NM_000399	0.0223665	5.42903	5.42903	0.0223665	1.29465	1.29465	PF-00477736 up vs DMSO
9020	7944978	EI24	NM_004879	0.0231005	10.4019	10.4019	0.0231005	0.783656	-1.27607	PF-00477736 down vs DMSO
32116	8171747	EIF1AX	NM_001412	0.0389455	9.85792	9.85792	0.0389455	0.780835	-1.28068	PF-00477736 down vs DMSO
5826	7912852	EIF1AX	NM_001412	0.0433455	10.4638	10.4638	0.0433455	0.762971	-1.31067	PF-00477736 down vs DMSO
6075	7915640	EIF2B3	NM_020365	0.0432515	8.15169	8.15169	0.0432515	0.743794	-1.34446	PF-00477736 down vs DMSO
22931	8084694	EIF4A2	NM_001967	0.0435603	10.0037	10.0037	0.0435603	1.17201	1.17201	PF-00477736 up vs DMSO
19461	8049180	EIF4E2	NM_004846	0.0141725	8.30766	8.30766	0.0141725	0.846543	-1.18128	PF-00477736 down vs DMSO
7329	7928119	EIF4EBP2	NM_004096	0.0234154	10.82	10.82	0.0234154	0.838253	-1.19296	PF-00477736 down vs DMSO
32185	8172345	ELK1	NM_001114123	0.00223534	8.27788	8.27788	0.00223534	0.837009	-1.19473	PF-00477736 down vs DMSO
21097	8066294	EMILIN3	NM_052846	0.0192735	4.77267	4.77267	0.0192735	1.16349	1.16349	PF-00477736 up vs DMSO
9457	7948718	EML3	NM_153265	0.00785894	7.60038	7.60038	0.00785894	0.784875	-1.27409	PF-00477736 down vs DMSO
5749	7912198	ENO1	NM_001428	0.0332794	12.5124	12.5124	0.0332794	0.677525	-1.47596	PF-00477736 down vs DMSO
12580	7980080	ENTPD5	NM_001249	0.0398382	6.4288	6.4288	0.0398382	0.711989	-1.40452	PF-00477736 down vs DMSO
32350	8173930	EPAG	L22650	0.0427699	3.96935	3.96935	0.0427699	1.20364	1.20364	PF-00477736 up vs DMSO
4500	7899534	EPB41	NM_001166005	0.0407862	9.43403	9.43403	0.0407862	0.796669	-1.25523	PF-00477736 down vs DMSO
7774	7932938	EPC1	NM_025209	0.00792227	8.58995	8.58995	0.00792227	1.46768	1.46768	PF-00477736 up vs DMSO
29162	8144516	ERI1	NM_153332	0.0213179	7.9491	7.9491	0.0213179	0.723776	-1.38164	PF-00477736 down vs DMSO
16227	8017555	ERN1	NM_001433	0.000690183	7.28866	7.28866	0.000690183	1.59077	1.59077	PF-00477736 up vs DMSO
12479	7979179	ERO1L	NM_014584	0.0177762	9.35124	9.35124	0.0177762	0.68246	-1.46529	PF-00477736 down vs DMSO
7004	7925043	EXOC8	NM_175876	0.0498146	7.20277	7.20277	0.0498146	1.31859	1.31859	PF-00477736 up vs DMSO
6105	7915995	FAF1	NM_007051	0.0127435	9.46088	9.46088	0.0127435	0.770745	-1.29745	PF-00477736 down vs DMSO
15510	8010050	FAM100B	NM_182565	0.00525422	9.13676	9.13676	0.00525422	1.60065	1.60065	PF-00477736 up vs DMSO
21978	8074842	FAM108A1	NM_001130111	0.0199751	8.16708	8.16708	0.0199751	0.865425	-1.15546	PF-00477736 down vs DMSO
31913	8169995	FAM122C	NM_001170779	0.0495489	7.303	7.303	0.0495489	1.41512	1.41512	PF-00477736 up vs DMSO
26220	8116620	FAM136B	NM_001012983	0.0307635	6.39017	6.39017	0.0307635	0.868144	-1.15188	PF-00477736 down vs DMSO
29700	8149324	FAM167A	NM_053279	0.0197798	4.92997	4.92997	0.0197798	1.18214	1.18214	PF-00477736 up vs DMSO
23790	8093601	FAM193A	NM_003704	0.0434457	8.5732	8.5732	0.0434457	1.20922	1.20922	PF-00477736 up vs DMSO
31818	8169085	FAM199X	NM_207318	0.0344557	7.49822	7.49822	0.0344557	1.14581	1.14581	PF-00477736 up vs DMSO
5319	7907680	FAM20B	NM_014864	0.017035	8.33796	8.33796	0.017035	0.764229	-1.30851	PF-00477736 down vs DMSO
7242	7927267	FAM35B	NR_027632	0.0187388	8.35924	8.35924	0.0187388	0.780356	-1.28147	PF-00477736 down vs DMSO
7246	7927288	FAM35B2	NR_027634	0.0413902	8.74481	8.74481	0.0413902	0.83157	-1.20254	PF-00477736 down vs DMSO
25347	8108321	FAM53C	NM_001135647	0.0247521	7.95498	7.95498	0.0247521	1.50404	1.50404	PF-00477736 up vs DMSO
11312	7967879	FAM58A	NM_152274	0.032717	8.06676	8.06676	0.032717	0.82253	-1.21576	PF-00477736 down vs DMSO
9704	7951093	FAM76B	NM_144664	0.00397084	7.31916	7.31916	0.00397084	1.34302	1.34302	PF-00477736 up vs DMSO
13528	7989611	FAM96A	NM_032231	0.0105531	8.3567	8.3567	0.0105531	0.8158	-1.22579	PF-00477736 down vs DMSO
22267	8077731	FANCD2	NM_033084	0.0471749	9.14382	9.14382	0.0471749	0.733812	-1.36275	PF-00477736 down vs DMSO
20395	8059319	FARSB	NM_005687	0.0436397	10.659	10.659	0.0436397	0.729366	-1.37105	PF-00477736 down vs DMSO
16376	8019392	FASN	NM_004104	0.0122608	9.47264	9.47264	0.0122608	0.5935	-1.68492	PF-00477736 down vs DMSO

25612	8110920	FASTKD3	NM_024091	0.0127795	6.76742	6.76742	0.0127795	0.701131	-1.42627	PF-00477736 down vs DMSO
30941	8161229	FBXO10	NM_012166	0.00622892	8.9888	8.9888	0.00622892	0.858348	-1.16503	PF-00477736 down vs DMSO
22972	8084947	FBXO45	NM_001105573	0.0347105	8.05269	8.05269	0.0347105	0.71125	-1.40597	PF-00477736 down vs DMSO
19784	8052742	FBXO48	NM_001024680	0.0165999	5.1562	5.1562	0.0165999	1.5404	1.5404	PF-00477736 up vs DMSO
24889	8103755	FBXO8	NM_012180	0.0224551	7.13996	7.13996	0.0224551	1.33455	1.33455	PF-00477736 up vs DMSO
5150	7905986	FDPS	NM_002004	0.00800511	8.61885	8.61885	0.00800511	0.705446	-1.41754	PF-00477736 down vs DMSO
28739	8140420	FDPSL2A	NR_003262	0.00211256	6.72305	6.72305	0.00211256	0.780475	-1.28127	PF-00477736 down vs DMSO
28742	8140443	FDPSL2A	NR_003262	0.00311354	6.8963	6.8963	0.00311354	0.782936	-1.27724	PF-00477736 down vs DMSO
8585	7940561	FEN1	NM_004111	0.0126836	8.92887	8.92887	0.0126836	0.738109	-1.35481	PF-00477736 down vs DMSO
7047	7925480	FH	NM_000143	0.0466112	8.27836	8.27836	0.0466112	0.721546	-1.38591	PF-00477736 down vs DMSO
28619	8139632	FIGNL1	NM_001042762	0.00152777	7.75019	7.75019	0.00152777	0.705359	-1.41772	PF-00477736 down vs DMSO
5136	7905831	FLAD1	NM_025207	0.023109	6.89168	6.89168	0.023109	0.894979	-1.11734	PF-00477736 down vs DMSO
10084	7954692	FLJ13224	NR_026806	0.00333271	5.45113	5.45113	0.00333271	1.36268	1.36268	PF-00477736 up vs DMSO
14309	7997832	FLJ40448	ENST00000333666	0.0458412	5.85752	5.85752	0.0458412	1.12949	1.12949	PF-00477736 up vs DMSO
9346	7947969	FNBP4	NM_015308	0.0292954	9.79169	9.79169	0.0292954	1.25838	1.25838	PF-00477736 up vs DMSO
12132	7975779	FOS	NM_005252	0.000197284	6.0491	6.0491	0.000197284	2.40401	2.40401	PF-00477736 up vs DMSO
17436	8029693	FOSB	NM_006732	0.022821	6.063	6.063	0.022821	1.51044	1.51044	PF-00477736 up vs DMSO
30408	8155661	FOXD4L3	NM_199135	0.0299139	6.61952	6.61952	0.0299139	1.1654	1.1654	PF-00477736 up vs DMSO
31585	8167270	FTSJ1	NM_177439	0.0186387	8.51149	8.51149	0.0186387	0.7736	-1.29266	PF-00477736 down vs DMSO
6217	7917156	FUBP1	NM_003902	0.0407035	10.3607	10.3607	0.0407035	0.871693	-1.14719	PF-00477736 down vs DMSO
30412	8155699	FXN	NM_000144	0.00593876	9.17056	9.17056	0.00593876	0.716349	-1.39597	PF-00477736 down vs DMSO
25446	8109368	G3BP1	NM_005754	0.0085508	11.0232	11.0232	0.0085508	0.820936	-1.21812	PF-00477736 down vs DMSO
4751	7902227	GADD45A	NM_001924	0.0445687	6.61769	6.61769	0.0445687	2.16306	2.16306	PF-00477736 up vs DMSO
30473	8156309	GADD45G	NM_006705	0.0272798	5.16824	5.16824	0.0272798	1.2835	1.2835	PF-00477736 up vs DMSO
9947	7953385	GAPDH	NM_002046	0.0296038	13.2951	13.2951	0.0296038	0.886596	-1.12791	PF-00477736 down vs DMSO
21493	8070102	GART	NM_000819	0.0431011	9.08465	9.08465	0.0431011	0.76037	-1.31515	PF-00477736 down vs DMSO
17186	8027169	GATAD2A	NM_017660	0.0401982	9.83347	9.83347	0.0401982	0.783339	-1.27659	PF-00477736 down vs DMSO
18959	8044236	GCC2	NM_181453	0.0188366	8.14293	8.14293	0.0188366	1.43669	1.43669	PF-00477736 up vs DMSO
13017	7983890	GCOM1	NM_001018100	0.0425888	3.9922	3.9922	0.0425888	1.09222	1.09222	PF-00477736 up vs DMSO
26028	8114778	GCSH	NM_004483	0.00776778	9.47764	9.47764	0.00776778	0.657235	-1.52153	PF-00477736 down vs DMSO
14810	8002999	GCSH	NM_004483	0.0153004	8.60373	8.60373	0.0153004	0.660627	-1.51371	PF-00477736 down vs DMSO
6709	7922104	GCSH	NM_004483	0.0165417	8.78128	8.78128	0.0165417	0.659216	-1.51695	PF-00477736 down vs DMSO
26092	8115410	GEMIN5	NM_015465	0.038014	8.68686	8.68686	0.038014	0.778486	-1.28454	PF-00477736 down vs DMSO
28535	8138857	GGCT	NM_024051	0.0100993	8.11737	8.11737	0.0100993	0.750414	-1.3326	PF-00477736 down vs DMSO
14829	8003204	GINS2	NM_016095	0.021434	10.4235	10.4235	0.021434	0.670349	-1.49176	PF-00477736 down vs DMSO
24864	8103535	GK3P	NR_026575	0.0300273	8.74261	8.74261	0.0300273	0.772944	-1.29375	PF-00477736 down vs DMSO
14984	8004922	GLP2R	NM_004246	0.0191334	4.55119	4.55119	0.0191334	0.876671	-1.14068	PF-00477736 down vs DMSO
7627	7931393	GLRX3	NM_006541	0.00301499	8.62232	8.62232	0.00301499	0.823273	-1.21466	PF-00477736 down vs DMSO
12205	7976515	GLRX5	NM_016417	0.00921594	9.13837	9.13837	0.00921594	0.843375	-1.18571	PF-00477736 down vs DMSO
31886	8169709	GLRX5	NM_016417	0.0217093	9.72939	9.72939	0.0217093	0.782746	-1.27755	PF-00477736 down vs DMSO
17155	8026806	GLT25D1	NM_024656	0.00773513	9.12444	9.12444	0.00773513	0.740103	-1.35116	PF-00477736 down vs DMSO
11143	7965941	GLT8D2	NM_031302	0.0425774	4.1683	4.1683	0.0425774	1.11047	1.11047	PF-00477736 up vs DMSO
18755	8042487	GMCL1	NM_178439	0.0224123	9.02184	9.02184	0.0224123	0.865016	-1.15605	PF-00477736 down vs DMSO
22820	8083523	GMPS	NM_003875	0.0304505	10.9421	10.9421	0.0304505	0.716038	-1.39657	PF-00477736 down vs DMSO
13587	7990283	GOLGA6A	NM_001038640	0.0074624	5.55455	5.55455	0.0074624	1.14101	1.14101	PF-00477736 up vs DMSO
13120	7984961	GOLGA6B	NM_018652	0.00385851	5.53683	5.53683	0.00385851	1.17483	1.17483	PF-00477736 up vs DMSO
13096	7984662	GOLGA6B	NM_018652	0.00679099	5.47147	5.47147	0.00679099	1.12296	1.12296	PF-00477736 up vs DMSO
12857	7982230	GOLGA8IP	NR_024074	0.0187204	5.17564	5.17564	0.0187204	1.17382	1.17382	PF-00477736 up vs DMSO
13344	7987114	GOLGA8IP	NR_024074	0.023989	5.10663	5.10663	0.023989	1.1899	1.1899	PF-00477736 up vs DMSO
13327	7986947	GOLGA8IP	NR_024074	0.0331094	5.16453	5.16453	0.0331094	1.1528	1.1528	PF-00477736 up vs DMSO
6932	7924327	GPATCH2	NM_018040	0.0234231	7.37531	7.37531	0.0234231	1.31641	1.31641	PF-00477736 up vs DMSO
20932	8064868	GPCPD1	NM_019593	0.0382708	8.08202	8.08202	0.0382708	1.50841	1.50841	PF-00477736 up vs DMSO
14478	7999532	GSPT1	NM_002094	0.00838192	10.7484	10.7484	0.00838192	0.758435	-1.31851	PF-00477736 down vs DMSO
29784	8150103	GTF2E2	NM_002095	0.020219	8.24818	8.24818	0.020219	0.768251	-1.30166	PF-00477736 down vs DMSO
17799	8033135	GTF2F1	NM_002096	0.0458962	8.8509	8.8509	0.0458962	1.13283	1.13283	PF-00477736 up vs DMSO
28717	8140170	GTF2IRD2	NM_173537	0.0424131	6.81118	6.81118	0.0424131	1.88474	1.88474	PF-00477736 up vs DMSO
27996	8133549	GTF2IRD2B	NM_001003795	0.0342756	6.76498	6.76498	0.0342756	1.98757	1.98757	PF-00477736 up vs DMSO
17150	8026735	GTPBP3	NM_133644	0.0111448	7.75813	7.75813	0.0111448	0.770904	-1.29718	PF-00477736 down vs DMSO
16293	8018439	H3F3B	NM_005324	0.0126538	10.7359	10.7359	0.0126538	1.30168	1.30168	PF-00477736 up vs DMSO
10789	7962194	H3F3C	NM_001013699	0.0306208	10.1205	10.1205	0.0306208	1.2295	1.2295	PF-00477736 up vs DMSO
24887	8103745	HAND2	NM_021973	0.0245924	4.79882	4.79882	0.0245924	1.11669	1.11669	PF-00477736 up vs DMSO
19196	8046346	HAT1	NM_003642	0.0402546	9.76744	9.76744	0.0402546	0.748806	-1.33546	PF-00477736 down vs DMSO
24370	8098985	HAUS3	NM_024511	0.0473789	6.97286	6.97286	0.0473789	0.893585	-1.11909	PF-00477736 down vs DMSO
32595	8175782	HAUS7	NM_017518	0.00125199	7.10281	7.10281	0.00125199	0.825809	-1.21093	PF-00477736 down vs DMSO
32986	8179235	HCG27	NR_026791	0.0412472	6.2915	6.2915	0.0412472	1.22384	1.22384	PF-00477736 up vs DMSO
26024	8114691	HDAC3	NM_003883	0.0350281	10.2154	10.2154	0.0350281	0.850848	-1.1753	PF-00477736 down vs DMSO
14093	7995456	HEATR3	NM_182922	0.0487352	6.76621	6.76621	0.0487352	0.873307	-1.14507	PF-00477736 down vs DMSO
4869	7903401	HEJ1	NR_033424	0.0396715	3.91976	3.91976	0.0396715	0.895588	-1.11658	PF-00477736 down vs DMSO
14134	7995895	HERPUD1	NM_014685	0.0114699	8.82082	8.82082	0.0114699	1.74711	1.74711	PF-00477736 up vs DMSO
15297	8007745	HEXIM1	NM_006460	0.0160937	7.85174	7.85174	0.0160937	1.93099	1.93099	PF-00477736 up vs DMSO
30497	8156538	HIATL1	NM_032558	0.0182373	9.56437	9.56437	0.0182373	0.748478	-1.33604	PF-00477736 down vs DMSO
27052	8124397	HIST1H1C	NM_005319	0.0110902	9.55577	9.55577	0.0110902	1.38018	1.38018	PF-00477736 up vs DMSO
27058	8124430	HIST1H1D	NM_005320	0.0400157	9.13828	9.13828	0.0400157	1.40267	1.40267	PF-00477736 up vs DMSO
27050	8124391	HIST1H2AB	NM_003513	0.000272876	9.18365	9.18365	0.000272876	1.79793	1.79793	PF-00477736 up vs DMSO
26312	8117372	HIST1H2AC	NM_003512	0.00331293	9.9831	9.9831	0.00331293	1.38545	1.38545	PF-00477736 up vs DMSO
26336	8117535	HIST1H2AG	NM_021064	0.0123625	7.89372	7.89372	0.0123625	1.46975	1.46975	PF-00477736 up vs DMSO
26338	8117543	HIST1H2AH	NM_080596	0.00830262	8.00437	8.00437	0.00830262	1.39007	1.39007	PF-00477736 up vs DMSO
26348	8117583	HIST1H2AI	NM_003509	0.000408947	10.3069	10.3069	0.000408947	1.62348	1.62348	PF-00477736 up vs DMSO
26347	8117580	HIST1H2AI	NM_003509	0.00280351	10.2854	10.2854	0.00280351	1.53093	1.53093	PF-00477736 up vs DMSO
27077	8124518	HIST1H2AJ	NM_021066	0.000393236	6.03981	6.03981	0.000393236	5.14566	5.14566	PF-00477736 up vs DMSO
27079	8124524	HIST1H2AK	NM_003510	0.000106277	9.10732	9.10732	0.000106277	1.82333	1.82333	PF-00477736 up vs DMSO
26353	8117608	HIST1H2AL	NM_003511	0.00303833	9.15341	9.15341	0.00303833	1.50314	1.50314	PF-00477736 up vs DMSO
27084	8124540	HIST1H2AM	NM_003514	0.00168511	6.69658	6.69658	0.00168511	2.13115	2.13115	PF-00477736 up vs DMSO
27054	8124406	HIST1H2BC	NM_003526	0.0191889	8.87947	8.87947	0.0191889	1.36001	1.36001	PF-00477736 up vs DMSO
26314	8117382	HIST1H2BD	NM_021063	0.0362084	6.4851	6.4851	0.0362084	1.49275	1.49275	PF-00477736 up vs DMSO
26316	8117395	HIST1H2BF	NM_003522	0.0105254	10.4971	10.4971	0.0105254	1.42912	1.42912	PF-00477736 up vs DMSO
27070	8124484	HIST1H2BJ	NM_021058	0.00151919	6.52787	6.52787	0.00151919	2.65774	2.65774	PF-00477736 up vs DMSO
27076	8124510	HIST1H2BL	NM_003519	0.00301598	7.6843	7.6843	0.00301598	1.53604	1.53604	PF-00477736 up vs DMSO
26352	8117600	HIST1H2BN	NM_003520	0.0473288	6.93623	6.93623	0.0473288	1.22984	1.22984	PF-00477736 up vs DMSO
26354	8117614	HIST1H2BO	NM_003527	0.00486751	6.99995	6.99995	0.00486751	1.71485	1.71485	PF-00477736 up vs DMSO
26309	8117339	HIST1H3C	NM_003531	0.0411347	5.81755	5.81755	0.0411347	1.75124	1.75124	PF-00477736 up vs DMSO
27056	8124416	HIST1H3D	NM_003530	0.00791956	11.9829	11.9829	0.00791956	1.41756	1.41756	PF-00477736 up vs DMSO
27061	8124440	HIST1H3G	NM_003534	0.032981	8.48965	8.48965	0.032981	1.61726	1.61726	PF-00477736 up vs DMSO
27083	8124537	HIST1H3J	NM_003535	0.0220134	9.43732	9.43732	0.0220134	1.51517	1.51517	PF-00477736 up vs DMSO
27048	8124385	HIST1H4B	NM_003544	0.000213581	10.2261	10.2261	0.000213581	1.67093	1.67093	PF-00477736 up vs DMSO
26317	8117402	HIST1H4E	NM_003545	0.00263321	9.73097	9.73097	0.00263321	1.3328	1.3328	PF-00477736 up vs DMSO
26351	8117598	HIST1H4J	NM_021968	0.0123996	10.7835	10.7835	0.0123996	1.34237	1.34237	PF-00477736 up vs DMSO
27078	8124521	HIST1H4K	NM_003541	0.0149439	11.0634	11.0634	0.0149439	1.36941	1.36941	PF-00477736 up vs DMSO
5047	7905079	HIST2H2AA3	NM_003516	7.82E-05	10.8301	10.8301	7.82E-05	1.66106	1.66106	PF-00477736 up vs DMSO
6476	7919619	HIST2H2AA3	NM_003516	7.82E-05	10.8301	10.8301	7.82E-05	1.66106	1.66106	PF-00477736 up vs DMSO
6480	7919642	HIST2H2AB	NM_175065	0.0434652	11.7181	11.7181	0.0434652	1.33288	1.33288	PF-00477736 up vs DMSO
5049	7905088	HIST2H2AC	NM_003517	0.00138705	10.8791	10.8791	0.00138705	1.46123	1.46123	PF-00477736 up vs DMSO
6477	7919625	HIST2H2BC	NR_036461	0.01113	12.2046	12.2046	0.01113	1.18191	1.18191	PF-00477736 up vs DMSO
6473	7919606	HIST2H2BF	NM_001024599	0.00183873	7.29011	7.29011	0.00183873	2.02203	2.02203	PF-00477736 up vs DMSO
6464	7919584	HIST2H2BF	NM_001024599	0.00287831	6.3963	6.3963	0.00287831	2.18769	2.18769	PF-00477736 up vs DMSO
6472	7919604	HIST2H2BF	AK092418	0.0461241	4.60008	4.60008	0.0461241	1.17269	1.17269	PF-00477736 up vs DMSO
5045	7905067	HIST2H4A	NM_003548	0.00204775	8.01514	8.01514	0.00204775	3.53071	3.53071	PF-00477736 up vs DMSO
6478	7919627	HIST2H4A	NM_003548	0.00204775	8.01514	8.01514	0.00204775	3.53071	3.53071	PF-00477736 up vs DMSO
10731	7961483	HIST4H4	NM_175054	0.00402927	4.80632	4.80632	0.00402927	1.88883	1.88883	PF-00477736 up vs DMSO
18797	8042942	HK2	NM_000189	0.0291511	8.96013	8.96013	0.0291511	0.624827	-1.60044	PF-00477736 down vs DMSO
26478	8118794	HMGA1	NM_145899	0.0156076	9.40454	9.40454	0.0156076	0.719171	-1.39049	PF-00477736 down vs DMSO
5882	7913682	HMGCL	NM_000191	0.0202677	6.18153	6.18153	0.0202677	1.20395	1.20395	PF-00477736 up vs DMSO
13798	7992337	HN1L	NM_144570	0.0313452	8.89395	8.89395	0.0313452	0.797437	-1.25402	PF-00477736 down vs DMSO
12338	7977657	HNRNPC	NM_031314	0.0414806	11.4899	11.4899	0.0414806	0.883891	-1.13136	PF-00477736 down vs DMSO
7307	7927854	HNRNPH3	NM_012207	0.0350193	9.75177	9.75177	0.0350193	0.830775	-1.2037	PF-00477736 down vs DMSO
18133	8036613	HNRNPL	NM_001533	0.0487501	11.1527	11.1527	0.0487501	0.884942	-1.13002	PF-00477736 down vs DMSO
5872	7913571	HNRNPR	NM_001102398	0.000850876	10.4965	10.4965	0.000850876	0.805953	-1.24077	PF-00477736 down vs DMSO
7058	7925565	HNRNPU	NM_031844	0.00914131	10.9157	10.9157	0.00914131	0.874254	-1.14383	PF-00477736 down vs DMSO
31912	8169984	HPRT1	NM_000194	0.00921934	9.26904	9.26904	0.00921934	0.691862	-1.44537	PF-00477736 down vs DMSO
12701	7981335	HSP90AA1	NM_001017963	0.0184482	11.9868	11.9868	0.0184482	0.75912	-1.31732	PF-00477736 down vs DMSO
24882	8103722	HSP90AA6P	NR_036751	0.0371501	8.08011	8.08011	0.0371501	0.624741	-1.60066	PF-00477736 down vs DMSO
25321	8108015	HSPA4	NM_002154	0.00108044	10.0397	10.0397	0.00108044	0.732136	-1.36587	PF-00477736 down vs DMSO
9837	7952325	HSPA8	NM_006597	0.0206475	13.2263	13.2263	0.0206475	0.885531	-1.12927	PF-00477736 down vs DMSO
6133	7916356	HSPB11	NM_016126	0.0249599	8.1724	8.1724	0.0249599	0.715848	-1.39695	PF-00477736 down vs DMSO
20277	8058052	HSPD1	NM_002156	0.00588087	11.0786	11.0786	0.00588087	0.723075	-1.38298	PF-00477736 down vs DMSO
19275	8047223	HSPE1	NM_002157	0.0136729	8.74204	8.74204	0.0136729	0.630742	-1.58543	PF-00477736 down vs DMSO
25746	8112333	HTR1A	NM_000524	0.0332151	4.51602	4.51602	0.0332151	1.22905	1.22905	PF-00477736 up vs DMSO
10025	7954071	HTR7P1	NR_002774	0.0246221	5.57203	5.57203	0.0246221	1.16721	1.16721	PF-00477736 up vs DMSO
5497	7909815	IARS2	NM_018060	0.0387169	8.65926	8.65926	0.0387169	0.747604	-1.33761	PF-00477736 down vs DMSO
17035	8025601	ICAM1	NM_000201	0.0391904	6.51932	6.51932	0.0391904	1.87195	1.87195	PF-00477736 up vs DMSO
27123	8124848	IER3	NM_003897	0.0181219	7.3823	7.3823	0.0181219	2.50753	2.50753	PF-00477736 up vs DMSO
33033	8179704	IER3	NM_003897	0.0181219	7.3823	7.3823	0.0181219	2.50753	2.50753	PF-00477736 up vs DMSO
32905	8178435	IER3	NM_003897	0.0306624	7.37126	7.37126	0.0306624	2.41736	2.41736	PF-00477736 up vs DMSO
16792	8023175	IER3IP1	NM_016097	0.0428308	8.53423	8.53423	0.0428308	0.809288	-1.23565	PF-00477736 down vs DMSO
5337	7907904	IER5	NM_016545	4.43E-05	7.39178	7.39178	4.43E-05	1.34209	1.34209	PF-00477736 up vs DMSO
12197	7976436	IFI27L1	NM_145249	0.0295726	7.13827	7.13827	0.0295726	0.780135	-1.28183	PF-00477736 down vs DMSO
30850	8160371	IFNA21	NM_002175	0.0256498	2.78607	2.78607	0.0256498	1.08141	1.08141	PF-00477736 up vs DMSO
27780	8131860	IGF2BP3	NM_006547	0.0281024	9.46012	9.46012	0.0281024	0.760205	-1.31543	PF-00477736 down vs DMSO
19888	8053690	IGK@	BC032451	0.0145153	5.0527	5.0527	0.0145153	1.26043	1.26043	PF-00477736 up vs DMSO

18865	8043431	IGKC	AF113887	0.000855922	4.86182	4.86182	0.000855922	1.19266	1.19266	PF-00477736 up vs DMSO
24016	8095680	IL8	NM_000584	0.036437	5.3249	5.3249	0.036437	2.29928	2.29928	PF-00477736 up vs DMSO
28193	8135718	ING3	NM_019071	0.0144377	8.31991	8.31991	0.0144377	1.48456	1.48456	PF-00477736 up vs DMSO
10629	7960654	ING4	NM_016162	0.034378	8.23388	8.23388	0.034378	1.4915	1.4915	PF-00477736 up vs DMSO
11703	7971692	INTS6	NM_012141	0.0246469	6.84737	6.84737	0.0246469	1.28098	1.28098	PF-00477736 up vs DMSO
25083	8105545	IPO11	NM_016338	0.0303231	9.11903	9.11903	0.0303231	0.77117	-1.29673	PF-00477736 down vs DMSO
11497	7969703	IPO5	NM_002271	0.00988993	10.4093	10.4093	0.00988993	0.768135	-1.30185	PF-00477736 down vs DMSO
20451	8059878	IQCA1	NM_024726	0.0430483	3.65086	3.65086	0.0430483	0.936928	-1.06732	PF-00477736 down vs DMSO
9071	7945462	IRF7	NM_004031	0.0137811	6.85587	6.85587	0.0137811	1.2708	1.2708	PF-00477736 up vs DMSO
25297	8107814	ISOC1	NM_016048	0.0370262	8.43087	8.43087	0.0370262	0.649481	-1.53969	PF-00477736 down vs DMSO
19552	8050176	ITGB1BP1	NM_004763	0.0294936	8.29859	8.29859	0.0294936	0.759066	-1.31741	PF-00477736 down vs DMSO
18901	8043597	ITPRIPL1	NM_178495	0.0364927	5.62015	5.62015	0.0364927	0.786572	-1.27134	PF-00477736 down vs DMSO
6800	7922889	IVNS1ABP	NM_006469	0.0209311	9.32028	9.32028	0.0209311	0.775727	-1.28911	PF-00477736 down vs DMSO
17992	8035351	JAK3	NM_000215	0.0131008	6.75287	6.75287	0.0131008	1.44871	1.44871	PF-00477736 up vs DMSO
6160	7916609	JUN	NM_002228	0.000932141	5.9837	5.9837	0.000932141	5.3469	5.3469	PF-00477736 up vs DMSO
17077	8026047	JUNB	NM_002229	0.0118988	7.7061	7.7061	0.0118988	1.75993	1.75993	PF-00477736 up vs DMSO
17997	8035445	JUND	NM_005354	0.043722	8.10539	8.10539	0.043722	1.64645	1.64645	PF-00477736 up vs DMSO
8663	7941382	KAT5	NM_182710	0.046845	7.90823	7.90823	0.046845	1.15248	1.15248	PF-00477736 up vs DMSO
5803	7912629	KAZ	NM_201628	0.031254	5.22826	5.22826	0.031254	0.930861	-1.07427	PF-00477736 down vs DMSO
4614	7900833	KDM4A	NM_014663	0.00514289	8.1768	8.1768	0.00514289	1.30271	1.30271	PF-00477736 up vs DMSO
14967	8004671	KDM6B	NM_001080424	0.00554689	7.55016	7.55016	0.00554689	1.66623	1.66623	PF-00477736 up vs DMSO
18606	8040802	KHK	NM_000221	0.0251551	6.60901	6.60901	0.0251551	0.756622	-1.32166	PF-00477736 down vs DMSO
11929	7973732	KHNYN	NM_015299	0.046657	6.07137	6.07137	0.046657	1.18675	1.18675	PF-00477736 up vs DMSO
15840	8013696	KIAA0100	NM_014680	0.00429884	9.46555	9.46555	0.00429884	0.810469	-1.23385	PF-00477736 down vs DMSO
13532	7989647	KIAA0101	NM_014736	0.0438412	10.7407	10.7407	0.0438412	0.628048	-1.59224	PF-00477736 down vs DMSO
24957	8104350	KIAA0947	NM_015325	0.0401241	8.61422	8.61422	0.0401241	0.854184	-1.17071	PF-00477736 down vs DMSO
23400	8089659	KIAA1407	AK302488	0.00906553	4.64786	4.64786	0.00906553	1.41472	1.41472	PF-00477736 up vs DMSO
17996	8035435	KIAA1683	NM_001145304	0.0458675	5.78532	5.78532	0.0458675	1.22676	1.22676	PF-00477736 up vs DMSO
14918	8004057	KIF1C	NM_006612	0.0307925	7.25728	7.25728	0.0307925	0.812764	-1.23037	PF-00477736 down vs DMSO
6832	7923233	KIF21B	NM_017596	0.0257258	7.64913	7.64913	0.0257258	1.36853	1.36853	PF-00477736 up vs DMSO
17733	8032365	KLF16	NM_031918	0.0156401	8.55352	8.55352	0.0156401	0.709622	-1.4092	PF-00477736 down vs DMSO
17136	8026564	KLF2	NM_016270	0.0416304	7.11067	7.11067	0.0416304	1.11165	1.11165	PF-00477736 up vs DMSO
7661	7931810	KLF6	NM_001300	8.41E-05	7.30321	7.30321	8.41E-05	2.17448	2.17448	PF-00477736 up vs DMSO
26556	8119648	KLHDC3	NM_057161	0.0412531	9.95736	9.95736	0.0412531	0.815715	-1.22592	PF-00477736 down vs DMSO
22902	8084219	KLHL24	NM_017644	0.0187323	7.99444	7.99444	0.0187323	1.7793	1.7793	PF-00477736 up vs DMSO
21464	8069827	KRTAP19-2	NM_181608	0.0240666	3.42336	3.42336	0.0240666	0.924443	-1.08173	PF-00477736 down vs DMSO
32616	8176093	LAGE3	NM_006014	0.0264532	7.55732	7.55732	0.0264532	0.731464	-1.36712	PF-00477736 down vs DMSO
23858	8094259	LAP3	NM_015907	0.0498473	8.04866	8.04866	0.0498473	0.819031	-1.22095	PF-00477736 down vs DMSO
30748	8159275	LCN9	NM_001001676	0.038244	3.91949	3.91949	0.038244	0.934836	-1.06971	PF-00477736 down vs DMSO
8372	7938777	LDHA	NM_005566	0.00698046	12.0032	12.0032	0.00698046	0.634567	-1.57588	PF-00477736 down vs DMSO
18413	8039139	LENG1	NM_024316	0.0073856	5.94074	5.94074	0.0073856	1.27048	1.27048	PF-00477736 up vs DMSO
13471	7988838	LEO1	NM_138792	0.0128768	8.62665	8.62665	0.0128768	0.792382	-1.26202	PF-00477736 down vs DMSO
28210	8135909	LEP	NM_000230	0.030361	5.47268	5.47268	0.030361	1.14184	1.14184	PF-00477736 up vs DMSO
18365	8038815	LIM2	NM_030657	0.0488367	5.03955	5.03955	0.0488367	1.1281	1.1281	PF-00477736 up vs DMSO
10859	7963024	LMBR1L	NM_018113	0.00644508	7.40362	7.40362	0.00644508	1.26842	1.26842	PF-00477736 up vs DMSO
5713	7911718	LOC100129534	NR_024489	0.0451958	5.65142	5.65142	0.0451958	1.22017	1.22017	PF-00477736 up vs DMSO
19548	8050113	LOC100129581	AK125905	0.0248198	5.72844	5.72844	0.0248198	0.866183	-1.15449	PF-00477736 down vs DMSO
14787	8002760	LOC100132346	ENST00000412576	0.0483117	6.69911	6.69911	0.0483117	0.681078	-1.46826	PF-00477736 down vs DMSO
8654	7941269	LOC100291851	ENST00000309775	0.0135662	6.74443	6.74443	0.0135662	0.886917	-1.1275	PF-00477736 down vs DMSO
4933	7904082	LOC128322	XM_001716411	0.046695	6.22629	6.22629	0.046695	0.807713	-1.23806	PF-00477736 down vs DMSO
18818	8043036	LOC1720	NR_033423	0.0335124	9.76685	9.76685	0.0335124	0.754659	-1.3251	PF-00477736 down vs DMSO
15428	8009094	LOC342541	ENST00000416490	0.0348965	8.04535	8.04535	0.0348965	0.834158	-1.19881	PF-00477736 down vs DMSO
11330	7968056	LOC374491	NR_002815	0.00949123	3.82145	3.82145	0.00949123	1.09475	1.09475	PF-00477736 up vs DMSO
14150	7996160	LOC388282	ENST00000335616	0.0419155	4.97592	4.97592	0.0419155	1.10984	1.10984	PF-00477736 up vs DMSO
18520	8040077	LOC400940	NR_026833	0.0360237	3.87227	3.87227	0.0360237	1.12432	1.12432	PF-00477736 up vs DMSO
20453	8059902	LOC93463	AL122100	0.0181868	4.59834	4.59834	0.0181868	1.10351	1.10351	PF-00477736 up vs DMSO
28848	8141535	LRCH4	NM_002319	0.000634073	7.19613	7.19613	0.000634073	1.23764	1.23764	PF-00477736 up vs DMSO
18142	8036707	LRFN1	NM_020862	0.0413699	6.33623	6.33623	0.0413699	0.758	-1.31926	PF-00477736 down vs DMSO
28253	8136315	LRGUK	NM_144648	0.0291266	4.66012	4.66012	0.0291266	1.36692	1.36692	PF-00477736 up vs DMSO
4694	7901577	LRRC42	NM_052940	0.0192894	8.2777	8.2777	0.0192894	0.766293	-1.30498	PF-00477736 down vs DMSO
13404	7987916	LRRC57	NM_153260	0.0170259	7.15224	7.15224	0.0170259	1.19565	1.19565	PF-00477736 up vs DMSO
27153	8125125	LSM2	NM_021177	0.0456249	8.60919	8.60919	0.0456249	0.734026	-1.36235	PF-00477736 down vs DMSO
32926	8178641	LSM2	NM_021177	0.0456249	8.60919	8.60919	0.0456249	0.734026	-1.36235	PF-00477736 down vs DMSO
33050	8179839	LSM2	NM_021177	0.0456249	8.60919	8.60919	0.0456249	0.734026	-1.36235	PF-00477736 down vs DMSO
21583	8070961	LSS	NM_002340	0.00969321	8.34759	8.34759	0.00969321	0.610593	-1.63775	PF-00477736 down vs DMSO
10062	7954460	LYRM5	NM_001001660	0.0421667	7.92526	7.92526	0.0421667	1.18733	1.18733	PF-00477736 up vs DMSO
10667	7960933	M6PR	NM_002355	0.0317937	9.96641	9.96641	0.0317937	0.775884	-1.28885	PF-00477736 down vs DMSO
21799	8073007	MAFF	NM_012323	0.0185345	5.17878	5.17878	0.0185345	1.15646	1.15646	PF-00477736 up vs DMSO
10693	7961208	MAGOHB	NM_018048	0.0434457	8.62102	8.62102	0.0434457	0.82251	-1.21579	PF-00477736 down vs DMSO
26699	8121144	MANEA	NM_024641	0.03173	8.8404	8.8404	0.03173	0.732654	-1.3649	PF-00477736 down vs DMSO
14298	7997740	MAP1LC3B	NM_022818	0.000626127	6.50448	6.50448	0.000626127	2.04974	2.04974	PF-00477736 up vs DMSO
31261	8164177	MAPKAP1	NM_001006617	0.00660394	9.28812	9.28812	0.00660394	0.770288	-1.29822	PF-00477736 down vs DMSO
16094	8016263	MAPT	NM_016835	0.0289398	5.11276	5.11276	0.0289398	1.15567	1.15567	PF-00477736 up vs DMSO
18834	8043187	MAT2A	NM_005911	0.0237438	11.6443	11.6443	0.0237438	0.805075	-1.24212	PF-00477736 down vs DMSO
17187	8027184	MAU2	NM_015329	0.00317168	8.45566	8.45566	0.00317168	1.28051	1.28051	PF-00477736 up vs DMSO
14009	7994637	MAZ	NM_001042539	0.0378781	8.54846	8.54846	0.0378781	0.884338	-1.13079	PF-00477736 down vs DMSO
10264	7956470	MBD6	NM_052897	0.00896192	7.81912	7.81912	0.00896192	1.19257	1.19257	PF-00477736 up vs DMSO
22709	8082350	MCM2	NM_004526	0.0303043	9.80165	9.80165	0.0303043	0.699849	-1.42888	PF-00477736 down vs DMSO
27328	8127031	MCM3	NM_002388	0.0384981	10.5511	10.5511	0.0384981	0.767667	-1.30265	PF-00477736 down vs DMSO
21767	8072687	MCM5	NM_006739	0.0155688	9.99016	9.99016	0.0155688	0.735882	-1.35891	PF-00477736 down vs DMSO
20085	8055426	MCM6	NM_005915	0.0200566	9.78709	9.78709	0.0200566	0.734218	-1.36199	PF-00477736 down vs DMSO
18728	8042259	MDH1	NM_005917	0.0149784	9.14441	9.14441	0.0149784	0.798188	-1.25284	PF-00477736 down vs DMSO
17714	8032106	MED16	NM_005481	0.0207336	8.288	8.288	0.0207336	0.882049	-1.13372	PF-00477736 down vs DMSO
9403	7948293	MED19	NM_153450	0.04832	6.3719	6.3719	0.04832	1.16467	1.16467	PF-00477736 up vs DMSO
17339	8028645	MED29	NM_017592	0.0119726	8.04259	8.04259	0.0119726	1.2204	1.2204	PF-00477736 up vs DMSO
18274	8037878	MEIS3	NM_020160	0.0269124	6.09292	6.09292	0.0269124	0.87817	-1.13873	PF-00477736 down vs DMSO
19653	8051372	MEMO1	NM_015955	0.0218596	7.90564	7.90564	0.0218596	0.882172	-1.13357	PF-00477736 down vs DMSO
21330	8068410	MEMO1	NM_015955	0.0403903	7.89566	7.89566	0.0403903	0.899105	-1.11222	PF-00477736 down vs DMSO
24900	8103853	MGC45800	NR_027107	0.0397349	5.22888	5.22888	0.0397349	1.14249	1.14249	PF-00477736 up vs DMSO
5256	7906978	MGST3	NM_004528	0.0259147	9.61887	9.61887	0.0259147	0.655961	-1.52448	PF-00477736 down vs DMSO
21674	8071737	MIF	NM_002415	0.0159243	10.7954	10.7954	0.0159243	0.68887	-1.45165	PF-00477736 down vs DMSO
11482	7969576	MIR17HG	NR_027350	0.0302224	7.07504	7.07504	0.0302224	0.663122	-1.50802	PF-00477736 down vs DMSO
31281	8164396	MIR199B	NR_029619	0.0319207	3.0331	3.0331	0.0319207	1.12734	1.12734	PF-00477736 up vs DMSO
19385	8048317	MIR26B	NR_029500	0.0370609	4.30012	4.30012	0.0370609	1.09892	1.09892	PF-00477736 up vs DMSO
29741	8149705	MIR320A	NR_029714	0.000514333	6.87299	6.87299	0.000514333	1.52402	1.52402	PF-00477736 up vs DMSO
23173	8087250	MIR425	NR_029948	0.0183971	5.16047	5.16047	0.0183971	1.26639	1.26639	PF-00477736 up vs DMSO
10509	7959205	MLEC	NM_014730	0.00905339	9.55255	9.55255	0.00905339	0.817403	-1.22339	PF-00477736 down vs DMSO
15932	8014702	MLLT6	NM_005937	0.019959	5.82885	5.82885	0.019959	1.23108	1.23108	PF-00477736 up vs DMSO
32530	8175360	MMGT1	NM_173470	0.0247137	8.90083	8.90083	0.0247137	0.814171	-1.22824	PF-00477736 down vs DMSO
9723	7951246	MMP8	NM_002424	0.029461	3.18098	3.18098	0.029461	1.28926	1.28926	PF-00477736 up vs DMSO
19820	8053064	MOBKL1B	NM_018221	0.0326554	10.8181	10.8181	0.0326554	0.893285	-1.11946	PF-00477736 down vs DMSO
8771	7942603	MOGAT2	NM_025098	0.0266602	4.88104	4.88104	0.0266602	1.09848	1.09848	PF-00477736 up vs DMSO
28859	8141676	MOGAT3	NM_178176	0.0336379	4.76472	4.76472	0.0336379	1.18617	1.18617	PF-00477736 up vs DMSO
5712	7911701	MORN1	NM_024848	0.0155427	6.13836	6.13836	0.0155427	1.12189	1.12189	PF-00477736 up vs DMSO
8036	7935510	MORN4	NM_178832	0.0285281	4.24896	4.24896	0.0285281	0.779961	-1.28212	PF-00477736 down vs DMSO
17168	8026982	MPV17L2	NM_032683	0.0376518	8.9398	8.9398	0.0376518	0.804442	-1.2431	PF-00477736 down vs DMSO
15185	8006668	MRM1	NM_024864	0.034497	6.88856	6.88856	0.034497	0.738049	-1.35492	PF-00477736 down vs DMSO
15558	8010664	MRPL12	NM_002949	0.0411139	8.97523	8.97523	0.0411139	0.814703	-1.22744	PF-00477736 down vs DMSO
9183	7946267	MRPL17	NM_022061	0.0374088	9.54699	9.54699	0.0374088	0.765695	-1.306	PF-00477736 down vs DMSO
26918	8123148	MRPL18	NM_014161	0.0454849	9.61204	9.61204	0.0454849	0.776648	-1.28758	PF-00477736 down vs DMSO
18801	8042962	MRPL19	NM_014763	0.040266	8.16989	8.16989	0.040266	0.840899	-1.1892	PF-00477736 down vs DMSO
17034	8025586	MRPL4	NM_146388	0.0260121	8.01988	8.01988	0.0260121	0.765281	-1.30671	PF-00477736 down vs DMSO
14375	7998585	MRPS34	NM_023936	0.0257856	8.96665	8.96665	0.0257856	0.799616	-1.2506	PF-00477736 down vs DMSO
9435	7948455	MS4A6A	NM_152852	0.0103145	3.98595	3.98595	0.0103145	1.13089	1.13089	PF-00477736 up vs DMSO
4366	7898357	MST1P2	NR_027504	0.0181424	5.50611	5.50611	0.0181424	1.10038	1.10038	PF-00477736 up vs DMSO
5829	7912863	MST1P9	NR_002729	0.0412796	5.64294	5.64294	0.0412796	1.08588	1.08588	PF-00477736 up vs DMSO
20478	8060196	MTERFD2	NR_028049	0.017934	6.46485	6.46485	0.017934	1.24432	1.24432	PF-00477736 up vs DMSO
12065	7975045	MTHFD1	NM_005956	0.0348006	9.18997	9.18997	0.0348006	0.682535	-1.46513	PF-00477736 down vs DMSO
16177	8017019	MTMR4	NM_004687	0.0371033	9.67314	9.67314	0.0371033	0.824905	-1.21226	PF-00477736 down vs DMSO
8666	7941425	MUS81	NM_025128	0.0207167	7.57134	7.57134	0.0207167	1.09969	1.09969	PF-00477736 up vs DMSO
30562	8157173	MUSK	NM_005592	0.0326566	3.67527	3.67527	0.0326566	0.934793	-1.06976	PF-00477736 down vs DMSO
14844	8003332	MVD	NM_002461	0.0396409	7.90888	7.90888	0.0396409	0.862443	-1.1595	PF-00477736 down vs DMSO
14012	7994659	MVP	NM_017458	0.0155009	5.80895	5.80895	0.0155009	1.18641	1.18641	PF-00477736 up vs DMSO
10447	7958524	MYO1H	NM_001101421	0.0249555	3.54181	3.54181	0.0249555	1.13622	1.13622	PF-00477736 up vs DMSO
32605	8175924	NAA10	NM_003491	0.0275382	9.24854	9.24854	0.0275382	0.769779	-1.29907	PF-00477736 down vs DMSO
24199	8097480	NAA15	NM_057175	0.0305753	9.55859	9.55859	0.0305753	0.741504	-1.34861	PF-00477736 down vs DMSO
9104	7945786	NAP1L4	NM_005969	0.0369944	9.04956	9.04956	0.0369944	0.895967	-1.11611	PF-00477736 down vs DMSO
15574	8010804	NARF	NM_001038618	0.0470734	9.30157	9.30157	0.0470734	0.774017	-1.29196	PF-00477736 down vs DMSO
20426	8059689	NCL	NM_005381	0.0158189	11.5857	11.5857	0.0158189	0.773352	-1.29307	PF-00477736 down vs DMSO
20773	8063211	NCOA3	NM_181659	0.0145151	8.84103	8.84103	0.0145151	1.32494	1.32494	PF-00477736 up vs DMSO
26794	8121861	NCOA7	NM_181782	0.036833	7.31603	7.31603	0.036833	1.29484	1.29484	PF-00477736 up vs DMSO
21221	8067588	NCRNA00029	NR_028295	0.0492371	4.35955	4.35955	0.0492371	1.16593	1.16593	PF-00477736 up vs DMSO
9043	7945177	NCRNA00167	NR_024233	0.0276419	4.78217	4.78217	0.0276419	1.14181	1.14181	PF-00477736 up vs DMSO
20779	8063337	NCRNA00275	NR_003604	0.0232951	8.73637	8.73637	0.0232951	1.29926	1.29926	PF-00477736 up vs DMSO
24731	8102450	NDST4	NM_022569	0.0394477	3.29977	3.29977	0.0394477	1.10645	1.10645	PF-00477736 up vs DMSO
31878	8169659	NDUFA1	NM_004541	0.0393267	8.62287	8.62287	0.0393267	0.829179	-1.20601	PF-00477736 down vs DMSO
26016	8114618	NDUFA2	NM_002488	0.0156869	8.98667	8.98667	0.0156869	0.734655	-1.36118	PF-00477736 down vs DMSO
14540	8000323	NDUFAB1	NM_005003	0.000575343	8.79471	8.79471	0.000575343	0.702099	-1.4243	PF-00477736 down vs DMSO
28279	8136602	NDUFB2	NM_004546	0.0250867	7.58845	7.58845	0.0250867	0.85776	-1.16583	PF-00477736 down vs DMSO

24950	8104298	NDUFS6	NM_004553	0.0148435	8.95223	8.95223	0.0148435	0.794616	-1.25847	PF-00477736 down vs DMSO
18400	8039068	NDUFV2	NM_021074	0.0199335	9.82345	9.82345	0.0199335	0.791739	-1.26304	PF-00477736 down vs DMSO
16464	8020058	NDUFV2	NM_021074	0.0200259	9.80199	9.80199	0.0200259	0.784289	-1.27504	PF-00477736 down vs DMSO
27155	8125139	NEU1	NM_000434	0.0282531	7.57229	7.57229	0.0282531	1.39516	1.39516	PF-00477736 up vs DMSO
33052	8179851	NEU1	NM_000434	0.0282531	7.57229	7.57229	0.0282531	1.39516	1.39516	PF-00477736 up vs DMSO
32929	8178676	NEU1	NM_000434	0.0437996	7.93464	7.93464	0.0437996	1.37291	1.37291	PF-00477736 up vs DMSO
7515	7930074	NFKB2	NM_002502	0.00626564	6.74776	6.74776	0.00626564	2.26153	2.26153	PF-00477736 up vs DMSO
22617	8081386	NFKBIZ	NM_031419	0.0181783	5.68879	5.68879	0.0181783	2.09757	2.09757	PF-00477736 up vs DMSO
7437	7929243	NHP2	NM_017838	0.0426055	9.4632	9.4632	0.0426055	0.745203	-1.34192	PF-00477736 down vs DMSO
7028	7925320	NID1	NM_002508	0.00488302	4.20268	4.20268	0.00488302	1.18252	1.18252	PF-00477736 up vs DMSO
15366	8008517	NME1	NM_198175	0.00491297	10.3588	10.3588	0.00491297	0.634141	-1.57694	PF-00477736 down vs DMSO
31088	8162352	NOL8	NR_024020	0.0261281	9.46031	9.46031	0.0261281	0.831229	-1.20304	PF-00477736 down vs DMSO
28400	8137584	NOM1	NM_138400	0.0372481	8.43781	8.43781	0.0372481	0.781893	-1.27895	PF-00477736 down vs DMSO
26149	8115907	NOP16	NM_016391	0.0496477	7.81944	7.81944	0.0496477	0.694525	-1.43983	PF-00477736 down vs DMSO
11219	7966779	NOS1	NM_000620	0.00836457	4.60735	4.60735	0.00836457	1.18093	1.18093	PF-00477736 up vs DMSO
15323	8007976	NPEPPS	NM_006310	0.0239849	9.72501	9.72501	0.0239849	0.800317	-1.2495	PF-00477736 down vs DMSO
17982	8035249	NR2F6	NM_005234	0.0309747	7.74193	7.74193	0.0309747	0.812183	-1.23125	PF-00477736 down vs DMSO
30527	8156848	NR4A3	NM_006981	0.00125595	5.27121	5.27121	0.00125595	1.3768	1.3768	PF-00477736 up vs DMSO
32902	8178399	NRM	NM_007243	0.0427639	8.94871	8.94871	0.0427639	0.649332	-1.54004	PF-00477736 down vs DMSO
33031	8179683	NRM	NM_007243	0.0427639	8.94871	8.94871	0.0427639	0.649332	-1.54004	PF-00477736 down vs DMSO
22576	8081128	NSUN3	NM_022072	0.0355264	7.75996	7.75996	0.0355264	1.19343	1.19343	PF-00477736 up vs DMSO
11149	7966026	NUAK1	NM_014840	0.0162129	4.13576	4.13576	0.0162129	0.865339	-1.15562	PF-00477736 down vs DMSO
7689	7932069	NUDT5	NM_014142	0.0348946	9.68629	9.68629	0.0348946	0.83481	-1.19788	PF-00477736 down vs DMSO
10305	7956949	NUP107	NM_020401	0.0196789	8.74871	8.74871	0.0196789	0.713309	-1.40192	PF-00477736 down vs DMSO
9348	7947991	NUP160	NM_015231	0.0432497	9.33974	9.33974	0.0432497	0.820625	-1.21858	PF-00477736 down vs DMSO
30678	8158446	NUP188	NM_015354	0.00742775	9.89229	9.89229	0.00742775	0.71317	-1.40219	PF-00477736 down vs DMSO
28260	8136401	NUP205	NM_015135	0.0190509	9.60294	9.60294	0.0190509	0.773639	-1.29259	PF-00477736 down vs DMSO
11134	7965855	NUP37	NM_024057	0.041587	8.74612	8.74612	0.041587	0.669755	-1.49308	PF-00477736 down vs DMSO
27610	8130129	NUP43	NM_198887	0.0137035	10.1115	10.1115	0.0137035	0.823166	-1.21482	PF-00477736 down vs DMSO
26772	8121704	NUS1	NM_138459	0.0330939	10.6812	10.6812	0.0330939	0.815625	-1.22605	PF-00477736 down vs DMSO
14196	7996677	NUTF2	NM_005796	0.0248642	11.1616	11.1616	0.0248642	0.782236	-1.27839	PF-00477736 down vs DMSO
27733	8131349	OCM	NM_001097622	0.0233235	5.13203	5.13203	0.0233235	1.10973	1.10973	PF-00477736 up vs DMSO
26986	8123843	OFCC1	AF520800	0.0452024	3.0745	3.0745	0.0452024	1.06812	1.06812	PF-00477736 up vs DMSO
7145	7926334	OLAH	NM_018324	0.0270921	3.71411	3.71411	0.0270921	1.68853	1.68853	PF-00477736 up vs DMSO
7138	7926239	OPTN	NM_001008211	0.014091	6.24495	6.24495	0.014091	1.45753	1.45753	PF-00477736 up vs DMSO
31152	8162936	OR13C2	NM_001004481	0.0328886	3.01725	3.01725	0.0328886	1.18055	1.18055	PF-00477736 up vs DMSO
5656	7911271	OR14C36	NM_001001918	0.0436482	4.71149	4.71149	0.0436482	0.871549	-1.14738	PF-00477736 down vs DMSO
27100	8124632	OR2B3	NM_001005226	0.0403527	3.65552	3.65552	0.0403527	1.16712	1.16712	PF-00477736 up vs DMSO
32887	8178285	OR2B3	NM_001005226	0.0403527	3.65552	3.65552	0.0403527	1.16712	1.16712	PF-00477736 up vs DMSO
33022	8179589	OR2B3	NM_001005226	0.0403527	3.65552	3.65552	0.0403527	1.16712	1.16712	PF-00477736 up vs DMSO
5635	7911207	OR2G2	NM_001001915	0.0264105	4.49366	4.49366	0.0264105	0.885787	-1.12894	PF-00477736 down vs DMSO
8480	7939918	OR4A15	NM_001005275	0.0183847	3.29272	3.29272	0.0183847	1.16695	1.16695	PF-00477736 up vs DMSO
8548	7940177	OR4D10	NM_001004705	0.0355228	3.99394	3.99394	0.0355228	0.869194	-1.15049	PF-00477736 down vs DMSO
4213	7896740	OR4F17	NM_001005240	0.0356364	2.98762	2.98762	0.0356364	1.2266	1.2266	PF-00477736 up vs DMSO
13737	7991663	OR4F17	NM_001005240	0.0356364	2.98762	2.98762	0.0356364	1.2266	1.2266	PF-00477736 up vs DMSO
16880	8023937	OR4F17	NM_001005240	0.0356364	2.98762	2.98762	0.0356364	1.2266	1.2266	PF-00477736 up vs DMSO
9131	7945991	OR51E2	NM_030774	0.00149301	3.86368	3.86368	0.00149301	0.866384	-1.15422	PF-00477736 down vs DMSO
8275	7938008	OR52D1	NM_001005163	0.00962391	4.35866	4.35866	0.00962391	0.865002	-1.15607	PF-00477736 down vs DMSO
9134	7946001	OR52R1	NM_001005177	0.0154181	4.75823	4.75823	0.0154181	1.1174	1.1174	PF-00477736 up vs DMSO
10215	7955995	OR6C65	NM_001005518	0.0428967	3.59888	3.59888	0.0428967	1.1679	1.1679	PF-00477736 up vs DMSO
9843	7952373	OR6X1	NM_001005188	0.0416722	4.24522	4.24522	0.0416722	1.08972	1.08972	PF-00477736 up vs DMSO
17843	8033736	OR7G1	NM_001005192	0.0213331	4.07544	4.07544	0.0213331	0.889511	-1.12421	PF-00477736 down vs DMSO
17844	8033738	OR7G3	NM_001001958	0.0266527	3.93617	3.93617	0.0266527	1.11639	1.11639	PF-00477736 up vs DMSO
15483	8009705	OTOP2	NM_178160	0.0386263	4.9796	4.9796	0.0386263	1.25515	1.25515	PF-00477736 up vs DMSO
8521	7940005	P2RX3	NM_002559	0.00795897	4.53392	4.53392	0.00795897	1.15493	1.15493	PF-00477736 up vs DMSO
10236	7956152	PA2G4	NM_006191	0.0378106	11.9524	11.9524	0.0378106	0.775398	-1.28966	PF-00477736 down vs DMSO
32261	8173168	PAGE3	NR_033460	0.0407776	3.64875	3.64875	0.0407776	0.918494	-1.08874	PF-00477736 down vs DMSO
23955	8095221	PAICS	NM_001079525	0.00240582	10.0815	10.0815	0.00240582	0.637927	-1.56758	PF-00477736 down vs DMSO
22975	8084963	PAK2	NM_002577	0.0461975	9.94981	9.94981	0.0461975	0.803421	-1.24468	PF-00477736 down vs DMSO
10962	7964089	PAN2	NM_001127460	0.0438232	8.206	8.206	0.0438232	1.4144	1.4144	PF-00477736 up vs DMSO
13578	7990165	PARP6	NM_020214	0.0207316	9.00525	9.00525	0.0207316	1.24753	1.24753	PF-00477736 up vs DMSO
29099	8144047	PAXIP1	NM_007349	0.0132755	9.01499	9.01499	0.0132755	0.817245	-1.22362	PF-00477736 down vs DMSO
21138	8066637	PCIF1	ENST00000372409	0.0481965	6.34066	6.34066	0.0481965	1.18058	1.18058	PF-00477736 up vs DMSO
20927	8064844	PCNA	NM_002592	0.046601	10.2769	10.2769	0.046601	0.777797	-1.28568	PF-00477736 down vs DMSO
18761	8042519	PCYOX1	NM_016297	0.00419744	8.03719	8.03719	0.00419744	0.775452	-1.28957	PF-00477736 down vs DMSO
24095	8096528	PDHA2	NM_005390	0.0493354	5.04569	5.04569	0.0493354	0.846746	-1.18099	PF-00477736 down vs DMSO
19200	8046408	PDK1	NM_002610	0.0129385	8.84821	8.84821	0.0129385	0.592518	-1.68771	PF-00477736 down vs DMSO
7188	7926807	PDSS1	NM_014317	0.0102212	9.49539	9.49539	0.0102212	0.711833	-1.40482	PF-00477736 down vs DMSO
29874	8150901	PENK	NM_006211	0.0269655	5.49796	5.49796	0.0269655	1.14414	1.14414	PF-00477736 up vs DMSO
14975	8004804	PFAS	NM_012393	0.0181055	8.66162	8.66162	0.0181055	0.705339	-1.41776	PF-00477736 down vs DMSO
23160	8086961	PFKFB4	NM_004567	0.002696	5.93476	5.93476	0.002696	0.615014	-1.62598	PF-00477736 down vs DMSO
7474	7929624	PGAM1	NM_002629	0.0142801	10.5106	10.5106	0.0142801	0.706634	-1.41516	PF-00477736 down vs DMSO
10416	7958152	PGAM1	NM_002629	0.0150013	10.5714	10.5714	0.0150013	0.708793	-1.41085	PF-00477736 down vs DMSO
11096	7965480	PGAM1	NM_002629	0.0150597	10.706	10.706	0.0150597	0.711905	-1.40468	PF-00477736 down vs DMSO
32329	8173729	PGAM4	NM_001029891	0.0124812	10.1259	10.1259	0.0124812	0.733399	-1.36351	PF-00477736 down vs DMSO
32639	8176282	PGAM4	NM_001029891	0.0124812	10.1259	10.1259	0.0124812	0.733399	-1.36351	PF-00477736 down vs DMSO
18994	8044640	PGM5	NM_021965	0.043017	5.97031	5.97031	0.043017	1.16376	1.16376	PF-00477736 up vs DMSO
30996	8161513	PGM5P2	NR_002836	0.0166513	4.45994	4.45994	0.0166513	1.14881	1.14881	PF-00477736 up vs DMSO
30997	8161520	PGM5P2	NR_002836	0.0261494	4.24737	4.24737	0.0261494	1.17549	1.17549	PF-00477736 up vs DMSO
24762	8102745	PGRMC2	NM_006320	0.0105313	8.23136	8.23136	0.0105313	0.810769	-1.2334	PF-00477736 down vs DMSO
4271	7897322	PHF13	NM_153812	0.00497425	6.99248	6.99248	0.00497425	0.839075	-1.19179	PF-00477736 down vs DMSO
15676	8012099	PHF23	NM_024297	0.017153	7.19698	7.19698	0.017153	0.825201	-1.21183	PF-00477736 down vs DMSO
15182	8006634	PIGW	NM_178517	0.00767303	8.30192	8.30192	0.00767303	0.581448	-1.71984	PF-00477736 down vs DMSO
22061	8075483	PIK3IP1	NM_052880	0.029878	4.97091	4.97091	0.029878	1.31135	1.31135	PF-00477736 up vs DMSO
10562	7959856	PIWIL1	NM_004764	0.0460715	4.0286	4.0286	0.0460715	0.902862	-1.10759	PF-00477736 down vs DMSO
22013	8075026	PIWIL3	NM_001008496	0.0282581	4.04228	4.04228	0.0282581	0.901092	-1.10976	PF-00477736 down vs DMSO
5399	7908672	PKP1	NM_000299	0.0400659	4.96821	4.96821	0.0400659	0.921671	-1.08499	PF-00477736 down vs DMSO
18283	8037970	PLA2G4C	NM_003706	0.0323287	4.34553	4.34553	0.0323287	1.7354	1.7354	PF-00477736 up vs DMSO
8626	7940924	PLCB3	NM_000932	0.0439049	7.11376	7.11376	0.0439049	0.797141	-1.25448	PF-00477736 down vs DMSO
17740	8032455	PLEKHJ1	NM_018049	0.031922	7.99093	7.99093	0.031922	0.873999	-1.14417	PF-00477736 down vs DMSO
4635	7901054	PLK3	NM_004073	0.0050855	6.63898	6.63898	0.0050855	1.70542	1.70542	PF-00477736 up vs DMSO
30751	8159337	PMPCA	NM_015160	0.0297602	8.22812	8.22812	0.0297602	0.864571	-1.15664	PF-00477736 down vs DMSO
25791	8112709	POC5	NM_001099271	0.038812	7.97935	7.97935	0.038812	1.37031	1.37031	PF-00477736 up vs DMSO
6514	7920000	POGZ	NM_015100	0.0330553	9.88541	9.88541	0.0330553	1.24643	1.24643	PF-00477736 up vs DMSO
17525	8030641	POLD1	NM_002691	0.0138999	8.11494	8.11494	0.0138999	0.753188	-1.32769	PF-00477736 down vs DMSO
28594	8139299	POLD2	NM_001127218	0.00512995	10.1192	10.1192	0.00512995	0.752199	-1.32943	PF-00477736 down vs DMSO
31202	8163525	POLE3	NM_017443	0.0175938	8.81285	8.81285	0.0175938	0.779259	-1.28327	PF-00477736 down vs DMSO
22153	8076344	POLR3H	NM_001018050	0.04162	7.60535	7.60535	0.04162	0.81555	-1.22617	PF-00477736 down vs DMSO
23413	8089785	POPDC2	NM_022135	0.000589213	3.80207	3.80207	0.000589213	1.61662	1.61662	PF-00477736 up vs DMSO
25431	8109179	PPARGC1B	NM_133263	0.00669808	7.21974	7.21974	0.00669808	0.826211	-1.21034	PF-00477736 down vs DMSO
24531	8100495	PPAT	NM_002703	0.00968934	9.08366	9.08366	0.00968934	0.711074	-1.40632	PF-00477736 down vs DMSO
21270	8067978	PPIA	NM_021130	0.0493901	12.234	12.234	0.0493901	0.85507	-1.16949	PF-00477736 down vs DMSO
6449	7919436	PPIAL4A	NM_178230	0.0368039	12.2736	12.2736	0.0368039	0.886888	-1.12754	PF-00477736 down vs DMSO
5028	7904997	PPIAL4A	NM_178230	0.0425412	6.7136	6.7136	0.0425412	0.904085	-1.10609	PF-00477736 down vs DMSO
5030	7905016	PPIAL4A	NM_178230	0.0425412	6.7136	6.7136	0.0425412	0.904085	-1.10609	PF-00477736 down vs DMSO
4598	7900576	PPIH	NM_006347	0.0433062	8.80443	8.80443	0.0433062	0.750245	-1.3329	PF-00477736 down vs DMSO
15412	8008922	PPM1D	NM_003620	0.0029826	8.74359	8.74359	0.0029826	1.60182	1.60182	PF-00477736 up vs DMSO
19632	8051119	PPM1G	NM_177983	0.0033195	8.78979	8.78979	0.0033195	0.712699	-1.40312	PF-00477736 down vs DMSO
11187	7966368	PPP1CC	NM_002710	0.0373716	11.603	11.603	0.0373716	0.866367	-1.15425	PF-00477736 down vs DMSO
9499	7949060	PPP1R14B	NM_138689	0.0432644	10.3287	10.3287	0.0432644	0.768651	-1.30098	PF-00477736 down vs DMSO
17483	8030128	PPP1R15A	NM_014330	0.001569	7.35141	7.35141	0.001569	2.88355	2.88355	PF-00477736 up vs DMSO
26042	8114900	PPP2R2B	NM_004576	0.00228688	4.39569	4.39569	0.00228688	1.14543	1.14543	PF-00477736 up vs DMSO
31493	8166455	PRDX4	NM_006406	0.0205684	7.86614	7.86614	0.0205684	0.672361	-1.4873	PF-00477736 down vs DMSO
7275	7927606	PRKG1	NM_001098512	0.0469027	3.52437	3.52437	0.0469027	1.15258	1.15258	PF-00477736 up vs DMSO
20226	8057034	PRKRA	NM_003690	0.0236787	9.17842	9.17842	0.0236787	0.845731	-1.18241	PF-00477736 down vs DMSO
27029	8124185	PRL	NM_000948	0.0235902	3.42699	3.42699	0.0235902	1.18547	1.18547	PF-00477736 up vs DMSO
17513	8030437	PRMT1	NR_033397	0.028879	9.76888	9.76888	0.028879	0.795631	-1.25686	PF-00477736 down vs DMSO
23527	8091043	PRR23C	NM_001134657	0.0109917	3.71988	3.71988	0.0109917	0.907519	-1.10191	PF-00477736 down vs DMSO
31040	8161884	PRUNE2	NM_015225	0.0296182	3.43662	3.43662	0.0296182	1.10895	1.10895	PF-00477736 up vs DMSO
28585	8139232	PSMA2	NM_002787	0.0112423	10.845	10.845	0.0112423	0.793628	-1.26004	PF-00477736 down vs DMSO
15199	8006812	PSMB3	NM_002795	0.00866841	10.4724	10.4724	0.00866841	0.748366	-1.33624	PF-00477736 down vs DMSO
31251	8164067	PSMB7	NM_002799	0.0191009	10.5423	10.5423	0.0191009	0.794285	-1.25899	PF-00477736 down vs DMSO
12011	7974380	PSMC6	NM_002806	0.0215385	8.45302	8.45302	0.0215385	0.781932	-1.27888	PF-00477736 down vs DMSO
19446	8049016	PSMD1	NM_002807	0.00271371	9.81327	9.81327	0.00271371	0.751528	-1.33062	PF-00477736 down vs DMSO
19161	8045946	PSMD14	NM_005805	0.0244715	10.0759	10.0759	0.0244715	0.732222	-1.36571	PF-00477736 down vs DMSO
22914	8084423	PSMD2	NM_002808	0.0216597	9.49475	9.49475	0.0216597	0.811531	-1.23224	PF-00477736 down vs DMSO
28632	8139737	PSPH	NM_004577	0.0255246	8.20748	8.20748	0.0255246	1.38273	1.38273	PF-00477736 up vs DMSO
30766	8159521	PTGDS	NM_000954	0.0124138	5.26812	5.26812	0.0124138	1.14534	1.14534	PF-00477736 up vs DMSO
10972	7964250	PTGES3	NM_006601	0.00985497	10.9388	10.9388	0.00985497	0.750036	-1.33327	PF-00477736 down vs DMSO
20573	8061136	PTMA	NM_002823	0.0494953	13.6458	13.6458	0.0494953	0.925139	-1.08092	PF-00477736 down vs DMSO
28890	8142061	PUS7	NM_019042	0.0420048	8.28867	8.28867	0.0420048	0.632552	-1.5809	PF-00477736 down vs DMSO
18591	8040618	RAB10	NM_016131	0.00822271	10.5835	10.5835	0.00822271	0.830079	-1.2047	PF-00477736 down vs DMSO
17994	8035398	RAB3A	NM_002866	0.0320257	7.72099	7.72099	0.0320257	0.665564	-1.50248	PF-00477736 down vs DMSO
30633	8157843	RABEPK	NM_005833	0.0176482	7.62458	7.62458	0.0176482	0.7088	-1.41083	PF-00477736 down vs DMSO
15564	8010737	RAC3	NM_005052	0.0482917	8.00024	8.00024	0.0482917	0.821417	-1.21741	PF-00477736 down vs DMSO
25664	8111457	RAD1	NM_002853	0.00330288	8.31202	8.31202	0.00330288	0.785505	-1.27307	PF-00477736 down vs DMSO
22998	8085145	RAD18	NM_020165	0.0114965	9.60853	9.60853	0.0114965	0.777246	-1.28659	PF-00477736 down vs DMSO
30556	8157125	RAD23B	NM_002874	0.000300089	9.05617	9.05617	0.000300089	0.76426	-1.30846	PF-00477736 down vs DMSO
12922	7982792	RAD51	NM_002875	0.0259886	8.62549	8.62549	0.0259886	0.734394	-1.36167	PF-00477736 down vs DMSO

10593	7960261	RAD52	NM_134424	0.0074012	7.39086	7.39086	0.0074012	1.21879	1.21879	PF-00477736 up vs DMSO
27841	8132406	RALA	NM_005402	0.0361209	8.34796	8.34796	0.0361209	0.852899	-1.17247	PF-00477736 down vs DMSO
26400	8117888	RAN	NM_006325	0.0231902	10.984	10.984	0.0231902	0.793635	-1.26002	PF-00477736 down vs DMSO
10564	7959882	RAN	NM_006325	0.0250425	9.4482	9.4482	0.0250425	0.834599	-1.19818	PF-00477736 down vs DMSO
17963	8035050	RASAL3	NM_022904	0.0464664	7.14872	7.14872	0.0464664	1.51836	1.51836	PF-00477736 up vs DMSO
32099	8171516	RBBP7	NM_002893	0.0170966	11.1998	11.1998	0.0170966	0.79447	-1.2587	PF-00477736 down vs DMSO
16337	8018993	RBFOX3	NM_001082575	0.004147	5.44239	5.44239	0.004147	1.2066	1.2066	PF-00477736 up vs DMSO
8689	7941694	RBM14	NM_006328	0.00836977	9.63274	9.63274	0.00836977	0.742639	-1.34655	PF-00477736 down vs DMSO
21064	8066009	RBM39	NM_184234	0.00809499	10.4487	10.4487	0.00809499	1.22125	1.22125	PF-00477736 up vs DMSO
22468	8079869	RBM5	NM_005778	0.0357856	9.27959	9.27959	0.0357856	1.38305	1.38305	PF-00477736 up vs DMSO
4490	7899462	RCC1	NM_001048194	0.039227	8.83095	8.83095	0.039227	0.783476	-1.27636	PF-00477736 down vs DMSO
5472	7909529	RCOR3	NM_001136223	0.0213371	8.56804	8.56804	0.0213371	1.34275	1.34275	PF-00477736 up vs DMSO
17426	8029580	RELB	NM_006509	0.00170761	6.36471	6.36471	0.00170761	1.97129	1.97129	PF-00477736 up vs DMSO
23698	8092640	RFC4	NM_002916	0.0132262	10.7451	10.7451	0.0132262	0.711383	-1.40571	PF-00477736 down vs DMSO
30799	8159876	RFX3	NM_134428	0.00843392	6.12159	6.12159	0.00843392	1.68131	1.68131	PF-00477736 up vs DMSO
6784	7922717	RGS16	NM_002928	0.0178947	7.14094	7.14094	0.0178947	2.39957	2.39957	PF-00477736 up vs DMSO
5370	7908382	RGS21	NM_001039152	0.0325786	3.50302	3.50302	0.0325786	1.13354	1.13354	PF-00477736 up vs DMSO
31639	8167774	RIBC1	NM_001031745	0.0138687	5.39747	5.39747	0.0138687	1.12775	1.12775	PF-00477736 up vs DMSO
32320	8173615	RLIM	NM_183353	0.0119287	8.125	8.125	0.0119287	1.14427	1.14427	PF-00477736 up vs DMSO
10848	7962884	RND1	NM_014470	0.000675423	5.35555	5.35555	0.000675423	2.09633	2.09633	PF-00477736 up vs DMSO
5971	7914603	RNF19B	NM_153341	0.0361971	7.29126	7.29126	0.0361971	1.33039	1.33039	PF-00477736 up vs DMSO
10517	7959282	RNF34	NM_194271	0.0409589	8.58241	8.58241	0.0409589	0.844903	-1.18357	PF-00477736 down vs DMSO
9067	7945420	RNH1	NM_002939	0.0276793	7.73528	7.73528	0.0276793	0.824293	-1.21316	PF-00477736 down vs DMSO
14396	7998774	RNPS1	NM_080594	0.0235362	10.6163	10.6163	0.0235362	0.859505	-1.16346	PF-00477736 down vs DMSO
4495	7899502	RNU11	NR_004407	0.0010638	7.99925	7.99925	0.0010638	2.28336	2.28336	PF-00477736 up vs DMSO
4369	7898375	RNU1-1	NR_004430	0.0443288	11.4636	11.4636	0.0443288	1.36542	1.36542	PF-00477736 up vs DMSO
4373	7898411	RNU1-1	NR_004430	0.0443288	11.4636	11.4636	0.0443288	1.36542	1.36542	PF-00477736 up vs DMSO
5819	7912800	RNU1-1	NR_004430	0.0443288	11.4636	11.4636	0.0443288	1.36542	1.36542	PF-00477736 up vs DMSO
5825	7912850	RNU1-1	NR_004430	0.0443288	11.4636	11.4636	0.0443288	1.36542	1.36542	PF-00477736 up vs DMSO
6460	7919576	RNU1-1	NR_004430	0.0443288	11.4636	11.4636	0.0443288	1.36542	1.36542	PF-00477736 up vs DMSO
25726	8112176	RNU6ATAC	NR_023344	0.0405705	5.50118	5.50118	0.0405705	1.5261	1.5261	PF-00477736 up vs DMSO
16437	8019804	ROCK1P1	NR_033770	0.0118197	3.58635	3.58635	0.0118197	2.46798	2.46798	PF-00477736 up vs DMSO
31190	8163402	ROD1	NM_005156	0.0294009	10.0008	10.0008	0.0294009	0.788182	-1.26874	PF-00477736 down vs DMSO
14878	8003679	RPA1	NM_002945	0.00953255	9.23145	9.23145	0.00953255	0.814679	-1.22748	PF-00477736 down vs DMSO
18861	8043413	RPIA	NM_144563	0.0400718	7.68843	7.68843	0.0400718	0.755444	-1.32372	PF-00477736 down vs DMSO
7431	7929116	RPP30	NM_006413	0.0123279	9.55868	9.55868	0.0123279	0.745564	-1.34127	PF-00477736 down vs DMSO
26971	8123717	RPP40	NM_006638	0.0339347	7.46833	7.46833	0.0339347	0.68016	-1.47024	PF-00477736 down vs DMSO
8633	7941039	RPS6KA4	NM_003942	0.0204696	6.90176	6.90176	0.0204696	0.806033	-1.24064	PF-00477736 down vs DMSO
8245	7937915	RRM1	NM_001033	0.0483809	10.383	10.383	0.0483809	0.742134	-1.34746	PF-00477736 down vs DMSO
11260	7967304	RSRC2	NR_036435	0.0196443	8.84636	8.84636	0.0196443	1.38628	1.38628	PF-00477736 up vs DMSO
17919	8034555	RTBDN	NM_031429	0.0348364	4.70924	4.70924	0.0348364	1.14073	1.14073	PF-00477736 up vs DMSO
17482	8030113	RUVBL2	NM_006666	0.0352939	9.01804	9.01804	0.0352939	0.798148	-1.2529	PF-00477736 down vs DMSO
21861	8073645	SAMM50	NM_015380	0.0454292	9.58757	9.58757	0.0454292	0.813819	-1.22877	PF-00477736 down vs DMSO
4890	7903619	SARS	NM_006513	0.0266173	8.90923	8.90923	0.0266173	1.49081	1.49081	PF-00477736 up vs DMSO
17718	8032157	SBNO2	NM_014963	0.039315	7.28889	7.28889	0.039315	1.12176	1.12176	PF-00477736 up vs DMSO
24284	8098195	SC4MOL	NM_006745	0.0152398	9.29294	9.29294	0.0152398	0.612913	-1.63155	PF-00477736 down vs DMSO
24513	8100347	SCFD2	NM_152540	0.0174127	8.52363	8.52363	0.0174127	0.754404	-1.32555	PF-00477736 down vs DMSO
18070	8036033	SCGBL	NM_001025591	0.0304153	5.35052	5.35052	0.0304153	1.25239	1.25239	PF-00477736 up vs DMSO
5611	7911017	SDCCAG8	NM_006642	0.0365417	6.93629	6.93629	0.0365417	1.32642	1.32642	PF-00477736 up vs DMSO
16488	8020254	SEH1L	NM_031216	0.0316869	9.30556	9.30556	0.0316869	0.679603	-1.47145	PF-00477736 down vs DMSO
24442	8099696	SEPSECS	NM_016955	0.031124	7.69651	7.69651	0.031124	1.24673	1.24673	PF-00477736 up vs DMSO
28131	8135069	SERPINE1	NM_000602	0.000800523	5.03659	5.03659	0.000800523	1.53491	1.53491	PF-00477736 up vs DMSO
18164	8036902	SERTAD1	NM_013376	0.0344584	6.08116	6.08116	0.0344584	1.36909	1.36909	PF-00477736 up vs DMSO
4487	7899436	SESN2	NM_031459	0.00107603	6.74837	6.74837	0.00107603	2.22089	2.22089	PF-00477736 up vs DMSO
30671	8158372	SET	NM_003011	0.00917361	11.8903	11.8903	0.00917361	0.861777	-1.16039	PF-00477736 down vs DMSO
6264	7917674	SET	NM_001122821	0.0409003	10.3081	10.3081	0.0409003	0.870549	-1.1487	PF-00477736 down vs DMSO
5067	7905258	SETDB1	NM_001145415	0.0150712	8.21134	8.21134	0.0150712	1.21873	1.21873	PF-00477736 up vs DMSO
25526	8110090	SFXN1	NM_022754	0.0326158	10.0725	10.0725	0.0326158	0.715073	-1.39846	PF-00477736 down vs DMSO
7521	7930148	SFXN2	NM_178858	0.00894188	8.4137	8.4137	0.00894188	0.710585	-1.40729	PF-00477736 down vs DMSO
8126	7936641	SFXN4	NM_213649	0.0384193	8.26493	8.26493	0.0384193	0.740222	-1.35095	PF-00477736 down vs DMSO
5860	7913290	SH2D5	NM_001103161	0.0358172	5.23201	5.23201	0.0358172	1.16669	1.16669	PF-00477736 up vs DMSO
25023	8104912	SKP2	NM_005983	0.000101712	10.5513	10.5513	0.000101712	0.632441	-1.58117	PF-00477736 down vs DMSO
24363	8098904	SLBP	NM_006527	0.0214633	9.68393	9.68393	0.0214633	0.795721	-1.25672	PF-00477736 down vs DMSO
21578	8070912	SLC19A1	NM_194255	0.0266231	8.68246	8.68246	0.0266231	0.738117	-1.3548	PF-00477736 down vs DMSO
21931	8074388	SLC25A1	NM_005984	0.0208712	9.3373	9.3373	0.0208712	0.700181	-1.4282	PF-00477736 down vs DMSO
15559	8010673	SLC25A10	NM_012140	0.017931	8.2514	8.2514	0.017931	0.639042	-1.56484	PF-00477736 down vs DMSO
22142	8076260	SLC25A17	NM_006358	0.00600811	8.26954	8.26954	0.00600811	0.741393	-1.34881	PF-00477736 down vs DMSO
4285	7897460	SLC25A33	NM_032315	0.0157022	8.21112	8.21112	0.0157022	0.780977	-1.28045	PF-00477736 down vs DMSO
9360	7948088	SLC25A33	NM_032315	0.0170567	8.00906	8.00906	0.0170567	0.747499	-1.3378	PF-00477736 down vs DMSO
16069	8016018	SLC25A39	NM_001143780	0.0229806	9.82291	9.82291	0.0229806	0.711317	-1.40584	PF-00477736 down vs DMSO
12976	7983447	SLC28A2	NM_004212	0.0200609	3.863	3.863	0.0200609	1.09895	1.09895	PF-00477736 up vs DMSO
31058	8162059	SLC28A3	NM_022127	0.0159171	4.36776	4.36776	0.0159171	1.15508	1.15508	PF-00477736 up vs DMSO
6055	7915472	SLC2A1	NM_006516	0.0123534	9.33711	9.33711	0.0123534	0.589354	-1.69677	PF-00477736 down vs DMSO
14940	8004309	SLC2A4	NM_001042	0.00649907	6.4051	6.4051	0.00649907	0.80062	-1.24903	PF-00477736 down vs DMSO
25546	8110347	SLC34A1	NM_003052	0.0246025	5.82437	5.82437	0.0246025	0.874295	-1.14378	PF-00477736 down vs DMSO
16769	8022927	SLC39A6	NM_012319	0.0278681	8.70483	8.70483	0.0278681	0.824985	-1.21214	PF-00477736 down vs DMSO
19625	8051030	SLC5A6	NM_021095	0.0191948	8.90824	8.90824	0.0191948	0.694121	-1.44067	PF-00477736 down vs DMSO
31486	8166402	SMS	NM_004595	0.022991	9.23387	9.23387	0.022991	0.750885	-1.33176	PF-00477736 down vs DMSO
20786	8063382	SNAI1	NM_005985	0.031948	5.71296	5.71296	0.031948	1.63707	1.63707	PF-00477736 up vs DMSO
11665	7971386	SNORA31	NR_002967	0.0368307	5.84855	5.84855	0.0368307	1.30354	1.30354	PF-00477736 up vs DMSO
9697	7951038	SNORA40	NR_002973	0.0478641	8.34924	8.34924	0.0478641	0.744744	-1.34274	PF-00477736 down vs DMSO
8319	7938293	SNORA45	NR_002977	0.0200017	7.61702	7.61702	0.0200017	0.626855	-1.59527	PF-00477736 down vs DMSO
15436	8009241	SNORD104	NR_004380	0.0241525	9.81008	9.81008	0.0241525	1.32961	1.32961	PF-00477736 up vs DMSO
12828	7982068	SNORD115-31	NR_003346	0.0174698	2.48929	2.48929	0.0174698	1.17832	1.17832	PF-00477736 up vs DMSO
15113	8005957	SNORD4B	NR_000009	0.0295539	8.11889	8.11889	0.0295539	0.692657	-1.44372	PF-00477736 down vs DMSO
22135	8076219	SNORD83B	NR_000028	0.0165291	7.12647	7.12647	0.0165291	0.769331	-1.29983	PF-00477736 down vs DMSO
26202	8116532	SNORD95	NR_002591	0.0410009	9.04027	9.04027	0.0410009	1.39996	1.39996	PF-00477736 up vs DMSO
13744	7991735	SNRNP25	NM_024571	0.0312121	9.95229	9.95229	0.0312121	0.73127	-1.36748	PF-00477736 down vs DMSO
17359	8028916	SNRPA	NM_004596	0.0345541	10.6928	10.6928	0.0345541	0.814855	-1.22721	PF-00477736 down vs DMSO
16504	8020411	SNRPD1	NM_006938	0.0203439	8.81902	8.81902	0.0203439	0.762635	-1.31124	PF-00477736 down vs DMSO
10379	7957649	SNRPF	NM_003095	0.00530557	8.12714	8.12714	0.00530557	0.719798	-1.38928	PF-00477736 down vs DMSO
29963	8151572	SNX16	NM_022133	0.012503	6.48078	6.48078	0.012503	1.6578	1.6578	PF-00477736 up vs DMSO
23450	8090256	SNX4	NM_003794	0.0315041	7.83834	7.83834	0.0315041	0.867527	-1.1527	PF-00477736 down vs DMSO
14466	7999423	SOCS1	NM_003745	0.000802112	6.18193	6.18193	0.000802112	1.55325	1.55325	PF-00477736 up vs DMSO
21300	8068168	SOD1	NM_000454	0.0306464	10.4878	10.4878	0.0306464	0.728003	-1.37362	PF-00477736 down vs DMSO
26102	8115516	SOX30	NM_178424	0.00441936	4.18693	4.18693	0.00441936	1.24054	1.24054	PF-00477736 up vs DMSO
20199	8056798	SP3	NM_003111	0.0238994	8.8435	8.8435	0.0238994	1.09515	1.09515	PF-00477736 up vs DMSO
29658	8149180	SPAG11B	NM_016512	0.0346053	3.56033	3.56033	0.0346053	1.15196	1.15196	PF-00477736 up vs DMSO
17877	8034122	SPC24	NM_182513	0.0318432	8.37083	8.37083	0.0318432	0.719149	-1.39053	PF-00477736 down vs DMSO
13538	7989696	SPG21	NM_001127889	0.0489817	9.9561	9.9561	0.0489817	0.741905	-1.34788	PF-00477736 down vs DMSO
5769	7912374	SRM	NM_003132	0.025158	11.0095	11.0095	0.025158	0.672689	-1.48657	PF-00477736 down vs DMSO
23956	8095230	SRP72	NM_006947	0.0274184	11.2714	11.2714	0.0274184	0.836011	-1.19616	PF-00477736 down vs DMSO
16169	8016898	SRSF1	NM_001078166	0.018306	11.4563	11.4563	0.018306	0.845074	-1.18333	PF-00477736 down vs DMSO
9394	7948192	SSRP1	NM_003146	0.0321573	9.79151	9.79151	0.0321573	0.818977	-1.22103	PF-00477736 down vs DMSO
27962	8133215	STAG3L4	NM_022906	0.0194874	7.20918	7.20918	0.0194874	1.46845	1.46845	PF-00477736 up vs DMSO
11961	7973962	STELLAR	BC062480	0.00446459	4.77838	4.77838	0.00446459	0.780827	-1.28069	PF-00477736 down vs DMSO
33004	8179391	STK19	NR_026717	0.00519161	6.87964	6.87964	0.00519161	1.35094	1.35094	PF-00477736 up vs DMSO
26448	8118395	STK19	NR_026717	0.00588287	6.75381	6.75381	0.00588287	1.37607	1.37607	PF-00477736 up vs DMSO
32871	8178164	STK19	NR_026717	0.00588287	6.75381	6.75381	0.00588287	1.37607	1.37607	PF-00477736 up vs DMSO
16372	8019350	STRA13	NM_144998	0.018636	8.78942	8.78942	0.018636	0.751126	-1.33133	PF-00477736 down vs DMSO
13773	7992021	STUB1	NM_005861	0.0387858	7.51926	7.51926	0.0387858	0.817518	-1.22321	PF-00477736 down vs DMSO
9470	7948864	STX5	NM_003164	0.0243841	7.96674	7.96674	0.0243841	1.38989	1.38989	PF-00477736 up vs DMSO
18017	8035666	SUGP1	NM_172231	0.00993119	7.31762	7.31762	0.00993119	1.15815	1.15815	PF-00477736 up vs DMSO
5212	7906574	SUMO1P3	NR_002190	0.0156233	3.63765	3.63765	0.0156233	0.80719	-1.23887	PF-00477736 down vs DMSO
12339	7977674	SUPT16H	NM_007192	0.0429367	10.5605	10.5605	0.0429367	0.761376	-1.31341	PF-00477736 down vs DMSO
9578	7949931	SUV420H1	NM_017635	0.00924305	8.18931	8.18931	0.00924305	1.37024	1.37024	PF-00477736 up vs DMSO
7598	7931031	TACC2	NM_206862	0.0183085	5.21068	5.21068	0.0183085	0.898511	-1.11295	PF-00477736 down vs DMSO
6702	7922008	TADA1	NM_053053	0.0267323	8.01345	8.01345	0.0267323	0.776703	-1.28749	PF-00477736 down vs DMSO
32817	8177635	TAF9	NM_003187	0.00571714	8.36498	8.36498	0.00571714	0.816816	-1.22427	PF-00477736 down vs DMSO
25762	8112458	TAF9	NM_003187	0.0105576	8.48193	8.48193	0.0105576	0.817378	-1.22342	PF-00477736 down vs DMSO
8203	7937465	TALDO1	NM_006755	0.0115541	10.0293	10.0293	0.0115541	0.763505	-1.30975	PF-00477736 down vs DMSO
4298	7897663	TARDBP	NM_007375	0.0207868	10.2331	10.2331	0.0207868	0.877669	-1.13938	PF-00477736 down vs DMSO
19162	8045962	TBR1	NM_006593	0.0466914	4.99613	4.99613	0.0466914	1.15459	1.15459	PF-00477736 up vs DMSO
28553	8138997	TBX20	NM_001077653	0.0459556	4.80952	4.80952	0.0459556	0.91192	-1.09659	PF-00477736 down vs DMSO
25437	8109254	TCOF1	NM_001135243	0.0188405	8.4952	8.4952	0.0188405	0.790183	-1.26553	PF-00477736 down vs DMSO
10427	7958262	TCP11L2	NM_152772	0.0279902	5.4044	5.4044	0.0279902	2.20886	2.20886	PF-00477736 up vs DMSO
22460	8079746	TCTA	NM_022171	0.0494936	7.3468	7.3468	0.0494936	1.29185	1.29185	PF-00477736 up vs DMSO
17103	8026341	TECR	NM_138501	0.0464156	10.8101	10.8101	0.0464156	0.787647	-1.2696	PF-00477736 down vs DMSO
7551	7930487	TECTB	NM_058222	0.0448879	3.32997	3.32997	0.0448879	1.09294	1.09294	PF-00477736 up vs DMSO
32205	8172520	TFE3	NM_006521	0.047785	7.12383	7.12383	0.047785	1.2922	1.2922	PF-00477736 up vs DMSO
23738	8093053	TFRC	NM_003234	0.0164356	10.8488	10.8488	0.0164356	0.50992	-1.96109	PF-00477736 down vs DMSO
19854	8053388	TGOLN2	NM_006464	0.00296829	9.00168	9.00168	0.00296829	1.25762	1.25762	PF-00477736 up vs DMSO
24058	8096091	THAP9	NM_024672	0.0305676	6.49718	6.49718	0.0305676	1.42027	1.42027	PF-00477736 up vs DMSO
16432	8019772	THOC4	NM_005782	0.038555	11.3086	11.3086	0.038555	0.815149	-1.22677	PF-00477736 down vs DMSO
16364	8019273	THOC4	NM_005782	0.0419827	11.2574	11.2574	0.0419827	0.82285	-1.21529	PF-00477736 down vs DMSO
15215	8007008	THRA	NM_003250	0.0139039	8.04582	8.04582	0.0139039	0.664053	-1.5059	PF-00477736 down vs DMSO
11711	7971800	THSD1P1	NR_002816	0.017856	5.91753	5.91753	0.017856	1.33698	1.33698	PF-00477736 up vs DMSO
13086	7984588	THSD4	NM_024817	0.0214155	5.30735	5.30735	0.0214155	1.12381	1.12381	PF-00477736 up vs DMSO
14702	8001818	TK2	NM_004614	0.00866021	6.34261	6.34261	0.00866021	1.36645	1.36645	PF-00477736 up vs DMSO

15424	8009014	TLK2	NM_006852	0.0346856	8.9425	8.9425	0.0346856	1.22928	1.22928	PF-00477736 up vs DMSO
20409	8059525	TM4SF20	NM_024795	0.019063	3.04434	3.04434	0.019063	0.916535	-1.09107	PF-00477736 down vs DMSO
31386	8165642	TMEM203	NM_053045	0.0116072	7.70746	7.70746	0.0116072	0.740472	-1.35049	PF-00477736 down vs DMSO
31372	8165486	TMEM203	NM_053045	0.027989	8.08455	8.08455	0.027989	0.781748	-1.27919	PF-00477736 down vs DMSO
23709	8092735	TMEM207	NM_207316	0.0181015	4.15392	4.15392	0.0181015	0.887043	-1.12734	PF-00477736 down vs DMSO
15101	8005839	TMEM97	NM_014573	0.00581345	10.4777	10.4777	0.00581345	0.589701	-1.69577	PF-00477736 down vs DMSO
5741	7912145	TNFRSF9	NM_001561	0.0265882	4.68481	4.68481	0.0265882	1.98286	1.98286	PF-00477736 up vs DMSO
16985	8025053	TNFSF9	NM_003811	0.000225687	6.68635	6.68635	0.000225687	1.53568	1.53568	PF-00477736 up vs DMSO
17419	8029521	TOMM40	NM_001128917	0.00551855	8.52697	8.52697	0.00551855	0.750246	-1.3329	PF-00477736 down vs DMSO
6774	7922648	TOR1AIP1	NM_015602	0.00500568	5.79797	5.79797	0.00500568	1.25361	1.25361	PF-00477736 up vs DMSO
20662	8062034	TP53INP2	NM_021202	0.0255741	6.84929	6.84929	0.0255741	1.19	1.19	PF-00477736 up vs DMSO
11879	7973142	TPPP2	NM_173846	0.0461059	4.09127	4.09127	0.0461059	1.10027	1.10027	PF-00477736 up vs DMSO
14428	7999025	TRAP1	NM_016292	0.0423382	9.98413	9.98413	0.0423382	0.717269	-1.39418	PF-00477736 down vs DMSO
12441	7978739	TRAPPC6B	NM_001079537	0.00258983	7.88792	7.88792	0.00258983	1.37353	1.37353	PF-00477736 up vs DMSO
20494	8060344	TRIB3	NM_021158	0.0395005	7.10263	7.10263	0.0395005	2.71894	2.71894	PF-00477736 up vs DMSO
24862	8103520	TRIM61	NM_001012414	0.0244923	5.77253	5.77253	0.0244923	0.775594	-1.28933	PF-00477736 down vs DMSO
9201	7946380	TRIM66	NM_014818	0.0169067	6.77268	6.77268	0.0169067	1.54915	1.54915	PF-00477736 up vs DMSO
24946	8104234	TRIP13	NM_004237	0.0143901	9.05365	9.05365	0.0143901	0.614919	-1.62623	PF-00477736 down vs DMSO
31284	8164428	TRUB2	NM_015679	0.0493443	8.7866	8.7866	0.0493443	0.807615	-1.23821	PF-00477736 down vs DMSO
32405	8174361	TSC22D3	NM_198057	0.0427995	7.30521	7.30521	0.0427995	1.96846	1.96846	PF-00477736 up vs DMSO
17593	8031145	TSEN34	NM_024075	0.0331743	6.93687	6.93687	0.0331743	0.892766	-1.12011	PF-00477736 down vs DMSO
13407	7987960	TTBK2	NM_173500	0.0486521	7.3726	7.3726	0.0486521	1.19072	1.19072	PF-00477736 up vs DMSO
22184	8076569	TTLL12	NM_015140	0.0232666	9.22382	9.22382	0.0232666	0.682662	-1.46485	PF-00477736 down vs DMSO
10860	7963046	TUBA1B	NM_006082	0.0359587	10.4724	10.4724	0.0359587	0.806483	-1.23995	PF-00477736 down vs DMSO
19084	8045291	TUBA3D	NM_080386	0.0370549	6.18766	6.18766	0.0370549	0.89788	-1.11374	PF-00477736 down vs DMSO
26408	8117995	TUBB	NM_178014	0.006915	12.9872	12.9872	0.006915	0.788404	-1.26839	PF-00477736 down vs DMSO
32843	8177858	TUBB	NM_178014	0.006915	12.9872	12.9872	0.006915	0.788404	-1.26839	PF-00477736 down vs DMSO
32980	8179174	TUBB	NM_178014	0.006915	12.9872	12.9872	0.006915	0.788404	-1.26839	PF-00477736 down vs DMSO
14565	8000603	TUFM	NM_003321	0.0268351	9.27054	9.27054	0.0268351	0.806947	-1.23924	PF-00477736 down vs DMSO
10817	7962441	TWF1	NM_002822	0.0367654	8.55431	8.55431	0.0367654	0.821289	-1.2176	PF-00477736 down vs DMSO
15854	8013906	TWF1	NM_002822	0.0417796	8.47119	8.47119	0.0417796	0.796491	-1.25551	PF-00477736 down vs DMSO
4990	7904726	TXNIP	NM_006472	0.0122023	9.7039	9.7039	0.0122023	1.78913	1.78913	PF-00477736 up vs DMSO
17866	8033996	TYK2	NM_003331	0.0316304	7.94818	7.94818	0.0316304	1.16551	1.16551	PF-00477736 up vs DMSO
15638	8011626	UBE2G1	NM_003342	0.00477585	10.9757	10.9757	0.00477585	0.811784	-1.23186	PF-00477736 down vs DMSO
11094	7965471	UBE2N	NM_003348	0.00178989	8.98642	8.98642	0.00178989	0.804169	-1.24352	PF-00477736 down vs DMSO
31961	8170298	UBE2NL	NM_001012989	0.000776593	7.03538	7.03538	0.000776593	0.776046	-1.28858	PF-00477736 down vs DMSO
18444	8039491	UBE2S	NM_014501	0.0383695	9.06553	9.06553	0.0383695	1.35605	1.35605	PF-00477736 up vs DMSO
32617	8176098	UBL4A	NM_014235	0.0159312	7.53357	7.53357	0.0159312	0.759082	-1.31738	PF-00477736 down vs DMSO
9462	7948782	UBXN1	NM_015853	0.0392155	8.40694	8.40694	0.0392155	1.11555	1.11555	PF-00477736 up vs DMSO
23161	8086981	UCN2	NM_033199	0.0363388	5.67955	5.67955	0.0363388	0.854316	-1.17053	PF-00477736 down vs DMSO
15504	8009995	UNK	NM_001080419	0.0105273	8.2134	8.2134	0.0105273	1.35908	1.35908	PF-00477736 up vs DMSO
21725	8072274	UQCR10	NM_001003684	0.0217666	9.10345	9.10345	0.0217666	0.809652	-1.2351	PF-00477736 down vs DMSO
17729	8032284	UQCR11	NM_006830	0.0126678	8.43548	8.43548	0.0126678	0.828371	-1.20719	PF-00477736 down vs DMSO
5807	7912670	UQCRH	NM_006004	0.0101459	9.74475	9.74475	0.0101459	0.731456	-1.36714	PF-00477736 down vs DMSO
25318	8107998	UQCRQ	NM_014402	0.0331903	8.94923	8.94923	0.0331903	0.871848	-1.14699	PF-00477736 down vs DMSO
5557	7910416	URB2	NM_014777	0.0150781	8.41235	8.41235	0.0150781	0.713605	-1.40134	PF-00477736 down vs DMSO
9956	7953483	USP5	NM_001098536	0.0240134	8.48794	8.48794	0.0240134	0.836761	-1.19508	PF-00477736 down vs DMSO
25143	8106193	UTP15	NM_032175	0.00734088	8.59591	8.59591	0.00734088	0.705832	-1.41677	PF-00477736 down vs DMSO
26412	8118028	VARS2	NM_020442	0.0378054	7.22245	7.22245	0.0378054	0.857722	-1.16588	PF-00477736 down vs DMSO
32846	8177901	VARS2	NM_020442	0.0451123	7.12574	7.12574	0.0451123	0.861245	-1.16111	PF-00477736 down vs DMSO
25961	8114145	VDAC1	NM_003374	0.0335679	9.28812	9.28812	0.0335679	0.80545	-1.24154	PF-00477736 down vs DMSO
7364	7928524	VDAC2	NM_001184783	0.00767413	7.03533	7.03533	0.00767413	0.756318	-1.3222	PF-00477736 down vs DMSO
8624	7940904	VEGFB	NM_003377	0.0164737	8.00708	8.00708	0.0164737	0.755523	-1.32359	PF-00477736 down vs DMSO
23595	8091678	VEPH1	NM_024621	0.0194428	3.17472	3.17472	0.0194428	1.14796	1.14796	PF-00477736 up vs DMSO
27947	8133114	VKORC1L1	NM_173517	0.00145061	9.1552	9.1552	0.00145061	0.753319	-1.32746	PF-00477736 down vs DMSO
32278	8173287	VSIG4	NM_001184830	0.0427073	4.23419	4.23419	0.0427073	0.873061	-1.1454	PF-00477736 down vs DMSO
17222	8027385	VSTM2B	NM_001146339	0.0235439	7.35518	7.35518	0.0235439	1.1149	1.1149	PF-00477736 up vs DMSO
7194	7926851	WAC	NR_024557	0.024604	10.6047	10.6047	0.024604	1.10205	1.10205	PF-00477736 up vs DMSO
12693	7981290	WARS	NM_004184	0.0375458	8.87918	8.87918	0.0375458	1.50727	1.50727	PF-00477736 up vs DMSO
12230	7976766	WDR25	NM_024515	0.0316261	6.30414	6.30414	0.0316261	1.09803	1.09803	PF-00477736 up vs DMSO
30736	8159111	WDR5	NM_017588	0.0442008	8.94538	8.94538	0.0442008	0.799937	-1.2501	PF-00477736 down vs DMSO
13624	7990700	WDR61	NM_025234	0.0469173	7.51163	7.51163	0.0469173	0.812908	-1.23015	PF-00477736 down vs DMSO
13674	7991126	WDR73	NM_032856	0.0275526	7.26707	7.26707	0.0275526	1.17095	1.17095	PF-00477736 up vs DMSO
12963	7983306	WDR76	NM_024908	0.039615	9.86182	9.86182	0.039615	0.754646	-1.32512	PF-00477736 down vs DMSO
6341	7918517	WDR77	NM_024102	0.0211926	9.15391	9.15391	0.0211926	0.811759	-1.23189	PF-00477736 down vs DMSO
29274	8145702	WRN	NM_000553	0.0143655	8.75185	8.75185	0.0143655	0.751016	-1.33153	PF-00477736 down vs DMSO
6713	7922127	XCL2	NM_003175	0.0497243	3.2088	3.2088	0.0497243	1.16236	1.16236	PF-00477736 up vs DMSO
27290	8126588	XPO5	NM_020750	0.0492039	9.62736	9.62736	0.0492039	0.731272	-1.36748	PF-00477736 down vs DMSO
14555	8000482	XPO6	NM_015171	0.0256284	10.6946	10.6946	0.0256284	0.806602	-1.23977	PF-00477736 down vs DMSO
29096	8144036	XRCC2	NM_005431	0.0103727	9.55509	9.55509	0.0103727	0.660289	-1.51449	PF-00477736 down vs DMSO
19371	8048146	XRCC5	NM_021141	0.0101629	11.0138	11.0138	0.0101629	0.799322	-1.25106	PF-00477736 down vs DMSO
21840	8073457	XRCC6	NM_001469	0.0291745	10.7148	10.7148	0.0291745	0.807955	-1.23769	PF-00477736 down vs DMSO
4599	7900585	YBX1	NM_004559	0.0182497	12.2413	12.2413	0.0182497	0.845423	-1.18284	PF-00477736 down vs DMSO
28892	8142084	YBX1P2	CR601484	0.0392259	10.5086	10.5086	0.0392259	0.770886	-1.29721	PF-00477736 down vs DMSO
18643	8041281	YIPF4	ENST00000238831	0.0322226	8.56014	8.56014	0.0322226	1.45225	1.45225	PF-00477736 up vs DMSO
20089	8055476	YWHAE	NM_006761	0.0070947	12.0997	12.0997	0.0070947	0.762255	-1.3119	PF-00477736 down vs DMSO
15592	8011011	YWHAE	NR_024058	0.0272184	12.1516	12.1516	0.0272184	0.796117	-1.2561	PF-00477736 down vs DMSO
28737	8140398	YWHAG	NM_012479	0.0425801	9.53226	9.53226	0.0425801	0.851992	-1.17372	PF-00477736 down vs DMSO
29429	8147040	ZBTB10	NM_001105539	0.0343572	7.51114	7.51114	0.0343572	1.47357	1.47357	PF-00477736 up vs DMSO
17769	8032755	ZBTB7A	NM_015898	0.0151479	8.09526	8.09526	0.0151479	0.847166	-1.18041	PF-00477736 down vs DMSO
10238	7956162	ZC3H10	NM_032786	0.000907154	5.81193	5.81193	0.000907154	1.21493	1.21493	PF-00477736 up vs DMSO
4556	7900146	ZC3H12A	NM_025079	0.0282218	6.61252	6.61252	0.0282218	1.17097	1.17097	PF-00477736 up vs DMSO
14476	7999496	ZC3H7A	NM_014153	0.0325517	8.72317	8.72317	0.0325517	1.31501	1.31501	PF-00477736 up vs DMSO
19399	8048478	ZFAND2B	NM_138802	0.0208474	7.84207	7.84207	0.0208474	1.21134	1.21134	PF-00477736 up vs DMSO
17340	8028652	ZFP36	NM_003407	0.0354811	7.12685	7.12685	0.0354811	1.37979	1.37979	PF-00477736 up vs DMSO
31382	8165622	ZMYND19	NM_138462	0.00155588	8.15648	8.15648	0.00155588	0.742542	-1.34673	PF-00477736 down vs DMSO
13846	7992854	ZNF213	NM_004220	0.0227034	6.07328	6.07328	0.0227034	1.08401	1.08401	PF-00477736 up vs DMSO
17408	8029399	ZNF226	NM_001032372	0.0316301	5.91912	5.91912	0.0316301	1.48495	1.48495	PF-00477736 up vs DMSO
18393	8039013	ZNF321	NM_203307	0.0329609	6.1849	6.1849	0.0329609	0.837659	-1.1938	PF-00477736 down vs DMSO
28821	8141305	ZNF394	NM_032164	0.0490942	8.27806	8.27806	0.0490942	1.24058	1.24058	PF-00477736 up vs DMSO
29060	8143702	ZNF425	NM_001001661	0.0182976	5.60911	5.60911	0.0182976	1.35736	1.35736	PF-00477736 up vs DMSO
17203	8027292	ZNF431	NM_133473	0.0313027	8.00017	8.00017	0.0313027	1.2868	1.2868	PF-00477736 up vs DMSO
17059	8025945	ZNF441	NM_152355	0.000857398	4.46228	4.46228	0.000857398	1.50175	1.50175	PF-00477736 up vs DMSO
25561	8110478	ZNF454	NM_001178089	0.0396012	4.6979	4.6979	0.0396012	1.1724	1.1724	PF-00477736 up vs DMSO
28652	8139800	ZNF479	NM_033273	0.0102143	3.44675	3.44675	0.0102143	1.08956	1.08956	PF-00477736 up vs DMSO
27925	8133023	ZNF479	NM_033273	0.0189269	3.77696	3.77696	0.0189269	1.1005	1.1005	PF-00477736 up vs DMSO
17060	8025951	ZNF491	NM_152356	0.0458268	4.6384	4.6384	0.0458268	1.22892	1.22892	PF-00477736 up vs DMSO
17853	8033795	ZNF561	NM_152289	0.042546	7.65977	7.65977	0.042546	1.29342	1.29342	PF-00477736 up vs DMSO
14614	8000974	ZNF668	NM_024706	0.00250658	6.57981	6.57981	0.00250658	0.848497	-1.17855	PF-00477736 down vs DMSO
31554	8166989	ZNF673	NM_001129898	0.0325395	7.80078	7.80078	0.0325395	1.24188	1.24188	PF-00477736 up vs DMSO
32178	8172274	ZNF674	NM_001039891	0.0350344	5.39853	5.39853	0.0350344	1.2905	1.2905	PF-00477736 up vs DMSO
5536	7910190	ZNF678	NR_033184	0.0287361	6.35976	6.35976	0.0287361	1.32589	1.32589	PF-00477736 up vs DMSO
18033	8035803	ZNF708	NM_021269	0.0247131	7.35236	7.35236	0.0247131	1.35747	1.35747	PF-00477736 up vs DMSO
14053	7995252	ZNF720	NM_001130913	0.0394599	8.02797	8.02797	0.0394599	1.4866	1.4866	PF-00477736 up vs DMSO
24350	8098758	ZNF721	NM_133474	0.0395463	8.81167	8.81167	0.0395463	1.24199	1.24199	PF-00477736 up vs DMSO
17073	8026007	ZNF791	NM_153358	0.0439364	7.9497	7.9497	0.0439364	1.28674	1.28674	PF-00477736 up vs DMSO
17574	8030997	ZNF813	NM_001004301	0.0350848	6.62696	6.62696	0.0350848	0.819558	-1.22017	PF-00477736 down vs DMSO
17671	8031799	ZSCAN4	NM_152677	0.00616157	3.45207	3.45207	0.00616157	1.31736	1.31736	PF-00477736 up vs DMSO
