# Supplementary material for: In vitro and in vivo single-agent efficacy of checkpoint kinase inhibition in acute lymphoblastic leukemia
Source: J Hematol Oncol. 2015 Nov 5;8:125. doi: 10.1186/s13045-015-0206-5 (PMC4635624; doi:10.1186/s13045-015-0206-5)
Supplement: Additional file 4: — Enrichment analysis report: enrichment by process networks. (PDF 35 kb) [file 13045_2015_206_MOESM4_ESM.pdf]

Enrichment analysis report

Enrichment by Process Networks

#	Networks	Total	pValue	Min FDR	p-value	FDR	In Data	Genes from Active Data
1	DNA damage_Checkpoint	124	6.86E-10	1.1E-07	6.86E-10	1.1E-07	26	RFC4, Ku70/80, PCNA, Ku80, 14-3-3 gamma, GADD45 alpha, Chk2, 14-3-3 epsilon, Ku70, Cyclin A, TLK2, GADD45 gamma, NF-kB, BTG2, CDK4, Cyclin A2, FANCD2, RUVBL2, ATF-3, MUS81, RAD1, Claspin, ANAPC5, c-Jun, CDK2, 14-3-3
2	Cell cycle_S phase	149	2.11E-09	1.69E-07	2.11E-09	1.69E-07	28	RFC4, POLE3-POLE4 complex, PCNA, MCM6, GADD45 alpha, Chk2, MCM3, Cyclin A, ChAF1 subunit B, PTMA, TLK2, RPA1, MCM2, POLD reg (p50), FEN1, Histone H3, POLD cat (p125), MCM5, HSP90, Cyclin A2, POLE3 (YBL1), Rad51, WRN, HP1, Histone H4, Histone H1, CDK2, CDC45L
3	Apoptosis_Apoptotic nucleus	159	3.97E-08	2.12E-06	3.97E-08	2.12E-06	27	PHAP1 (pp32), Ku70/80, Ku80, Tip60, GADD45 alpha, Chk2, ING4, Ku70, NF-kB2 (p100), GADD45 gamma, Tau (MAPT), HDAC3, Tubulin alpha, PIG8, NF-kB, Histone H3, Cullin 2, NDPK A, FANCD2, Tyk2, Rad51, C/EBPbeta, IEX1, SET, Histone H2B, c-Jun, Histone H1
4	DNA damage_DBS repair	116	1.87E-06	6.34E-05	1.87E-06	6.34E-05	20	Ku70/80, PCNA, Ku80, Tip60, Chk2, Ku70, PP2A regulatory, ChAF1 subunit B, XRCC2, E2N(UBC13), RPA1, Rad52, FEN1, Histone H3, FANCD2, RUVBL2, MUS81, Rad51, WRN, Histone H4
5	Transcription_Chromatin modification	128	2.38E-06	6.34E-05	2.38E-06	6.34E-05	21	Tip60, HMGI/Y, HAT1, CARM1, PRMT1, Histone H3.3, EPC1, TLK2, HDAC3, SETDB1, Histone H3, RUVBL2, ING3, RBBP7 (RbAp46), Histone H2, HP1 gamma, Histone H2A, Histone H2B, Histone H4, Pc2, Histone H1
6	Cell cycle_G1-S Interleukin regulation	128	2.38E-06	6.34E-05	2.38E-06	6.34E-05	21	c-Jun/c-Fos, Cyclin A, JunD/c-Fos, AP-1, NF-kB2 (p100), NF-kB, RelB (NF-kB subunit), c-Fos, JunB, CDK4, Cyclin A2, NF-kB2 (p52), RalA, IL-8, p19, JAK3, Elk-1, c-Jun/c-Jun, NF-kB p52/RelB, c-Jun, CDK2
7	Inflammation_MIF signaling	140	2.87E-06	6.55E-05	2.87E-06	6.55E-05	22	PHAP1 (pp32), CREB1, HMGI/Y, MIF, c-Jun/c-Fos, Cyclin A, NF-kB2 (p100), CDK5, SKP2, NF-kB, c-Fos, PLC-beta, PLA2, CDK4, ICAM1, NF-kB2 (p52), PLC-beta3, IL-8, cPLA2, NF-kB p52/RelB, c-Jun, CDK2
8	Cell cycle_G1-S Growth factor regulation	195	2.49E-05	0.000499	2.49E-05	0.000499	25	c-Jun/c-Fos, PP2A regulatory, Cyclin A, JunD/c-Fos, AP-1, NF-kB2 (p100), NF-kB, RelB (NF-kB subunit), c-Fos, JunB, CDK4, NDPK A, Cyclin A2, NF-kB2 (p52), NDPK complex, RalA, PPP2R2B, p19, c-Jun/c-Jun, NDPK B, RBM5, NF-kB p52/RelB, Betacellulin, c-Jun, CDK2
9	Cell cycle_G1-S	163	3.47E-05	0.000617	3.47E-05	0.000617	22	PCNA, 14-3-3 gamma, GADD45 alpha, Chk2, 14-3-3 epsilon, Cyclin A, Importin (karyopherin)-beta, Skp2/TrCP/FBXW, CDK5, SKP2, HDAC3, CDK4, Cyclin A2, FANCD2, Rad51, Claspin, p19, ANAPC5, SERTAD1, FosB, CDK2, 14-3-3
10	Protein folding_Response to unfolded proteins	69	4.64E-05	0.000742	4.64E-05	0.000742	13	HSP90 alpha, HSP10 (mitochondrial), UBC7, HSC70, IRE1, ERO1, HSP60, HSP90, CHIP, HSP70, HSPA4, DNAJB2, HERP
