# Supplementary material for: In vitro and in vivo single-agent efficacy of checkpoint kinase inhibition in acute lymphoblastic leukemia
Source: J Hematol Oncol. 2015 Nov 5;8:125. doi: 10.1186/s13045-015-0206-5 (PMC4635624; doi:10.1186/s13045-015-0206-5)
Supplement: Additional file 5: — Table S1. List of differentially expressed genes between treated cells and their untreated counterpart with a false discovery rate less than 0.05. Table S2. Main clinical and molecular characteristics of ALL patients whose primary ALL samples have been tested in vitro. Table S3. TP53 primer sequences. Table S4. Antibody reactivity and sources as well as the antigen retrieval protocols, dilutions, and revelation systems used in immunohistochemistry. (DOCX 28 kb) [file 13045_2015_206_MOESM5_ESM.docx]

**Additional file 5**

**Table S1.** List of differentially expressed genes between treated cells and their untreated counterpart with a false discovery rate less than 0.05.

| **Up-Regulated genes (PF-00477736 vs. DMSO)** | | | | |
| --- | --- | --- | --- | --- |
| Transcript ID | Gene Symbol | RefSeq | p-value (Treatment) | Fold-Change  (PF-00477736 vs. DMSO) |
| 7964460 | *DDIT3* | NM_001195053 | 6.06E-05 | 3.32494 |
| 7931810 | *KLF6* | NM_001300 | 8.41E-05 | 2.17448 |
| 7975779 | *FOS* | NM_005252 | 1.97E-04 | 2.40401 |
| 8124518 | *HIST1H2AJ* | NM_021066 | 0.000393 | 5.14566 |
| 7997740 | *MAP1LC3B* | NM_022818 | 0.000626 | 2.04974 |
| 7962884 | *RND1* | NM_014470 | 0.000675 | 2.09633 |
| 7909610 | *ATF3* | NM_001040619 | 0.000835 | 2.76388 |
| 7916609 | *JUN* | NM_002228 | 0.000932 | 5.3469 |
| 7899502 | *RNU11* | NR_004407 | 0.001064 | 2.28336 |
| 7899436 | *SESN2* | NM_031459 | 0.001076 | 2.22089 |
| 8063386 | *CEBPB* | NM_005194 | 0.0012 | 2.11244 |
| 8124484 | *HIST1H2BJ* | NM_021058 | 0.001519 | 2.65774 |
| 8113073 | *ARRDC3* | NM_020801 | 0.001555 | 2.17704 |
| 8030128 | *PPP1R15A* | NM_014330 | 0.001569 | 2.88355 |
| 8124540 | *HIST1H2AM* | NM_003514 | 0.001685 | 2.13115 |
| 7961075 | *CD69* | NM_001781 | 0.001693 | 3.16956 |
| 7919606 | *HIST2H2BF* | NM_001024599 | 0.001839 | 2.02203 |
| 7905067 | *HIST2H4A* | NM_003548 | 0.002048 | 3.53071 |
| 7919627 | *HIST2H4A* | NM_003548 | 0.002048 | 3.53071 |
| 7919584 | *HIST2H2BF* | NM_001024599 | 0.002878 | 2.18769 |
| 7940582 | *BEST1* | NM_004183 | 0.00341 | 2.3629 |
| 7930074 | *NFKB2* | NM_002502 | 0.006266 | 2.26153 |
| 8019804 | *ROCK1P1* | NR_033770 | 0.01182 | 2.46798 |
| 7922717 | *RGS16* | NM_002928 | 0.017895 | 2.39957 |
| 8124848 | *IER3* | NM_003897 | 0.018122 | 2.50753 |
| 8179704 | *IER3* | NM_003897 | 0.018122 | 2.50753 |
| 8081386 | *NFKBIZ* | NM_031419 | 0.018178 | 2.09757 |
| 7958262 | *TCP11L2* | NM_152772 | 0.02799 | 2.20886 |
| 8178435 | *IER3* | NM_003897 | 0.030662 | 2.41736 |
| 8108370 | *EGR1* | NM_001964 | 0.031673 | 2.57069 |
| 8095680 | *IL8* | NM_000584 | 0.036437 | 2.29928 |
| 8060344 | *TRIB3* | NM_021158 | 0.039501 | 2.71894 |
| 7902227 | *GADD45A* | NM_001924 | 0.044569 | 2.16306 |
| 7908917 | *BTG2* | NM_006763 | 0.04734 | 2.0536 |
| 7982868 | *CHAC1* | NM_024111 | 0.048647 | 2.10011 |
| **Down-Regulated genes (PF-00477736 vs. DMSO)** | | | | |
| 8013660 | ALDOC | NM_005165 | 0.0281531 | -2.18418 |
| 7937079 | BNIP3 | NM_004052 | 0.0356865 | -2.2734 |

**Table S2.** Main clinical and molecular characteristics of ALL patients whose primary ALL samples have been tested in vitro. Sensitivity was defined as very good for IC_50_ values at 24 hours ranging from 0.1 and 0.5 μM; good for IC_50_ values at 24 hours ranging from 0.5 and 1 μM and poor for IC_50_ values at 24 hours higher than 1 μM. NA: not available.

|  | **ID** | **Age** | **Sex** | **Blasts (%)** | **Cytogenetics** | **Molecular feature** | **Previous**  **therapy** | **Sensitivity** |
| --- | --- | --- | --- | --- | --- | --- | --- | --- |
| ***BCR-ABL1-***  **positive ALL** | 1 | 70 | M | 100 | 45XY,-7, t(9;22)(q34;q11) | Bcr-Abl p210 | NO | Very good |
|  | 2 | 74 | M | 100 | 45XY, -3, der(9)t(3;9)(q10;p10) | Bcr-Abl p190 | NO | Good |
|  | 3 | 69 | F | 100 | 46 XX, t(9;22)(q34;q11) | Bcr-Abl p190 | YES | Poor |
|  | 4 | 46 | M | 95 | 45XY,-7, t(9;22)(q34;q11), der (12), t(7;12)(q11p11) | Bcr-Abl p190 | YES | Very good |
|  | 5 | 53 | F | 100 | 49XX, +5, +8, t(9;22)(q34;p11) | Bcr-Abl p190 | YES | Very good |
|  | 6 | 34 | F | 80 | 45XX, t(9;22)(q34;q11), der(16)dic(16;20) (q13;11), -20 | Bcr-Abl p190 | NO | Good |
|  | 7 | 25 | M | 60 | NA | Bcr-Abl p190 | YES | Low |
|  | 12 | 59 | F | 100 | 46XX (12), 46 XX, t(9;22)(q34;q11) (13), 46XX del(9)(p13;p22), t(9;22)(q34;q11) (5) | Bcr-Abl p210 | NO | Good |
|  | 13 | 32 | M | 90 | 46 XY, t(9;22)(q34;q11) | Bcr-Abl p210 | NO | Low |
|  | 14 | 46 | F | 90 | NA | Bcr-Abl p210 | NO | Good |
| ***BCR-ABL1*-**  **negative ALL** | 8 | 43 | F | 100 | 46 XX der(19) t(1;19)(q23;p13) | E2A-PBX | NO | Very good |
|  | 9 | 42 | F | 90 | 46XX, t(4;11)(q21;q23) | MLL-AF4 | NO | Good |
|  | 10 | 34 | M | 90 | 46 XY | - | NO | Good |
|  | 11 | 18 | M | 90 | 46 XY | - | NO | Very good |

**Table S3. *TP53* Primer sequences.** For each primer pair, the sequence (5’- 3’), the melting temperature (Tm), the length and the amplicon size are reported. Primers have been designed using Primer3 (v. 0.4.0) Software Tool.

| **Primer ID** | **Sequence 5’- 3’** | **Tm (C°)** | **Length** | **Amplicon size (bp)** |
| --- | --- | --- | --- | --- |
| p53 F1 | TGGATTGGCAGCCAGACT | 60.36 | 18 | 491 |
| p53 R1 | GGGGGTGTGGAATCAACC | 61.01 | 18 |  |
| p53 F2 | TCAACAAGATGTTTTGCCAACT | 59.50 | 22 | 482 |
| p53 R2 | GCGGAGATTCTCTTCCTCTGT | 59.97 | 21 |  |
| p53 F3 | GGTAATCTACTGGGACGGAACA | 60.24 | 22 | 498 |
| p53 R3 | CTATTGCAAGCAAGGGTTCAA | 60.25 | 21 |  |

**Table S4.** Antibody reactivity and sources as well as the antigen retrieval protocols, dilutions and revelation systems used in immunohistochemistry.

| **molecule** | **antibody type** | **clone** | **source** | **dilution** | **retrieval** | **revelation system** |
| --- | --- | --- | --- | --- | --- | --- |
| CHK1 | Rabbit polyclonal |  | NOVUS : NB100-91696 | 1:20 | PtLink, low pH 97°C x 20’ | K8012* |
| p-CHK1(Ser 345) | Rabbit polyclonal |  | NOVUS: NBPI-60799 | 1:20 | PtLink, low pH 97°C x 20’ | K8012* |
| CHK2 | Rabbit monoclonal | EPR4325 | EPITOMICS: 3428-1 | 1:400 | PtLink, high pH 92°C x 5’ | K5005^#^ |
| p-CHK2(Thr 68) | Rabbit polyclonal |  | CELL SIGNALING: 2661S | 1:200 | PtLink, high pH 92°C x 5’ | K5005^#^ |
| CDc25C | Rabbit monoclonal | E302 | EPITOMICS: 1302-1 | 1:9600 | PtLink, high pH 92°C x 5’ | K5005^#^ |
| p-CDc25C (Ser216) | Rabbit polyclonal |  | NOVUS: NB 100-92494 | 1:20 | PC+MW900W^§^ X 3’+TRIS EDTA | K8012* |
| p-H2AX(Ser 319) | Rabbit polyclonal |  | CELL SIGNALING: 2577S | 1:25 | PtLink, high pH 92°C x 5’ | K5005^#^ |

*K8012: Envision Flex, (Dako Cytomation, Denmark);  ^#^ K5005:Real Detection System Alkaline Phosphatase/RED rabbit/mouse (Dako Cytomation, Denmark); ^§^ PC+MW900W : microwaveable pressure cooker at 900W, timing 3 minutes when at pressure.
