# Supplementary material for: In vitro and in vivo single-agent efficacy of checkpoint kinase inhibition in acute lymphoblastic leukemia
Source: J Hematol Oncol. 2015 Nov 5;8:125. doi: 10.1186/s13045-015-0206-5 (PMC4635624; doi:10.1186/s13045-015-0206-5)
Supplement: Additional file 3: — Enrichment analysis report: enrichment by GO processes. (PDF 41 kb) [file 13045_2015_206_MOESM3_ESM.pdf]

Enrichment analysis report

Enrichment by GO Processes

#	Processes	Total	pValue	Min FDR	p-value	FDR	In Data	Genes from Active Data
1	cellular metabolic process	9418	9.28E-44	4.86E-40	9.28E-44	4.86E-40	617	FBXO45, APG3, DNA2, ZNF668, PACT, Forkhead box protein D4-like 2, GTF2IRD2B, G3P2, PHAP1 (pp32), RFC4, BTEB4, LOC440093, ZSCAN4, dUTPase (DUT), CTP synthase, POLE3-POLE4 complex, Twinfilin, Peroxiredoxin, Tubulin beta, CAPER, DPM2 (reg), MIWI, TRUB2, FIGNL1, FUBP1, Ku70/80, REV-ERBalpha, IRF7, FAF1, Dyskerin (NAP57), C9orf105, TK2, NOL8, TR-alpha, SC4MOL, hnRNP U, CREB1, PCNA, PDK1, JMJD2A, ZNF441, Probable cation-transporting ATPase 13A3, GSTM2, C/EBP, TOM40, MCM6, MGST3, CTE2, Histone H2AC, Ku80, Urocortin 2, SNRN25, Tip60, H protein N6-lipoyllysine, EIF1AX, GRINL1A, PERC, UQCR11, Sen34, PLK3 (CNK), C5orf41, Tubulin alpha-1B, MANEA, HMGI/Y, CAD, TER, FARSB, hnRNP H3, IKBZ, Cyclophilin H, GADD45 alpha, , Chk2, YB-1, PGHD, FAST kinase domain-containing protein 3, FBXO8, DDX54, MIF, PSMB3, MCM3, eIF2B3, EGR1, RNPS1, PUR2, ACTC, MRM1, PTOP, HAT1, ABCC4, RBM6, HSP90 alpha, PLA2G4C, INTS6, RAD18, ING4, SSRP1, G3BP1 (hdhVIII), PSMD1, CSNK1G1, SRP72, MRPL4, NDUFA2, Forkhead box protein D4-like 2, SLA/LP, JOSD3, Ku70, PSMD2, C/EBPgamma, HIST1H2BD, EGR2 (Krox20), Casein kinase I, c-Jun/c-Fos, ROCK, PP2A regulatory, NDUFB2, FASP1, ZNF425, CARM1, Importin (karyopherin)-beta, DDB1, ZNF561, MSK1/2 (RPS6KA5/4), Dynein, axonemal, heavy chains, GLUT1, EAF1, EWS, PRMT1, JunD/c-Fos, HIST1H2BG, Skp2/TrCP/FBXW, Histone H3.3, TFE3, HSP10 (mitochondrial), Serotonin receptor, EWS/WT1 fusion protein, C1orf124, PREI4, HIST2H2BC, MTMR4, XTP3TPA, EPC1, STK19, ChAF1 subunit B, ACACA, COX VIb-1, PAXIP1L, PTMA, TCP1-delta, UQCR10, hnRNP R, hnRNP L, NAP2, RNF12, HIST2H2BF, RFX3, PSMC6, 4E-BP2, XRCC2, SLC25A10, ZBTB10, ZNF491, UBC7, AP-1, HSST4, IBRDC3, Galpha(i)-specific amine GPCRs, AKD1, TLK2, SLBP, E2N(UBC13), Protein kinase G1, COQ4, KIAA0152, ACLY, PSMD14, PAF49, NF-kB2 (p100), B4GT2, TXNIP (VDUP1), WARS, KLF2, CDK5, APLF, GADD45 gamma, ATP1A1, TALDO, TARDBP (TDP43), Acsm5, C17orf79, APOA2, SPSY, COX Va, SLC19A1, CGI-51, eIF4A2, PSMB7, EWS/E1AF fusion protein, Cyclin L1, RPA1, SKP2, C10orf119, NDUFV2, LSM2, ENO1, Tubulin beta 1, C/EBP zeta, FBI-1 (Pokemon), WDR77, Galpha(q)-specific peptide GPCRs, STRA13, HDAC3, Forkhead box protein D4-like 2, Tubulin alpha, ARD1, Rad52, Cofilin, non-muscle, MCPIP, HIST1H2BL, NOR1, ALDOA, POLR3H, HIST1H2BJ, MVD, Forkhead box protein D4-like 6, TUFM, PIG8, PGAM1, GCTG, ENO, NUAK1, PP2C, RNF34, PIGW, FDPS, MOGAT2, Forkhead box protein D4-like 6, NF-kB, HIST1H2AK, TADA1L, BTG2, SETDB1, IGF2BP3, CLK1, F264, CGAT2, CHKB, GKP3, ZNF431, HIST2H2BE, RelB (NF-kB subunit), FosB/JunB, PCYOX1, RAD23B, TAFs, PCPH, Calmodulin, ZNF479, DIRAS2, PDHA (testis), CaMK II gamma, ZNF708, MSK2, SPEE, MCM2, ATP5G1, Histone H3.1, GMPS, HSC70, LRGUK, POLD reg (p50), ZNF791, FEN1, ZNF226, H protein N6-dihydrolipoyllysine, IRE1, ERO1, ALY, PPIAL4, CG beta, Cyclin T2, Ataxin-7, ZNF678, C14orf126, RHO6, Thyroid hormone receptor, PMP34, VARSL, Histone H3, Aggrecanase-1, WAC, POLD cat (p125), HSP60, SOD1, GLUT4, mTERFD2, TCP1-zeta-1, GINS2, PUS7, PDH alpha, MRPL12, MED29, c-Fos, ZNF213, MOGAT3, ASXL3, MCM5, MAP1LC3B, GLT25D1, PLC-beta, JunD, DPH2, UBE2G1, Ephrin-A, HSP90, DPY30, Gemin5, RPP40, Calpain 1(mu), Zinc finger protein 394, EWS/FLI1 fusion protein, RBM14, PRDX4, Histone H2BO, SMVT, ZNF674, NOLA2 (NHP2), ATP5C, FUMH, JunB, TFIID 31 kDa subunit, TRIP13, C16orf42, HIST2H2AC, CHIP, Cullin 2, PLA2, CDK4, LPIN3, LDHA, UBE2S, UQCRQPC, GCSH, TFIIF, alpha subunit, HIST2H2AB, FOXD4L4, UQCRH, ERO1Lalpha, DNA polymerase nu, HIST1H2AH, NDPK A, FASTKD3, FUS/DDIT3 fusion protein, NDUFAB1, HAND2, DUSP8, Puromycin-sensitive aminopeptidase, NDUFA1, DNAH1, IPPK, SFRS1 (SF2), CERCAM, NeuN, NCOA3 (pCIP/SRC3), MafF, NPEPPS, FANCD2, HSP70, PUR9, EAR2, WIP1, RUVBL2, JMJD3, POLE3 (YBL1), NUP98/HHEX fusion protein, Histone H2A.o, Rod1, hnRNP C, DHFRL1, Ndg2, Utp15, MRPL19, HXK2, ATP5G, NDPK complex, RalA, FASN, Tyk2, SNRPD1 (SMD1), Forkhead box protein D4-like 6, SLC25A39, FOXD4, USP52, PAFAH alpha (LIS1), ING3, CISY, PP1-cat gamma, EWS/ER81 fusion protein, ATF-3, MUS81, HEXIM1, OLAH, RAD1, Rad51, WDR5, C1TC, TTLL12, CGB, NS1-BP, TFIIE, beta subunit, COX17, GTF2IRD2, SNRP116, PDSS1, ZNF813, 4EHP, Rab-3, SUV420H1, TRAP-1, RBBP7 (RbAp46), TRAP95, C/EBPbeta, RAI, GTPBP3, MRPL18, IARS2, SUPT16H, ALDOC, PLC-beta3, HA95, IL-8, DZIP3, Leptin, ACAT2, WDR61, Probable D-tyrosyl-tRNA(T, Claspin, HIST1H2BN, SAKS1, p66alpha, S-acyl fatty acid synthase thioesterase, medium chain, cPLA2, LEO1, HIST1H2AE, nNOS, HPRT, ERAP140, HMGCL, CNOT3, COX VIa, Actin, p19, NUDT5, TCP1-zeta-1/2, ZNF454, PP1-cat, VKORC1L1, Rac3, Forkhead box protein D4-like 6, eIF1A, SERB, Histone H2, HP1 gamma, JAK3, PPIL3, URI, Histone H2A, WRN, DHFR, ANAPC5, Elk-1, B3gnt8, ZFP36(Tristetraprolin), c-Jun/c-Jun, PAI1, Splicing factor 4, PHF13, NDUFS6, Histone H1.2, snRNP-F, CHCHD4, FTSJ1, MAZ, Foxd4L1, ADSL, COX VIb, RPP30, NDPK B, MDH1, NOM1, SIN1, PSMA2, NIPK, HP1, UBL4A, FBXO10, PUR6, RBM5, Forkhead box protein D4-like 2, SBNO2, JPO1, Galpha(s)-specific glucagon GPCRs, PLCE, FAST kinase domain-containing protein 3, RRM1, F-box only protein 10, SAP25, Dcp1b, Nucleolar protein 8, DHX15, SET, Histone H2B, FAM20B, EWS/ERG fusion protein, Trp53inp2, DDX46, CNT2, KHK, Rab-3A, FOXD4L5, DNAJB2, FLAD1, SerRS, ZNF721, PYCS, PUR1, Tubulin alpha 3, SNRPA, PAK2, TBR1, TCOF1, NF-kB p52/RelB, Acyl-CoA synthetase, FLJ10292, MRPL17, ZNF394, MUSK, HIST1H3D, GSPT1, Forkhead box protein D4-like 6, eIF4A, Histone H4, CD147, PP2C gamma, COX Vb, MPP alpha, RCOR3, LCMR1, COX VIa-1, TTBK2, CPT-1B, KAD6, DHODH, NUP98, ATP13A3, BTG1, SF4, SERTAD1, ROCK1, ACSL3, NIP3, TBDN100, CYHR1, KLF6, CENPP, TBX20, MAT2A, Betacellulin, NUP98/HOXA9 fusion protein, Cofilin, c-Jun, Neuroglycan C, HIST1H2AD, C1orf69, AARS, Actin muscle, SOX30, Frataxin, Phosphatase regulator (inhibitor), Octanoyl-holo-acp, p23 co-chaperone, BCCIP, HIST1H2AJ, CENP-H, HERP, Protein kinase G, Pc2, Ran, Tubulin alpha 3E, Glycosyltransferase 25 family member 3 precursor, RLI, Ebp1, POLN, PGAM4, Histone H1, SP3, Ca-ATPase2, TREX2, NEUR1, Aciculin, USP5, Histone H1.3, PUR4, Cyclophilin A, ASGR2, CDK2, 3'HEXO, KLHDC3, SOCS1, SET/NUP214 fusion protein, Tubulin (in microtubules), TCP1-epsilon, CDC45L, SLC25A1, RPIA, 14-3-3, VEGF-B
2	nitrogen compound metabolic process	5560	5.23E-43	1.3E-39	5.23E-43	1.3E-39	432	DNA2, ZNF668, PACT, Forkhead box protein D4-like 2, GTF2IRD2B, PHAP1 (pp32), RFC4, BTEB4, LOC440093, ZSCAN4, dUTPase (DUT), CTP synthase, POLE3-POLE4 complex, Peroxiredoxin, Tubulin beta, CAPER, MIWI, TRUB2, FIGNL1, FUBP1, Ku70/80, REV-ERBalpha, IRF7, Dyskerin (NAP57), TK2, NOL8, TR-alpha, hnRNP U, CREB1, PCNA, JMJD2A, ZNF441, Probable cation-transporting ATPase 13A3, C/EBP, MCM6, Histone H2AC, Ku80, Urocortin 2, SNRN25, Tip60, GRINL1A, PERC, Sen34, C5orf41, Tubulin alpha-1B, HMGI/Y, CAD, FARSB, hnRNP H3, IKBZ, Cyclophilin H, GADD45 alpha, Chk2, YB-1, DDX54, PSMB3, MCM3, EGR1, RNPS1, PUR2, ACTC, MRM1, PTOP, HAT1, ABCC4, RBM6, HSP90 alpha, PLA2G4C, INTS6, RAD18, ING4, SSRP1, G3BP1 (hdhVIII), PSMD1, Forkhead box protein D4-like 2, SLA/LP, JOSD3, Ku70, PSMD2, C/EBPgamma, HIST1H2BD, EGR2 (Krox20), Casein kinase I, c-Jun/c-Fos, ROCK, PP2A regulatory, FASP1, ZNF425, CARM1, Importin (karyopherin)-beta, DDB1, ZNF561, Dynein, axonemal, heavy chains, EAF1, EWS, JunD/c-Fos, HIST1H2BG, Histone H3.3, TFE3, Serotonin receptor, EWS/WT1 fusion protein, C1orf124, HIST2H2BC, XTP3TPA, EPC1, ChAF1 subunit B, ACACA, PAXIP1L, PTMA, hnRNP R, hnRNP L, NAP2, RNF12, HIST2H2BF, RFX3, PSMC6, XRCC2, SLC25A10, ZBTB10, ZNF491, AP-1, Galpha(i)-specific amine GPCRs, AKD1, SLBP, E2N(UBC13), ACLY, PSMD14, PAF49, NF-kB2 (p100), TXNIP (VDUP1), WARS, KLF2, APLF, GADD45 gamma, ATP1A1, TALDO, TARDBP (TDP43), C17orf79, APOA2, SPSY, SLC19A1, eIF4A2, PSMB7, EWS/E1AF fusion protein, Cyclin L1, RPA1, C10orf119, LSM2, ENO1, Tubulin beta 1, C/EBP zeta, FBI-1 (Pokemon), WDR77, STRA13, HDAC3, Forkhead box protein D4-like 2, Tubulin alpha, ARD1, Rad52, MCPIP, HIST1H2BL, NOR1, ALDOA, POLR3H, HIST1H2BJ, Forkhead box protein D4-like 6, TUFM, ENO, PP2C, Forkhead box protein D4-like 6, NF-kB, HIST1H2AK, TADA1L, BTG2, SETDB1, CHKB, ZNF431, HIST2H2BE, RelB (NF-kB subunit), FosB/JunB, RAD23B, TAFs, PCPH, Calmodulin, ZNF479, DIRAS2, ZNF708, SPEE, MCM2, ATP5G1, Histone H3.1, GMPS, HSC70, POLD reg (p50), ZNF791, FEN1, ZNF226, IRE1, ALY, CG beta, Cyclin T2, Ataxin-7, ZNF678, RHO6, Thyroid hormone receptor, VARSL, Histone H3, WAC, POLD cat (p125), HSP60, SOD1, mTERFD2, GINS2, PUS7, MRPL12, MED29, c-Fos, ZNF213, ASXL3, MCM5, PLC-beta, JunD, HSP90, DPY30, Gemin5, RPP40, Zinc finger protein 394, EWS/FLI1 fusion protein, RBM14, Histone H2BO, SMVT, ZNF674, NOLA2 (NHP2), ATP5C, JunB, TFIID 31 kDa subunit, TRIP13, C16orf42, HIST2H2AC, CHIP, PLA2, LPIN3, LDHA, TFIIF, alpha subunit, HIST2H2AB, FOXD4L4, DNA polymerase nu, HIST1H2AH, NDPK A, FUS/DDIT3 fusion protein, HAND2, Myosin I, DNAH1, SFRS1 (SF2), NeuN, NCOA3 (pCIP/SRC3), MafF, FANCD2, HSP70, PUR9, EAR2, RUVBL2, POLE3 (YBL1), NUP98/HHEX fusion protein, Histone H2A.o, Rod1, hnRNP C, DHFRL1, Ndg2, Utp15, ATP5G, NDPK complex, RalA, SNRPD1 (SMD1), Forkhead box protein D4-like 6, SLC25A39, FOXD4, USP52, PAFAH alpha (LIS1), ING3, EWS/ER81 fusion protein, ATF-3, MUS81, HEXIM1, RAD1, Rad51, WDR5, C1TC, CGB, NS1-BP, TFIIE, beta subunit, GTF2IRD2, SNRP116, ZNF813, Rab-3, SUV420H1, RBBP7 (RbAp46), TRAP95, C/EBPbeta, RAI, GTPBP3, IARS2, SUPT16H, WDR61, Claspin, HIST1H2BN, p66alpha, cPLA2, LEO1, HIST1H2AE, nNOS, HPRT, ERAP140, CNOT3, Actin, p19, NUDT5, ZNF454, Rac3, Forkhead box protein D4-like 6, SERB, Histone H2, HP1 gamma, PPIL3, URI, Histone H2A, WRN, DHFR, Elk-1, ZFP36(Tristetraprolin), c-Jun/c-Jun, PAI1, Splicing factor 4, PHF13, Histone H1.2, snRNP-F, FTSJ1, MAZ, Foxd4L1, ADSL, RPP30, NDPK B, MDH1, NOM1, PSMA2, NIPK, HP1, PUR6, RBM5, Forkhead box protein D4-like 2, SBNO2, JPO1, RRM1, SAP25, Dcp1b, Nucleolar protein 8, DHX15, SET, Histone H2B, EWS/ERG fusion protein, Trp53inp2, DDX46, CNT2, Rab-3A, FOXD4L5, FLAD1, SerRS, ZNF721, PYCS, PUR1, Tubulin alpha 3, SNRPA, TBR1, TCOF1, NF-kB p52/RelB, FLJ10292, ZNF394, HIST1H3D, GSPT1, Forkhead box protein D4-like 6, eIF4A, Histone H4, RCOR3, LCMR1, KAD6, DHODH, NUP98, ATP13A3, SF4, SERTAD1, NIP3, TBDN100, KLF6, CENPP, TBX20, MAT2A, Betacellulin, NUP98/HOXA9 fusion protein, c-Jun, HIST1H2AD, C1orf69, AARS, Actin muscle, SOX30, Frataxin, Phosphatase regulator (inhibitor), p23 co-chaperone, BCCIP, HIST1H2AJ, CENP-H, Protein kinase G, Pc2, Ran, Tubulin alpha 3E, RLI, Ebp1, POLN, Histone H1, SP3, Ca-ATPase2, TREX2, Histone H1.3, PUR4, Cyclophilin A, CDK2, 3'HEXO, KLHDC3, SET/NUP214 fusion protein, Tubulin (in microtubules), CDC45L, RPIA, 14-3-3
3	cellular nitrogen compound metabolic process	5490	7.46E-43	1.3E-39	7.46E-43	1.3E-39	428	DNA2, ZNF668, PACT, Forkhead box protein D4-like 2, GTF2IRD2B, PHAP1 (pp32), RFC4, BTEB4, LOC440093, ZSCAN4, dUTPase (DUT), CTP synthase, POLE3-POLE4 complex, Peroxiredoxin, Tubulin beta, CAPER, MIWI, TRUB2, FIGNL1, FUBP1, Ku70/80, REV-ERBalpha, IRF7, Dyskerin (NAP57), TK2, NOL8, TR-alpha, hnRNP U, CREB1, PCNA, JMJD2A, ZNF441, Probable cation-transporting ATPase 13A3, C/EBP, MCM6, Histone H2AC, Ku80, Urocortin 2, SNRN25, Tip60, GRINL1A, PERC, Sen34, C5orf41, Tubulin alpha-1B, HMGI/Y, CAD, FARSB, hnRNP H3, IKBZ, Cyclophilin H, GADD45 alpha, Chk2, YB-1, DDX54, PSMB3, MCM3, EGR1, RNPS1, PUR2, ACTC, MRM1, PTOP, HAT1, ABCC4, RBM6, HSP90 alpha, PLA2G4C, INTS6, RAD18, ING4, SSRP1, G3BP1 (hdhVIII), PSMD1, Forkhead box protein D4-like 2, SLA/LP, JOSD3, Ku70, PSMD2, C/EBPgamma, HIST1H2BD, EGR2 (Krox20), Casein kinase I, c-Jun/c-Fos, ROCK, PP2A regulatory, FASP1, ZNF425, CARM1, Importin (karyopherin)-beta, DDB1, ZNF561, Dynein, axonemal, heavy chains, EAF1, EWS, JunD/c-Fos, HIST1H2BG, Histone H3.3, TFE3, Serotonin receptor, EWS/WT1 fusion protein, C1orf124, HIST2H2BC, XTP3TPA, EPC1, ChAF1 subunit B, ACACA, PAXIP1L, PTMA, hnRNP R, hnRNP L, NAP2, RNF12, HIST2H2BF, RFX3, PSMC6, XRCC2, SLC25A10, ZBTB10, ZNF491, AP-1, Galpha(i)-specific amine GPCRs, AKD1, SLBP, E2N(UBC13), ACLY, PSMD14, PAF49, NF-kB2 (p100), TXNIP (VDUP1), WARS, KLF2, APLF, GADD45 gamma, ATP1A1, TALDO, TARDBP (TDP43), C17orf79, APOA2, SPSY, eIF4A2, PSMB7, EWS/E1AF fusion protein, Cyclin L1, RPA1, C10orf119, LSM2, ENO1, Tubulin beta 1, C/EBP zeta, FBI-1 (Pokemon), WDR77, STRA13, HDAC3, Forkhead box protein D4-like 2, Tubulin alpha, ARD1, Rad52, MCPIP, HIST1H2BL, NOR1, ALDOA, POLR3H, HIST1H2BJ, Forkhead box protein D4-like 6, TUFM, ENO, PP2C, Forkhead box protein D4-like 6, NF-kB, HIST1H2AK, TADA1L, BTG2, SETDB1, CHKB, ZNF431, HIST2H2BE, RelB (NF-kB subunit), FosB/JunB, RAD23B, TAFs, PCPH, ZNF479, DIRAS2, ZNF708, SPEE, MCM2, ATP5G1, Histone H3.1, GMPS, HSC70, POLD reg (p50), ZNF791, FEN1, ZNF226, IRE1, ALY, CG beta, Cyclin T2, Ataxin-7, ZNF678, RHO6, Thyroid hormone receptor, VARSL, Histone H3, WAC, POLD cat (p125), HSP60, SOD1, mTERFD2, GINS2, PUS7, MRPL12, MED29, c-Fos, ZNF213, ASXL3, MCM5, PLC-beta, JunD, HSP90, DPY30, Gemin5, RPP40, Zinc finger protein 394, EWS/FLI1 fusion protein, RBM14, Histone H2BO, SMVT, ZNF674, NOLA2 (NHP2), ATP5C, JunB, TFIID 31 kDa subunit, TRIP13, C16orf42, HIST2H2AC, CHIP, PLA2, LPIN3, LDHA, TFIIF, alpha subunit, HIST2H2AB, FOXD4L4, DNA polymerase nu, HIST1H2AH, NDPK A, FUS/DDIT3 fusion protein, HAND2, DNAH1, SFRS1 (SF2), NeuN, NCOA3 (pCIP/SRC3), MafF, FANCD2, HSP70, PUR9, EAR2, RUVBL2, POLE3 (YBL1), NUP98/HHEX fusion protein, Histone H2A.o, Rod1, hnRNP C, DHFRL1, Ndg2, Utp15, ATP5G, NDPK complex, RalA, SNRPD1 (SMD1), Forkhead box protein D4-like 6, SLC25A39, FOXD4, USP52, PAFAH alpha (LIS1), ING3, EWS/ER81 fusion protein, ATF-3, MUS81, HEXIM1, RAD1, Rad51, WDR5, C1TC, CGB, NS1-BP, TFIIE, beta subunit, GTF2IRD2, SNRP116, ZNF813, Rab-3, SUV420H1, RBBP7 (RbAp46), TRAP95, C/EBPbeta, RAI, GTPBP3, IARS2, SUPT16H, WDR61, Claspin, HIST1H2BN, p66alpha, cPLA2, LEO1, HIST1H2AE, nNOS, HPRT, ERAP140, CNOT3, Actin, p19, NUDT5, ZNF454, Rac3, Forkhead box protein D4-like 6, SERB, Histone H2, HP1 gamma, PPIL3, URI, Histone H2A, WRN, DHFR, Elk-1, ZFP36(Tristetraprolin), c-Jun/c-Jun, PAI1, Splicing factor 4, PHF13, Histone H1.2, snRNP-F, FTSJ1, MAZ, Foxd4L1, ADSL, RPP30, NDPK B, MDH1, NOM1, PSMA2, NIPK, HP1, PUR6, RBM5, Forkhead box protein D4-like 2, SBNO2, JPO1, RRM1, SAP25, Dcp1b, Nucleolar protein 8, DHX15, SET, Histone H2B, EWS/ERG fusion protein, Trp53inp2, DDX46, CNT2, Rab-3A, FOXD4L5, FLAD1, SerRS, ZNF721, PYCS, PUR1, Tubulin alpha 3, SNRPA, TBR1, TCOF1, NF-kB p52/RelB, FLJ10292, ZNF394, HIST1H3D, GSPT1, Forkhead box protein D4-like 6, eIF4A, Histone H4, RCOR3, LCMR1, KAD6, DHODH, NUP98, ATP13A3, SF4, SERTAD1, NIP3, TBDN100, KLF6, CENPP, TBX20, MAT2A, Betacellulin, NUP98/HOXA9 fusion protein, c-Jun, HIST1H2AD, C1orf69, AARS, Actin muscle, SOX30, Frataxin, Phosphatase regulator (inhibitor), p23 co-chaperone, BCCIP, HIST1H2AJ, CENP-H, Pc2, Ran, Tubulin alpha 3E, RLI, Ebp1, POLN, Histone H1, SP3, Ca-ATPase2, TREX2, Histone H1.3, PUR4, Cyclophilin A, CDK2, 3'HEXO, KLHDC3, SET/NUP214 fusion protein, Tubulin (in microtubules), CDC45L, RPIA, 14-3-3
4	nucleobase-containing compound metabolic process	5145	1.86E-42	2.44E-39	1.86E-42	2.44E-39	409	DNA2, ZNF668, PACT, Forkhead box protein D4-like 2, GTF2IRD2B, PHAP1 (pp32), RFC4, BTEB4, LOC440093, ZSCAN4, dUTPase (DUT), CTP synthase, POLE3-POLE4 complex, Peroxiredoxin, Tubulin beta, CAPER, MIWI, TRUB2, FIGNL1, FUBP1, Ku70/80, REV-ERBalpha, IRF7, Dyskerin (NAP57), TK2, NOL8, TR-alpha, hnRNP U, CREB1, PCNA, JMJD2A, ZNF441, Probable cation-transporting ATPase 13A3, C/EBP, MCM6, Histone H2AC, Ku80, Urocortin 2, SNRN25, Tip60, GRINL1A, PERC, Sen34, C5orf41, Tubulin alpha-1B, HMGI/Y, CAD, FARSB, hnRNP H3, IKBZ, Cyclophilin H, GADD45 alpha, Chk2, YB-1, DDX54, PSMB3, MCM3, EGR1, RNPS1, PUR2, ACTC, MRM1, PTOP, HAT1, ABCC4, RBM6, HSP90 alpha, INTS6, RAD18, ING4, SSRP1, G3BP1 (hdhVIII), PSMD1, Forkhead box protein D4-like 2, SLA/LP, JOSD3, Ku70, PSMD2, C/EBPgamma, HIST1H2BD, EGR2 (Krox20), Casein kinase I, c-Jun/c-Fos, ROCK, PP2A regulatory, FASP1, ZNF425, CARM1, Importin (karyopherin)-beta, DDB1, ZNF561, Dynein, axonemal, heavy chains, EAF1, EWS, JunD/c-Fos, HIST1H2BG, Histone H3.3, TFE3, Serotonin receptor, EWS/WT1 fusion protein, C1orf124, HIST2H2BC, XTP3TPA, EPC1, ChAF1 subunit B, ACACA, PAXIP1L, PTMA, hnRNP R, hnRNP L, NAP2, RNF12, HIST2H2BF, RFX3, PSMC6, XRCC2, ZBTB10, ZNF491, AP-1, Galpha(i)-specific amine GPCRs, AKD1, SLBP, E2N(UBC13), ACLY, PSMD14, PAF49, NF-kB2 (p100), TXNIP (VDUP1), WARS, KLF2, APLF, GADD45 gamma, ATP1A1, TALDO, TARDBP (TDP43), C17orf79, eIF4A2, PSMB7, EWS/E1AF fusion protein, Cyclin L1, RPA1, C10orf119, LSM2, ENO1, Tubulin beta 1, C/EBP zeta, FBI-1 (Pokemon), WDR77, STRA13, HDAC3, Forkhead box protein D4-like 2, Tubulin alpha, ARD1, Rad52, MCPIP, HIST1H2BL, NOR1, ALDOA, POLR3H, HIST1H2BJ, Forkhead box protein D4-like 6, TUFM, ENO, PP2C, Forkhead box protein D4-like 6, NF-kB, HIST1H2AK, TADA1L, BTG2, SETDB1, ZNF431, HIST2H2BE, RelB (NF-kB subunit), FosB/JunB, RAD23B, TAFs, PCPH, ZNF479, DIRAS2, ZNF708, MCM2, ATP5G1, Histone H3.1, GMPS, HSC70, POLD reg (p50), ZNF791, FEN1, ZNF226, IRE1, ALY, Cyclin T2, Ataxin-7, ZNF678, RHO6, Thyroid hormone receptor, VARSL, Histone H3, WAC, POLD cat (p125), HSP60, SOD1, mTERFD2, GINS2, PUS7, MRPL12, MED29, c-Fos, ZNF213, ASXL3, MCM5, PLC-beta, JunD, HSP90, DPY30, Gemin5, RPP40, Zinc finger protein 394, EWS/FLI1 fusion protein, RBM14, Histone H2BO, ZNF674, NOLA2 (NHP2), ATP5C, JunB, TFIID 31 kDa subunit, TRIP13, C16orf42, HIST2H2AC, CHIP, LDHA, TFIIF, alpha subunit, HIST2H2AB, FOXD4L4, DNA polymerase nu, HIST1H2AH, NDPK A, FUS/DDIT3 fusion protein, HAND2, DNAH1, SFRS1 (SF2), NeuN, NCOA3 (pCIP/SRC3), MafF, FANCD2, HSP70, PUR9, EAR2, RUVBL2, POLE3 (YBL1), NUP98/HHEX fusion protein, Histone H2A.o, Rod1, hnRNP C, DHFRL1, Ndg2, Utp15, ATP5G, NDPK complex, RalA, SNRPD1 (SMD1), Forkhead box protein D4-like 6, FOXD4, USP52, ING3, EWS/ER81 fusion protein, ATF-3, MUS81, HEXIM1, RAD1, Rad51, WDR5, C1TC, NS1-BP, TFIIE, beta subunit, GTF2IRD2, SNRP116, ZNF813, Rab-3, SUV420H1, RBBP7 (RbAp46), TRAP95, C/EBPbeta, RAI, GTPBP3, IARS2, SUPT16H, WDR61, Claspin, HIST1H2BN, p66alpha, LEO1, HIST1H2AE, HPRT, ERAP140, CNOT3, Actin, p19, NUDT5, ZNF454, Rac3, Forkhead box protein D4-like 6, Histone H2, HP1 gamma, PPIL3, URI, Histone H2A, WRN, DHFR, Elk-1, ZFP36(Tristetraprolin), c-Jun/c-Jun, PAI1, Splicing factor 4, PHF13, Histone H1.2, snRNP-F, FTSJ1, MAZ, Foxd4L1, ADSL, RPP30, NDPK B, MDH1, NOM1, PSMA2, NIPK, HP1, PUR6, RBM5, Forkhead box protein D4-like 2, SBNO2, JPO1, RRM1, SAP25, Dcp1b, Nucleolar protein 8, DHX15, SET, Histone H2B, EWS/ERG fusion protein, Trp53inp2, DDX46, CNT2, Rab-3A, FOXD4L5, FLAD1, SerRS, ZNF721, PUR1, Tubulin alpha 3, SNRPA, TBR1, TCOF1, NF-kB p52/RelB, FLJ10292, ZNF394, HIST1H3D, GSPT1, Forkhead box protein D4-like 6, eIF4A, Histone H4, RCOR3, LCMR1, KAD6, DHODH, NUP98, ATP13A3, SF4, SERTAD1, NIP3, TBDN100, KLF6, CENPP, TBX20, MAT2A, Betacellulin, NUP98/HOXA9 fusion protein, c-Jun, HIST1H2AD, AARS, Actin muscle, SOX30, Phosphatase regulator (inhibitor), p23 co-chaperone, BCCIP, HIST1H2AJ, CENP-H, Pc2, Ran, Tubulin alpha 3E, RLI, Ebp1, POLN, Histone H1, SP3, Ca-ATPase2, TREX2, Histone H1.3, PUR4, Cyclophilin A, CDK2, 3'HEXO, KLHDC3, SET/NUP214 fusion protein, Tubulin (in microtubules), CDC45L, RPIA, 14-3-3
5	heterocycle metabolic process	5330	5.92E-41	6.21E-38	5.92E-41	6.21E-38	415	DNA2, ZNF668, PACT, Forkhead box protein D4-like 2, GTF2IRD2B, PHAP1 (pp32), RFC4, BTEB4, LOC440093, ZSCAN4, dUTPase (DUT), CTP synthase, POLE3-POLE4 complex, Peroxiredoxin, Tubulin beta, CAPER, MIWI, TRUB2, FIGNL1, FUBP1, Ku70/80, REV-ERBalpha, IRF7, Dyskerin (NAP57), TK2, NOL8, TR-alpha, hnRNP U, CREB1, PCNA, JMJD2A, ZNF441, Probable cation-transporting ATPase 13A3, C/EBP, MCM6, Histone H2AC, Ku80, Urocortin 2, SNRN25, Tip60, GRINL1A, PERC, Sen34, C5orf41, Tubulin alpha-1B, HMGI/Y, CAD, FARSB, hnRNP H3, IKBZ, Cyclophilin H, GADD45 alpha, Chk2, YB-1, DDX54, PSMB3, MCM3, EGR1, RNPS1, PUR2, ACTC, MRM1, PTOP, HAT1, ABCC4, RBM6, HSP90 alpha, INTS6, RAD18, ING4, SSRP1, G3BP1 (hdhVIII), PSMD1, Forkhead box protein D4-like 2, SLA/LP, JOSD3, Ku70, PSMD2, C/EBPgamma, HIST1H2BD, EGR2 (Krox20), Casein kinase I, c-Jun/c-Fos, ROCK, PP2A regulatory, FASP1, ZNF425, CARM1, Importin (karyopherin)-beta, DDB1, ZNF561, Dynein, axonemal, heavy chains, EAF1, EWS, JunD/c-Fos, HIST1H2BG, Histone H3.3, TFE3, Serotonin receptor, EWS/WT1 fusion protein, C1orf124, HIST2H2BC, XTP3TPA, EPC1, ChAF1 subunit B, ACACA, PAXIP1L, PTMA, hnRNP R, hnRNP L, NAP2, RNF12, HIST2H2BF, RFX3, PSMC6, XRCC2, ZBTB10, ZNF491, AP-1, Galpha(i)-specific amine GPCRs, AKD1, SLBP, E2N(UBC13), ACLY, PSMD14, PAF49, NF-kB2 (p100), TXNIP (VDUP1), WARS, KLF2, APLF, GADD45 gamma, ATP1A1, TALDO, TARDBP (TDP43), C17orf79, SLC19A1, eIF4A2, PSMB7, EWS/E1AF fusion protein, Cyclin L1, RPA1, C10orf119, LSM2, ENO1, Tubulin beta 1, C/EBP zeta, FBI-1 (Pokemon), WDR77, STRA13, HDAC3, Forkhead box protein D4-like 2, Tubulin alpha, ARD1, Rad52, MCPIP, HIST1H2BL, NOR1, ALDOA, POLR3H, HIST1H2BJ, Forkhead box protein D4-like 6, TUFM, ENO, PP2C, Forkhead box protein D4-like 6, NF-kB, HIST1H2AK, TADA1L, BTG2, SETDB1, ZNF431, HIST2H2BE, RelB (NF-kB subunit), FosB/JunB, RAD23B, TAFs, PCPH, ZNF479, DIRAS2, ZNF708, MCM2, ATP5G1, Histone H3.1, GMPS, HSC70, POLD reg (p50), ZNF791, FEN1, ZNF226, IRE1, ALY, Cyclin T2, Ataxin-7, ZNF678, RHO6, Thyroid hormone receptor, VARSL, Histone H3, WAC, POLD cat (p125), HSP60, SOD1, mTERFD2, GINS2, PUS7, MRPL12, MED29, c-Fos, ZNF213, ASXL3, MCM5, PLC-beta, JunD, HSP90, DPY30, Gemin5, RPP40, Zinc finger protein 394, EWS/FLI1 fusion protein, RBM14, Histone H2BO, SMVT, ZNF674, NOLA2 (NHP2), ATP5C, JunB, TFIID 31 kDa subunit, TRIP13, C16orf42, HIST2H2AC, CHIP, LDHA, TFIIF, alpha subunit, HIST2H2AB, FOXD4L4, DNA polymerase nu, HIST1H2AH, NDPK A, FUS/DDIT3 fusion protein, HAND2, DNAH1, SFRS1 (SF2), NeuN, NCOA3 (pCIP/SRC3), MafF, FANCD2, HSP70, PUR9, EAR2, RUVBL2, POLE3 (YBL1), NUP98/HHEX fusion protein, Histone H2A.o, Rod1, hnRNP C, DHFRL1, Ndg2, Utp15, ATP5G, NDPK complex, RalA, SNRPD1 (SMD1), Forkhead box protein D4-like 6, SLC25A39, FOXD4, USP52, ING3, EWS/ER81 fusion protein, ATF-3, MUS81, HEXIM1, RAD1, Rad51, WDR5, C1TC, NS1-BP, TFIIE, beta subunit, GTF2IRD2, SNRP116, ZNF813, Rab-3, SUV420H1, RBBP7 (RbAp46), TRAP95, C/EBPbeta, RAI, GTPBP3, IARS2, SUPT16H, WDR61, Claspin, HIST1H2BN, p66alpha, LEO1, HIST1H2AE, HPRT, ERAP140, CNOT3, Actin, p19, NUDT5, ZNF454, Rac3, Forkhead box protein D4-like 6, Histone H2, HP1 gamma, PPIL3, URI, Histone H2A, WRN, DHFR, Elk-1, ZFP36(Tristetraprolin), c-Jun/c-Jun, PAI1, Splicing factor 4, PHF13, Histone H1.2, snRNP-F, FTSJ1, MAZ, Foxd4L1, ADSL, RPP30, NDPK B, MDH1, NOM1, PSMA2, NIPK, HP1, PUR6, RBM5, Forkhead box protein D4-like 2, SBNO2, JPO1, RRM1, SAP25, Dcp1b, Nucleolar protein 8, DHX15, SET, Histone H2B, EWS/ERG fusion protein, Trp53inp2, DDX46, CNT2, Rab-3A, FOXD4L5, FLAD1, SerRS, ZNF721, PYCS, PUR1, Tubulin alpha 3, SNRPA, TBR1, TCOF1, NF-kB p52/RelB, FLJ10292, ZNF394, HIST1H3D, GSPT1, Forkhead box protein D4-like 6, eIF4A, Histone H4, RCOR3, LCMR1, KAD6, DHODH, NUP98, ATP13A3, SF4, SERTAD1, NIP3, TBDN100, KLF6, CENPP, TBX20, MAT2A, Betacellulin, NUP98/HOXA9 fusion protein, c-Jun, HIST1H2AD, C1orf69, AARS, Actin muscle, SOX30, Frataxin, Phosphatase regulator (inhibitor), p23 co-chaperone, BCCIP, HIST1H2AJ, CENP-H, Pc2, Ran, Tubulin alpha 3E, RLI, Ebp1, POLN, Histone H1, SP3, Ca-ATPase2, TREX2, Histone H1.3, PUR4, Cyclophilin A, CDK2, 3'HEXO, KLHDC3, SET/NUP214 fusion protein, Tubulin (in microtubules), CDC45L, RPIA, 14-3-3
6	primary metabolic process	9619	5.97E-40	5.21E-37	5.97E-40	5.21E-37	616	FBXO45, APG3, DNA2, ZNF668, PACT, Forkhead box protein D4-like 2, GTF2IRD2B, CD69, G3P2, PHAP1 (pp32), RFC4, TfR1, BTEB4, LOC440093, ZSCAN4, dUTPase (DUT), CTP synthase, POLE3-POLE4 complex, Twinfilin, Peroxiredoxin, Tubulin beta, CAPER, DPM2 (reg), MIWI, TRUB2, FIGNL1, FUBP1, Ku70/80, REV-ERBalpha, IRF7, FAF1, Dyskerin (NAP57), C9orf105, TK2, NOL8, TR-alpha, SC4MOL, hnRNP U, CREB1, PCNA, PDK1, JMJD2A, ZNF441, Probable cation-transporting ATPase 13A3, GSTM2, C/EBP, TOM40, MCM6, IGKC, MGST3, CTE2, Histone H2AC, Ku80, Urocortin 2, SNRN25, Tip60, H protein N6-lipoyllysine, EIF1AX, GRINL1A, PERC, Sen34, PLK3 (CNK), C5orf41, Tubulin alpha-1B, MANEA, HMGI/Y, CAD, TER, FARSB, hnRNP H3, IKBZ, Cyclophilin H, GADD45 alpha, , Chk2, YB-1, PGHD, FAST kinase domain-containing protein 3, FBXO8, DDX54, MIF, PSMB3, MCM3, eIF2B3, EGR1, RNPS1, PUR2, ACTC, MRM1, PTOP, HAT1, ABCC4, RBM6, HSP90 alpha, PLA2G4C, INTS6, RAD18, ING4, SSRP1, G3BP1 (hdhVIII), PSMD1, CSNK1G1, SRP72, MRPL4, Forkhead box protein D4-like 2, SLA/LP, JOSD3, Ku70, PSMD2, C/EBPgamma, HIST1H2BD, EGR2 (Krox20), Casein kinase I, c-Jun/c-Fos, ROCK, PP2A regulatory, FASP1, ZNF425, CARM1, Importin (karyopherin)-beta, DDB1, ZNF561, MSK1/2 (RPS6KA5/4), Dynein, axonemal, heavy chains, GLUT1, EAF1, EWS, PRMT1, JunD/c-Fos, HIST1H2BG, Skp2/TrCP/FBXW, Histone H3.3, TFE3, HSP10 (mitochondrial), Serotonin receptor, EWS/WT1 fusion protein, C1orf124, PREI4, HIST2H2BC, MTMR4, XTP3TPA, NUP43, EPC1, NUP205, STK19, ChAF1 subunit B, ACACA, PAXIP1L, MSP, PTMA, TCP1-delta, hnRNP R, hnRNP L, NAP2, RNF12, HIST2H2BF, RFX3, PSMC6, 4E-BP2, XRCC2, SLC25A10, Kappa chain (Ig light chain), ZBTB10, ZNF491, UBC7, AP-1, HSST4, IBRDC3, Galpha(i)-specific amine GPCRs, AKD1, TLK2, SLBP, E2N(UBC13), Protein kinase G1, KIAA0152, ACLY, PSMD14, PAF49, NF-kB2 (p100), B4GT2, TXNIP (VDUP1), WARS, KLF2, CDK5, APLF, GADD45 gamma, ATP1A1, TALDO, TARDBP (TDP43), CRIg, Acsm5, C17orf79, MMP-8, APOA2, SPSY, SLC19A1, CGI-51, eIF4A2, PSMB7, EWS/E1AF fusion protein, Cyclin L1, RPA1, SKP2, C10orf119, LSM2, ENO1, Tubulin beta 1, C/EBP zeta, FBI-1 (Pokemon), WDR77, Galpha(q)-specific peptide GPCRs, STRA13, HDAC3, Forkhead box protein D4-like 2, Tubulin alpha, ARD1, Rad52, Cofilin, non-muscle, MCPIP, HIST1H2BL, NOR1, ALDOA, POLR3H, HIST1H2BJ, MVD, Forkhead box protein D4-like 6, TUFM, PGAM1, GCTG, ENO, NUAK1, PP2C, RNF34, PIGW, FDPS, MOGAT2, Forkhead box protein D4-like 6, NF-kB, HIST1H2AK, TADA1L, BTG2, SETDB1, CPA5, IGF2BP3, CLK1, F264, CGAT2, CHKB, GKP3, ZNF431, HIST2H2BE, RelB (NF-kB subunit), FosB/JunB, PCYOX1, RAD23B, TAFs, PCPH, Calmodulin, ZNF479, DIRAS2, NUP107, PDHA (testis), CaMK II gamma, ZNF708, MSK2, MCM2, ATP5G1, Histone H3.1, GMPS, HSC70, POLD reg (p50), ZNF791, Factor B, FEN1, ZNF226, H protein N6-dihydrolipoyllysine, IRE1, ERO1, ALY, PPIAL4, CG beta, Cyclin T2, Ataxin-7, ZNF678, C14orf126, RHO6, Thyroid hormone receptor, PMP34, VARSL, Histone H3, Aggrecanase-1, WAC, POLD cat (p125), HSP60, SOD1, GLUT4, mTERFD2, TCP1-zeta-1, GINS2, PUS7, PDH alpha, MRPL12, MED29, c-Fos, ZNF213, MOGAT3, ASXL3, MCM5, GLT25D1, PLC-beta, JunD, DPH2, UBE2G1, EBP, Ephrin-A, HSP90, SEH1L, DPY30, Gemin5, RPP40, Calpain 1(mu), Zinc finger protein 394, EWS/FLI1 fusion protein, RBM14, PRDX4, Histone H2BO, SMVT, ZNF674, NOLA2 (NHP2), ATP5C, C4A protein, JunB, TFIID 31 kDa subunit, TRIP13, C16orf42, HIST2H2AC, CHIP, Cullin 2, PLA2, CDK4, LPIN3, LDHA, UBE2S, GCSH, TFIIF, alpha subunit, HIST2H2AB, FOXD4L4, ERO1Lalpha, DNA polymerase nu, HIST1H2AH, NDPK A, FASTKD3, FUS/DDIT3 fusion protein, NDUFAB1, LSS, HAND2, DUSP8, Puromycin-sensitive aminopeptidase, DNAH1, GC1QBP, IPPK, SFRS1 (SF2), CERCAM, NeuN, NCOA3 (pCIP/SRC3), MafF, NPEPPS, FANCD2, HSP70, PUR9, EAR2, WIP1, RUVBL2, JMJD3, POLE3 (YBL1), NUP98/HHEX fusion protein, Histone H2A.o, Rod1, hnRNP C, DHFRL1, Ndg2, Utp15, MRPL19, HXK2, ATP5G, NDPK complex, RalA, FASN, Tyk2, SNRPD1 (SMD1), Forkhead box protein D4-like 6, FOXD4, USP52, PAFAH alpha (LIS1), ING3, CISY, PP1-cat gamma, EWS/ER81 fusion protein, ATF-3, MUS81, HEXIM1, OLAH, RAD1, Rad51, WDR5, C1TC, TTLL12, CGB, NS1-BP, TFIIE, beta subunit, CHID1, GTF2IRD2, SNRP116, PDSS1, ZNF813, 4EHP, Rab-3, SUV420H1, DHCR7, TRAP-1, RBBP7 (RbAp46), TRAP95, C/EBPbeta, RAI, GTPBP3, AMPL, MRPL18, IARS2, SUPT16H, ALDOC, PLC-beta3, DZIP3, Leptin, ACAT2, WDR61, Probable D-tyrosyl-tRNA(T, Claspin, HIST1H2BN, SAKS1, p66alpha, S-acyl fatty acid synthase thioesterase, medium chain, cPLA2, C4B protein, LEO1, HIST1H2AE, nNOS, HPRT, ERAP140, HMGCL, CNOT3, Actin, p19, NUDT5, TCP1-zeta-1/2, ZNF454, PP1-cat, Rac3, Forkhead box protein D4-like 6, eIF1A, SERB, Histone H2, HP1 gamma, JAK3, PPIL3, URI, Histone H2A, WRN, DHFR, ANAPC5, Elk-1, B3gnt8, ZFP36(Tristetraprolin), c-Jun/c-Jun, PAI1, Splicing factor 4, PHF13, Histone H1.2, snRNP-F, CHCHD4, C4, FTSJ1, MAZ, Foxd4L1, ADSL, RPP30, NDPK B, MDH1, NOM1, PSMA2, NIPK, HP1, UBL4A, FBXO10, PUR6, RBM5, Forkhead box protein D4-like 2, SBNO2, JPO1, PLCE, FAST kinase domain-containing protein 3, RRM1, F-box only protein 10, NUP37, SAP25, Dcp1b, Nucleolar protein 8, DHX15, C1s, SET, Histone H2B, EWS/ERG fusion protein, Trp53inp2, DDX46, CNT2, KHK, Rab-3A, FOXD4L5, DNAJB2, FLAD1, SerRS, ZNF721, PYCS, PUR1, Tubulin alpha 3, SNRPA, PAK2, TBR1, TCOF1, NF-kB p52/RelB, Acyl-CoA synthetase, FLJ10292, MRPL17, ZNF394, MUSK, HIST1H3D, GSPT1, Forkhead box protein D4-like 6, eIF4A, Histone H4, PP2C gamma, MPP alpha, RCOR3, LCMR1, TTBK2, CPT-1B, KAD6, DHODH, NUP98, ATP13A3, BTG1, SF4, SERTAD1, ROCK1, ACSL3, NIP3, TBDN100, CYHR1, KLF6, CENPP, EML3, TBX20, MAT2A, Betacellulin, NUP98/HOXA9 fusion protein, Cofilin, c-Jun, Neuroglycan C, HIST1H2AD, C1orf69, AARS, Actin muscle, SOX30, Frataxin, Phosphatase regulator (inhibitor), BTN2A1, Octanoyl-holo-acp, p23 co-chaperone, BCCIP, HIST1H2AJ, CENP-H, HERP, Ig light chain, Protein kinase G, Pc2, Ran, Tubulin alpha 3E, Glycosyltransferase 25 family member 3 precursor, NUP188, RLI, Ebp1, POLN, PGAM4, Histone H1, SP3, Ca-ATPase2, TREX2, NEUR1, Aciculin, NUP160, USP5, Histone H1.3, PUR4, Cyclophilin A, ASGR2, CDK2, 3'HEXO, KLHDC3, SOCS1, SET/NUP214 fusion protein, Tubulin (in microtubules), TCP1-epsilon, CDC45L, SLC25A1, RPIA, 14-3-3, VEGF-B
7	organic cyclic compound metabolic process	5562	9.25E-40	6.84E-37	9.25E-40	6.84E-37	424	DNA2, ZNF668, PACT, Forkhead box protein D4-like 2, GTF2IRD2B, PHAP1 (pp32), RFC4, BTEB4, LOC440093, ZSCAN4, dUTPase (DUT), CTP synthase, POLE3-POLE4 complex, Peroxiredoxin, Tubulin beta, CAPER, MIWI, TRUB2, FIGNL1, FUBP1, Ku70/80, REV-ERBalpha, IRF7, Dyskerin (NAP57), TK2, NOL8, TR-alpha, SC4MOL, hnRNP U, CREB1, PCNA, JMJD2A, ZNF441, Probable cation-transporting ATPase 13A3, C/EBP, MCM6, Histone H2AC, Ku80, Urocortin 2, SNRN25, Tip60, GRINL1A, PERC, Sen34, C5orf41, Tubulin alpha-1B, HMGI/Y, CAD, TER, FARSB, hnRNP H3, IKBZ, Cyclophilin H, GADD45 alpha, Chk2, YB-1, DDX54, PSMB3, MCM3, EGR1, RNPS1, PUR2, ACTC, MRM1, PTOP, HAT1, ABCC4, RBM6, HSP90 alpha, INTS6, RAD18, ING4, SSRP1, G3BP1 (hdhVIII), PSMD1, Forkhead box protein D4-like 2, SLA/LP, JOSD3, Ku70, PSMD2, C/EBPgamma, HIST1H2BD, EGR2 (Krox20), Casein kinase I, c-Jun/c-Fos, ROCK, PP2A regulatory, FASP1, ZNF425, CARM1, Importin (karyopherin)-beta, DDB1, ZNF561, Dynein, axonemal, heavy chains, EAF1, EWS, JunD/c-Fos, HIST1H2BG, Histone H3.3, TFE3, Serotonin receptor, EWS/WT1 fusion protein, C1orf124, HIST2H2BC, XTP3TPA, EPC1, ChAF1 subunit B, ACACA, PAXIP1L, PTMA, hnRNP R, hnRNP L, NAP2, RNF12, HIST2H2BF, RFX3, PSMC6, XRCC2, ZBTB10, ZNF491, AP-1, Galpha(i)-specific amine GPCRs, AKD1, SLBP, E2N(UBC13), ACLY, PSMD14, PAF49, NF-kB2 (p100), TXNIP (VDUP1), WARS, KLF2, APLF, GADD45 gamma, ATP1A1, TALDO, TARDBP (TDP43), C17orf79, APOA2, SLC19A1, eIF4A2, PSMB7, EWS/E1AF fusion protein, Cyclin L1, RPA1, C10orf119, LSM2, ENO1, Tubulin beta 1, C/EBP zeta, FBI-1 (Pokemon), WDR77, STRA13, HDAC3, Forkhead box protein D4-like 2, Tubulin alpha, ARD1, Rad52, MCPIP, HIST1H2BL, NOR1, ALDOA, POLR3H, HIST1H2BJ, MVD, Forkhead box protein D4-like 6, TUFM, ENO, PP2C, FDPS, Forkhead box protein D4-like 6, NF-kB, HIST1H2AK, TADA1L, BTG2, SETDB1, ZNF431, HIST2H2BE, RelB (NF-kB subunit), FosB/JunB, RAD23B, TAFs, PCPH, ZNF479, DIRAS2, ZNF708, MCM2, ATP5G1, Histone H3.1, GMPS, HSC70, POLD reg (p50), ZNF791, FEN1, ZNF226, IRE1, ALY, Cyclin T2, Ataxin-7, ZNF678, RHO6, Thyroid hormone receptor, VARSL, Histone H3, WAC, POLD cat (p125), HSP60, SOD1, mTERFD2, GINS2, PUS7, MRPL12, MED29, c-Fos, ZNF213, ASXL3, MCM5, PLC-beta, JunD, EBP, HSP90, DPY30, Gemin5, RPP40, Zinc finger protein 394, EWS/FLI1 fusion protein, RBM14, Histone H2BO, SMVT, ZNF674, NOLA2 (NHP2), ATP5C, JunB, TFIID 31 kDa subunit, TRIP13, C16orf42, HIST2H2AC, CHIP, LDHA, TFIIF, alpha subunit, HIST2H2AB, FOXD4L4, DNA polymerase nu, HIST1H2AH, NDPK A, FUS/DDIT3 fusion protein, LSS, HAND2, DNAH1, SFRS1 (SF2), NeuN, NCOA3 (pCIP/SRC3), MafF, FANCD2, HSP70, PUR9, EAR2, RUVBL2, POLE3 (YBL1), NUP98/HHEX fusion protein, Histone H2A.o, Rod1, hnRNP C, DHFRL1, Ndg2, Utp15, ATP5G, NDPK complex, RalA, SNRPD1 (SMD1), Forkhead box protein D4-like 6, SLC25A39, FOXD4, USP52, ING3, EWS/ER81 fusion protein, ATF-3, MUS81, HEXIM1, RAD1, Rad51, WDR5, C1TC, NS1-BP, TFIIE, beta subunit, GTF2IRD2, SNRP116, ZNF813, Rab-3, SUV420H1, DHCR7, RBBP7 (RbAp46), TRAP95, C/EBPbeta, RAI, GTPBP3, IARS2, SUPT16H, Leptin, WDR61, Claspin, HIST1H2BN, p66alpha, LEO1, HIST1H2AE, HPRT, ERAP140, CNOT3, Actin, p19, NUDT5, ZNF454, Rac3, Forkhead box protein D4-like 6, Histone H2, HP1 gamma, PPIL3, URI, Histone H2A, WRN, DHFR, Elk-1, ZFP36(Tristetraprolin), c-Jun/c-Jun, PAI1, Splicing factor 4, PHF13, Histone H1.2, snRNP-F, FTSJ1, MAZ, Foxd4L1, ADSL, RPP30, NDPK B, MDH1, NOM1, PSMA2, NIPK, HP1, PUR6, RBM5, Forkhead box protein D4-like 2, SBNO2, JPO1, RRM1, SAP25, Dcp1b, Nucleolar protein 8, DHX15, SET, Histone H2B, EWS/ERG fusion protein, Trp53inp2, DDX46, CNT2, Rab-3A, FOXD4L5, FLAD1, SerRS, ZNF721, PYCS, PUR1, Tubulin alpha 3, SNRPA, TBR1, TCOF1, NF-kB p52/RelB, FLJ10292, ZNF394, HIST1H3D, GSPT1, Forkhead box protein D4-like 6, eIF4A, Histone H4, RCOR3, LCMR1, KAD6, DHODH, NUP98, ATP13A3, SF4, SERTAD1, NIP3, TBDN100, KLF6, CENPP, TBX20, MAT2A, Betacellulin, NUP98/HOXA9 fusion protein, c-Jun, HIST1H2AD, C1orf69, AARS, Actin muscle, SOX30, Frataxin, Phosphatase regulator (inhibitor), p23 co-chaperone, BCCIP, HIST1H2AJ, CENP-H, Pc2, Ran, Tubulin alpha 3E, RLI, Ebp1, POLN, Histone H1, SP3, Ca-ATPase2, TREX2, Histone H1.3, PUR4, Cyclophilin A, CDK2, 3'HEXO, KLHDC3, SET/NUP214 fusion protein, Tubulin (in microtubules), CDC45L, RPIA, 14-3-3
8	cellular aromatic compound metabolic process	5352	1.06E-39	6.84E-37	1.06E-39	6.84E-37	413	DNA2, ZNF668, PACT, Forkhead box protein D4-like 2, GTF2IRD2B, PHAP1 (pp32), RFC4, BTEB4, LOC440093, ZSCAN4, dUTPase (DUT), CTP synthase, POLE3-POLE4 complex, Peroxiredoxin, Tubulin beta, CAPER, MIWI, TRUB2, FIGNL1, FUBP1, Ku70/80, REV-ERBalpha, IRF7, Dyskerin (NAP57), TK2, NOL8, TR-alpha, hnRNP U, CREB1, PCNA, JMJD2A, ZNF441, Probable cation-transporting ATPase 13A3, C/EBP, MCM6, Histone H2AC, Ku80, Urocortin 2, SNRN25, Tip60, GRINL1A, PERC, Sen34, C5orf41, Tubulin alpha-1B, HMGI/Y, CAD, FARSB, hnRNP H3, IKBZ, Cyclophilin H, GADD45 alpha, Chk2, YB-1, DDX54, PSMB3, MCM3, EGR1, RNPS1, PUR2, ACTC, MRM1, PTOP, HAT1, ABCC4, RBM6, HSP90 alpha, INTS6, RAD18, ING4, SSRP1, G3BP1 (hdhVIII), PSMD1, Forkhead box protein D4-like 2, SLA/LP, JOSD3, Ku70, PSMD2, C/EBPgamma, HIST1H2BD, EGR2 (Krox20), Casein kinase I, c-Jun/c-Fos, ROCK, PP2A regulatory, FASP1, ZNF425, CARM1, Importin (karyopherin)-beta, DDB1, ZNF561, Dynein, axonemal, heavy chains, EAF1, EWS, JunD/c-Fos, HIST1H2BG, Histone H3.3, TFE3, Serotonin receptor, EWS/WT1 fusion protein, C1orf124, HIST2H2BC, XTP3TPA, EPC1, ChAF1 subunit B, ACACA, PAXIP1L, PTMA, hnRNP R, hnRNP L, NAP2, RNF12, HIST2H2BF, RFX3, PSMC6, XRCC2, ZBTB10, ZNF491, AP-1, Galpha(i)-specific amine GPCRs, AKD1, SLBP, E2N(UBC13), ACLY, PSMD14, PAF49, NF-kB2 (p100), TXNIP (VDUP1), WARS, KLF2, APLF, GADD45 gamma, ATP1A1, TALDO, TARDBP (TDP43), C17orf79, SLC19A1, eIF4A2, PSMB7, EWS/E1AF fusion protein, Cyclin L1, RPA1, C10orf119, LSM2, ENO1, Tubulin beta 1, C/EBP zeta, FBI-1 (Pokemon), WDR77, STRA13, HDAC3, Forkhead box protein D4-like 2, Tubulin alpha, ARD1, Rad52, MCPIP, HIST1H2BL, NOR1, ALDOA, POLR3H, HIST1H2BJ, Forkhead box protein D4-like 6, TUFM, ENO, PP2C, Forkhead box protein D4-like 6, NF-kB, HIST1H2AK, TADA1L, BTG2, SETDB1, ZNF431, HIST2H2BE, RelB (NF-kB subunit), FosB/JunB, RAD23B, TAFs, PCPH, ZNF479, DIRAS2, ZNF708, MCM2, ATP5G1, Histone H3.1, GMPS, HSC70, POLD reg (p50), ZNF791, FEN1, ZNF226, IRE1, ALY, Cyclin T2, Ataxin-7, ZNF678, RHO6, Thyroid hormone receptor, VARSL, Histone H3, WAC, POLD cat (p125), HSP60, SOD1, mTERFD2, GINS2, PUS7, MRPL12, MED29, c-Fos, ZNF213, ASXL3, MCM5, PLC-beta, JunD, HSP90, DPY30, Gemin5, RPP40, Zinc finger protein 394, EWS/FLI1 fusion protein, RBM14, Histone H2BO, ZNF674, NOLA2 (NHP2), ATP5C, JunB, TFIID 31 kDa subunit, TRIP13, C16orf42, HIST2H2AC, CHIP, LDHA, TFIIF, alpha subunit, HIST2H2AB, FOXD4L4, DNA polymerase nu, HIST1H2AH, NDPK A, FUS/DDIT3 fusion protein, HAND2, DNAH1, SFRS1 (SF2), NeuN, NCOA3 (pCIP/SRC3), MafF, FANCD2, HSP70, PUR9, EAR2, RUVBL2, POLE3 (YBL1), NUP98/HHEX fusion protein, Histone H2A.o, Rod1, hnRNP C, DHFRL1, Ndg2, Utp15, ATP5G, NDPK complex, RalA, SNRPD1 (SMD1), Forkhead box protein D4-like 6, SLC25A39, FOXD4, USP52, ING3, EWS/ER81 fusion protein, ATF-3, MUS81, HEXIM1, RAD1, Rad51, WDR5, C1TC, NS1-BP, TFIIE, beta subunit, GTF2IRD2, SNRP116, ZNF813, Rab-3, SUV420H1, RBBP7 (RbAp46), TRAP95, C/EBPbeta, RAI, GTPBP3, IARS2, SUPT16H, WDR61, Claspin, HIST1H2BN, p66alpha, LEO1, HIST1H2AE, HPRT, ERAP140, CNOT3, Actin, p19, NUDT5, ZNF454, Rac3, Forkhead box protein D4-like 6, Histone H2, HP1 gamma, PPIL3, URI, Histone H2A, WRN, DHFR, Elk-1, ZFP36(Tristetraprolin), c-Jun/c-Jun, PAI1, Splicing factor 4, PHF13, Histone H1.2, snRNP-F, FTSJ1, MAZ, Foxd4L1, ADSL, RPP30, NDPK B, MDH1, NOM1, PSMA2, NIPK, HP1, PUR6, RBM5, Forkhead box protein D4-like 2, SBNO2, JPO1, RRM1, SAP25, Dcp1b, Nucleolar protein 8, DHX15, SET, Histone H2B, EWS/ERG fusion protein, Trp53inp2, DDX46, CNT2, Rab-3A, FOXD4L5, FLAD1, SerRS, ZNF721, PUR1, Tubulin alpha 3, SNRPA, TBR1, TCOF1, NF-kB p52/RelB, FLJ10292, ZNF394, HIST1H3D, GSPT1, Forkhead box protein D4-like 6, eIF4A, Histone H4, RCOR3, LCMR1, KAD6, DHODH, NUP98, ATP13A3, SF4, SERTAD1, NIP3, TBDN100, KLF6, CENPP, TBX20, MAT2A, Betacellulin, NUP98/HOXA9 fusion protein, c-Jun, HIST1H2AD, C1orf69, AARS, Actin muscle, SOX30, Frataxin, Phosphatase regulator (inhibitor), p23 co-chaperone, BCCIP, HIST1H2AJ, CENP-H, Pc2, Ran, Tubulin alpha 3E, RLI, Ebp1, POLN, Histone H1, SP3, Ca-ATPase2, TREX2, Histone H1.3, PUR4, Cyclophilin A, CDK2, 3'HEXO, KLHDC3, SET/NUP214 fusion protein, Tubulin (in microtubules), CDC45L, RPIA, 14-3-3
9	metabolic process	10691	1.17E-39	6.84E-37	1.17E-39	6.84E-37	661	[illegible] (reg), MIWI, TRUB2, FIGNL1, FUBP1, Ku70/80, REV-ERBalpha, IRF7, FAF1, Dyskerin (NAP57), C9orf105, TK2, NOL8, TR-alpha, SC4MOL, hnRNP U, CREB1, PCNA, PDK1, JMJD2A, ZNF441, Probable cation-transporting ATPase 13A3, GSTM2, C/EBP, TOM40, MCM6, IGKC, MGST3, CTE2, Histone H2AC, Ku80, Urocortin 2, SNRN25, Tip60, 14-3-3 gamma, H protein N6-lipoyllysine, EIF1AX, GRINL1A, PERC, UQCR11, Sen34, PLK3 (CNK), C5orf41, Tubulin alpha-1B, MANEA, HMGI/Y, CAD, TIGAR (C12orf5), TER, FARSB, hnRNP H3, IKBZ, Cyclophilin H, GADD45 alpha, , Chk2, YB-1, PGHD, FAST kinase domain-containing protein 3, FBXO8, DDX54, MIF, PSMB3, MCM3, eIF2B3, EGR1, RNPS1, PUR2, ACTC, MRM1, PTOP, HAT1, ABCC4, RBM6, HSP90 alpha, PLA2G4C, INTS6, RAD18, ING4, SSRP1, G3BP1 (hdhVIII), PSMD1, CSNK1G1, 14-3-3 epsilon, SRP72, MRPL4, NDUFA2, Forkhead box protein D4-like 2, SLA/LP, JOSD3, Ku70, PSMD2, C/EBPgamma, HIST1H2BD, EGR2 (Krox20), Casein kinase I, c-Jun/c-Fos, ROCK, PP2A regulatory, NDUFB2, FASP1, ZNF425, CARM1, Importin (karyopherin)-beta, DDB1, ZNF561, MSK1/2 (RPS6KA5/4), Dynein, axonemal, heavy chains, GLUT1, C13orf39, EAF1, EWS, PRMT1, JunD/c-Fos, HIST1H2BG, Skp2/TrCP/FBXW, Histone H3.3, TFE3, HSP10 (mitochondrial), Serotonin receptor, EWS/WT1 fusion protein, C1orf124, PREI4, MSTP9, HIST2H2BC, MTMR4, XTP3TPA, NUP43, EPC1, NUP205, STK19, ChAF1 subunit B, ACACA, COX VIb-1, PAXIP1L, MSP, PTMA, TCP1-delta, UQCR10, hnRNP R, hnRNP L, NAP2, RNF12, HIST2H2BF, RFX3, PSMC6, 4E-BP2, XRCC2, SLC25A10, Kappa chain (Ig light chain), ZBTB10, ZNF491, NSUN3, UBC7, AP-1, HSST4, IBRDC3, Galpha(i)-specific amine GPCRs, AKD1, TLK2, SLBP, E2N(UBC13), Protein kinase G1, COQ4, KIAA0152, ACLY, PSMD14, PAF49, NF-kB2 (p100), B4GT2, TXNIP (VDUP1), WARS, KLF2, CDK5, APLF, GADD45 gamma, ATP1A1, TALDO, TARDBP (TDP43), CRIg, Acsm5, C17orf79, ISOC1, MMP-8, APOA2, SPSY, COX Va, SLC19A1, CGI-51, eIF4A2, PSMB7, EWS/E1AF fusion protein, Cyclin L1, RPA1, SKP2, C10orf119, NDUFV2, LSM2, ENO1, Tubulin beta 1, C/EBP zeta, FBI-1 (Pokemon), WDR77, Galpha(q)-specific peptide GPCRs, STRA13, HDAC3, Forkhead box protein D4-like 2, Tubulin alpha, ARD1, Rad52, Cofilin, non-muscle, MCPIP, HIST1H2BL, NOR1, ALDOA, POLR3H, HIST1H2BJ, GLRX5, MVD, Forkhead box protein D4-like 6, TUFM, PIG8, PGAM1, GCTG, ENO, NUAK1, PP2C, RNF34, PIGW, FDPS, MOGAT2, Forkhead box protein D4-like 6, NF-kB, HIST1H2AK, TADA1L, BTG2, SETDB1, CPA5, IGF2BP3, CLK1, F264, CGAT2, CHKB, GKP3, ZNF431, HIST2H2BE, RelB (NF-kB subunit), FosB/JunB, PCYOX1, RAD23B, TAFs, PCPH, Calmodulin, ZNF479, DIRAS2, NUP107, PDHA (testis), CaMK II gamma, ZNF708, MSK2, SPEE, MCM2, ATP5G1, Histone H3.1, GMPS, HSC70, LRGUK, POLD reg (p50), ZNF791, Factor B, FEN1, ZNF226, H protein N6-dihydrolipoyllysine, IRE1, ERO1, ALY, PPIAL4, CG beta, Cyclin T2, Ataxin-7, ZNF678, C14orf126, RHO6, Thyroid hormone receptor, PMP34, VARSL, Histone H3, Aggrecanase-1, WAC, POLD cat (p125), HSP60, SOD1, GLUT4, mTERFD2, TCP1-zeta-1, GINS2, PUS7, PDH alpha, MRPL12, MED29, c-Fos, ZNF213, MOGAT3, ASXL3, MCM5, MAP1LC3B, GLT25D1, PLC-beta, JunD, DPH2, UBE2G1, EBP, Ephrin-A, HSP90, ACAD-9, SEH1L, DPY30, Gemin5, RPP40, Calpain 1(mu), Zinc finger protein 394, EWS/FLI1 fusion protein, RBM14, PRDX4, Histone H2BO, SMVT, ZNF674, NOLA2 (NHP2), ATP5C, C4A protein, FUMH, JunB, TFIID 31 kDa subunit, TRIP13, C16orf42, HIST2H2AC, CHIP, Cullin 2, PLA2, CDK4, LPIN3, LDHA, UBE2S, UQCRQPC, GCSH, TFIIF, alpha subunit, HIST2H2AB, FOXD4L4, UQCRH, ERO1Lalpha, DNA polymerase nu, HIST1H2AH, NDPK A, FASTKD3, FUS/DDIT3 fusion protein, NDUFAB1, LSS, HAND2, DUSP8, Puromycin-sensitive aminopeptidase, NDUFA1, Myosin I, DNAH1, GC1QBP, MYO1H, IPPK, SFRS1 (SF2), CERCAM, NeuN, NCOA3 (pCIP/SRC3), MafF, NPEPPS, FANCD2, HSP70, PUR9, EAR2, WIP1, RUVBL2, JMJD3, POLE3 (YBL1), NUP98/HHEX fusion protein, Histone H2A.o, Rod1, hnRNP C, DHFRL1, Ndg2, Utp15, MRPL19, HXK2, ATP5G, Exportin 5, NDPK complex, RalA, FASN, Tyk2, SNRPD1 (SMD1), Forkhead box protein D4-like 6, SLC25A39, FOXD4, USP52, PAFAH alpha (LIS1), ING3, CISY, PP1-cat gamma, EWS/ER81 fusion protein, ATF-3, MUS81, HEXIM1, OLAH, RAD1, Thioredoxin-like 2, Rad51, WDR5, C1TC, TTLL12, CGB, NS1-BP, TFIIE, beta subunit, COX17, CHID1, GTF2IRD2, SNRP116, PDSS1, ZNF813, 4EHP, Rab-3, SUV420H1, DHCR7, TRAP-1, RBBP7 (RbAp46), TRAP95, C/EBPbeta, RAI, GTPBP3, AMPL, MRPL18, IARS2, SUPT16H, ALDOC, PLC-beta3, HA95, IL-8, DZIP3, Leptin, ACAT2, WDR61, Probable D-tyrosyl-tRNA(T, Claspin, HIST1H2BN, SAKS1, p66alpha, S-acyl fatty acid synthase thioesterase, medium chain, cPLA2, C4B protein, LEO1, HIST1H2AE, nNOS, HPRT, ERAP140, HMGCL, CNOT3, COX VIa, Actin, p19, NUDT5, TCP1-zeta-1/2, ZNF454, PP1-cat, VKORC1L1, Rac3, Forkhead box protein D4-like 6, eIF1A, SERB, Histone H2, DHRS13, HP1 gamma, JAK3, PPIL3, URI, Histone H2A, WRN, DHFR, ANAPC5, Elk-1, B3gnt8, ZFP36(Tristetraprolin), c-Jun/c-Jun, PAI1, Splicing factor 4, PHF13, NDUFS6, Histone H1.2, snRNP-F, CHCHD4, C4, FTSJ1, MAZ, Foxd4L1, ADSL, COX VIb, RPP30, NDPK B, MDH1, NOM1, SIN1, PSMA2, NIPK, HP1, UBL4A, FBXO10, PUR6, RBM5, Forkhead box protein D4-like 2, SBNO2, JPO1, Galpha(s)-specific glucagon GPCRs, PLCE, FAST kinase domain-containing protein 3, RRM1, F-box only protein 10, NUP37, SAP25, Dcp1b, Nucleolar protein 8, DHX15, C1s, SET, Histone H2B, ALKBH5, FAM20B, EWS/ERG fusion protein, Trp53inp2, DDX46, CNT2, KHK, Rab-3A, FOXD4L5, DNAJB2, FLAD1, SerRS, ZNF721, PYCS, PUR1, Tubulin alpha 3, SNRPA, PAK2, TBR1, TCOF1, NF-kB p52/RelB, Acyl-CoA synthetase, FLJ10292, MRPL17, ZNF394, MUSK, HIST1H3D, GSPT1, Forkhead box protein D4-like 6, eIF4A, Histone H4, CD147, PP2C gamma, COX Vb, MPP alpha, RCOR3, LCMR1, COX VIa-1, TTBK2, CPT-1B, KAD6, DHODH, NUP98, ATP13A3, BTG1, SF4, SERTAD1, ROCK1, ACSL3, NIP3, TBDN100, CYHR1, KLF6, CENPP, EML3, TBX20, MAT2A, Betacellulin, NUP98/HOXA9 fusion protein, Cofilin, c-Jun, Neuroglycan C, HIST1H2AD, C1orf69, AARS, Actin muscle, SOX30, Frataxin, Phosphatase regulator (inhibitor), BTN2A1, Octanoyl-holo-acp, p23 co-chaperone, BCCIP, HIST1H2AJ, CENP-H, HERP, Ig light chain, Protein kinase G, Pc2, Ran, Tubulin alpha 3E, Glycosyltransferase 25 family member 3 precursor, NUP188, RLI, Ebp1, POLN, PGAM4, Histone H1, SP3, Ca-ATPase2, TREX2, NEUR1, Aciculin, NUP160, USP5, Histone H1.3, PUR4, Cyclophilin A, ASGR2, CDK2, 3'HEXO, KLHDC3, SOCS1, SET/NUP214 fusion protein, Tubulin (in microtubules), TCP1-epsilon, CDC45L, SLC25A1, RPIA, 14-3-3, VEGF-B
10	organic substance metabolic process	6141	3.2E-36	1.54E-33	3.2E-36	1.54E-33	444	DNA2, ZNF668, PACT, Forkhead box protein D4-like 2, GTF2IRD2B, PHAP1 (pp32), RFC4, BTEB4, LOC440093, ZSCAN4, dUTPase (DUT), CTP synthase, POLE3-POLE4 complex, Peroxiredoxin, Tubulin beta, CAPER, DPM2 (reg), MIWI, TRUB2, FIGNL1, FUBP1, Ku70/80, REV-ERBalpha, IRF7, Dyskerin (NAP57), TK2, NOL8, TR-alpha, SC4MOL, hnRNP U, CREB1, PCNA, JMJD2A, ZNF441, Probable cation-transporting ATPase 13A3, C/EBP, MCM6, Histone H2AC, Ku80, Urocortin 2, SNRN25, Tip60, GRINL1A, PERC, Sen34, C5orf41, Tubulin alpha-1B, MANEA, HMGI/Y, CAD, TER, FARSB, hnRNP H3, IKBZ, Cyclophilin H, GADD45 alpha, Chk2, YB-1, DDX54, PSMB3, MCM3, EGR1, RNPS1, PUR2, ACTC, MRM1, PTOP, HAT1, ABCC4, RBM6, HSP90 alpha, INTS6, RAD18, ING4, SSRP1, G3BP1 (hdhVIII), PSMD1, Forkhead box protein D4-like 2, SLA/LP, JOSD3, Ku70, PSMD2, C/EBPgamma, HIST1H2BD, EGR2 (Krox20), Casein kinase I, c-Jun/c-Fos, ROCK, PP2A regulatory, FASP1, ZNF425, CARM1, Importin (karyopherin)-beta, DDB1, ZNF561, Dynein, axonemal, heavy chains, EAF1, EWS, JunD/c-Fos, HIST1H2BG, Histone H3.3, TFE3, Serotonin receptor, EWS/WT1 fusion protein, C1orf124, HIST2H2BC, XTP3TPA, EPC1, ChAF1 subunit B, ACACA, PAXIP1L, PTMA, hnRNP R, hnRNP L, NAP2, RNF12, HIST2H2BF, RFX3, PSMC6, XRCC2, ZBTB10, ZNF491, AP-1, HSST4, Galpha(i)-specific amine GPCRs, AKD1, SLBP, E2N(UBC13), KIAA0152, ACLY, PSMD14, PAF49, NF-kB2 (p100), B4GT2, TXNIP (VDUP1), WARS, KLF2, APLF, GADD45 gamma, ATP1A1, TALDO, TARDBP (TDP43), C17orf79, APOA2, SLC19A1, eIF4A2, PSMB7, EWS/E1AF fusion protein, Cyclin L1, RPA1, C10orf119, LSM2, ENO1, Tubulin beta 1, C/EBP zeta, FBI-1 (Pokemon), WDR77, STRA13, HDAC3, Forkhead box protein D4-like 2, Tubulin alpha, ARD1, Rad52, MCPIP, HIST1H2BL, NOR1, ALDOA, POLR3H, HIST1H2BJ, MVD, Forkhead box protein D4-like 6, TUFM, ENO, PP2C, FDPS, Forkhead box protein D4-like 6, NF-kB, HIST1H2AK, TADA1L, BTG2, SETDB1, F264, CGAT2, CHKB, ZNF431, HIST2H2BE, RelB (NF-kB subunit), FosB/JunB, RAD23B, TAFs, PCPH, ZNF479, DIRAS2, ZNF708, MCM2, ATP5G1, Histone H3.1, GMPS, HSC70, POLD reg (p50), ZNF791, FEN1, ZNF226, IRE1, ALY, Cyclin T2, Ataxin-7, ZNF678, RHO6, Thyroid hormone receptor, VARSL, Histone H3, Aggrecanase-1, WAC, POLD cat (p125), HSP60, SOD1, GLUT4, mTERFD2, GINS2, PUS7, MRPL12, MED29, c-Fos, ZNF213, ASXL3, MCM5, PLC-beta, JunD, EBP, HSP90, DPY30, Gemin5, RPP40, Zinc finger protein 394, EWS/FLI1 fusion protein, RBM14, Histone H2BO, SMVT, ZNF674, NOLA2 (NHP2), ATP5C, JunB, TFIID 31 kDa subunit, TRIP13, C16orf42, HIST2H2AC, CHIP, PLA2, LPIN3, LDHA, TFIIF, alpha subunit, HIST2H2AB, FOXD4L4, DNA polymerase nu, HIST1H2AH, NDPK A, FUS/DDIT3 fusion protein, LSS, HAND2, DNAH1, SFRS1 (SF2), NeuN, NCOA3 (pCIP/SRC3), MafF, FANCD2, HSP70, PUR9, EAR2, RUVBL2, POLE3 (YBL1), NUP98/HHEX fusion protein, Histone H2A.o, Rod1, hnRNP C, DHFRL1, Ndg2, Utp15, ATP5G, NDPK complex, RalA, SNRPD1 (SMD1), Forkhead box protein D4-like 6, SLC25A39, FOXD4, USP52, ING3, EWS/ER81 fusion protein, ATF-3, MUS81, HEXIM1, RAD1, Rad51, WDR5, C1TC, NS1-BP, TFIIE, beta subunit, CHID1, GTF2IRD2, SNRP116, ZNF813, Rab-3, SUV420H1, DHCR7, RBBP7 (RbAp46), TRAP95, C/EBPbeta, RAI, GTPBP3, IARS2, SUPT16H, ALDOC, Leptin, WDR61, Claspin, HIST1H2BN, p66alpha, LEO1, HIST1H2AE, HPRT, ERAP140, CNOT3, Actin, p19, NUDT5, ZNF454, Rac3, Forkhead box protein D4-like 6, Histone H2, HP1 gamma, PPIL3, URI, Histone H2A, WRN, DHFR, Elk-1, B3gnt8, ZFP36(Tristetraprolin), c-Jun/c-Jun, PAI1, Splicing factor 4, PHF13, Histone H1.2, snRNP-F, FTSJ1, MAZ, Foxd4L1, ADSL, RPP30, NDPK B, MDH1, NOM1, PSMA2, NIPK, HP1, PUR6, RBM5, Forkhead box protein D4-like 2, SBNO2, JPO1, RRM1, SAP25, Dcp1b, Nucleolar protein 8, DHX15, SET, Histone H2B, EWS/ERG fusion protein, Trp53inp2, DDX46, CNT2, Rab-3A, FOXD4L5, FLAD1, SerRS, ZNF721, PYCS, PUR1, Tubulin alpha 3, SNRPA, TBR1, TCOF1, NF-kB p52/RelB, FLJ10292, ZNF394, HIST1H3D, GSPT1, Forkhead box protein D4-like 6, eIF4A, Histone H4, RCOR3, LCMR1, KAD6, DHODH, NUP98, ATP13A3, SF4, SERTAD1, NIP3, TBDN100, KLF6, CENPP, TBX20, MAT2A, Betacellulin, NUP98/HOXA9 fusion protein, c-Jun, Neuroglycan C, HIST1H2AD, C1orf69, AARS, Actin muscle, SOX30, Frataxin, Phosphatase regulator (inhibitor), p23 co-chaperone, BCCIP, HIST1H2AJ, CENP-H, Pc2, Ran, Tubulin alpha 3E, RLI, Ebp1, POLN, Histone H1, SP3, Ca-ATPase2, TREX2, NEUR1, Aciculin, Histone H1.3, PUR4, Cyclophilin A, ASGR2, CDK2, 3'HEXO, KLHDC3, SET/NUP214 fusion protein, Tubulin (in microtubules), CDC45L, RPIA, 14-3-3, VEGF-B
